# Comparative proteomics reveals unexpected quantitative phosphorylation differences linked to platelet activation state

**DOI:** 10.1038/s41598-019-55391-5

**Published:** 2019-12-12

**Authors:** G. J. Schmidt, C. M. Reumiller, H. Ercan, U. Resch, E. Butt, S. Heber, Z. Liutkevičiūte, J. Basílio, J. A. Schmid, A. Assinger, B. Jilma, M. Zellner

**Affiliations:** 1 Department of Clinical Pharmacology, Medical University of Vienna, Vienna, Austria; 2 Center for Physiology and Pharmacology, Institute of Vascular Biology and Thrombosis Research, Medical University of Vienna, Vienna, Austria; 3 Institute for Experimental Biomedicine II, University Clinic, Wuerzburg, Germany; 4 Center for Physiology and Pharmacology, Institute of Physiology, Medical University of Vienna, Vienna, Austria; 5 Center for Physiology and Pharmacology, Institute of Pharmacology, Medical University of Vienna, Vienna, Austria

**Keywords:** Phosphoproteins, Diagnostic markers, Molecular medicine

## Abstract

There is a need to assess platelet activation in patients with thrombotic disorders. P-selectin and activated integrin αIIbβ3 are usually quantified by flow cytometry to measure platelet activation. Monitoring changes in vasodilator-stimulated phosphoprotein (VASP) phosphorylation is an established method to determine the platelet-reactivity status. To study disruptions of platelet reactivity more comprehensively, we compared the human non-secretory platelet proteome after *in-vitro* -activation and –inhibition with their respective untreated controls using unbiased fluorescence two-dimensional differential in-gel electrophoresis. The non-secretory platelet proteome was more severely affected during inhibition than during activation. Strikingly, while VASP reached a 1.3-fold increase in phosphorylation levels in inhibited platelets, other protein kinase A targets showed several-fold stronger inhibition-induced phosphorylation levels, including LIM and SH3 domain protein 1 (6.7-fold), Src kinase-associated phosphoprotein 2 (4.6-fold), and Ras-related protein Rap1b (4.1-fold). Moreover, phosphorylation of integrin-linked protein kinase (ILK) and pleckstrin (PLEK) species was associated with P-selectin surface expression. The discrimination power between activation and inhibition was more pronounced for dephosphorylated ILK (3.79 Cohen’s d effect size) and phosphorylated PLEK (3.77) species than for P-selectin (2.35). These data reveal new insights into the quantitative changes of the platelet reactivity proteome and suggest powerful alternatives to characterise their activation and inactivation potential.

## Introduction

Platelets represent a decisive factor in (cardio)vascular diseases, the leading cause of death in the industrialised world. Indeed, inappropriate platelet reactivity and subsequent thrombus formation contribute to as many as 610,000 deaths per year in the USA^1^. In the circulation platelets are preserved in a sensitive balance between upholding adequate blood-flow and promoting blood clotting in the case of bleeding. Upon vessel injury, platelets instantly become activated, leading to granule release, aggregation, and clot formation. Although much is known about factors resulting in platelet activation, the molecular characteristics maintaining platelets in their quiescent state are less extensively characterized. Since dysregulation of platelet reactivity represents such a serious health hazard, it is necessary to explore biomarkers reflecting platelet activation status for their monitoring in health and disease.

Major *in-vivo* triggers for activation include binding of sub-endothelial collagen as well as other platelet agonists, like adenosine-diphosphate (ADP) and thrombin, to their cognate receptors^2,3^. At balanced physiological conditions, endothelial cells produce prostacyclin (PGI_2_) and nitric oxide, thereby keeping platelets silenced. Both mediators induce the synthesis of cyclic nucleotides (cAMP, cGMP), which activate protein kinases A and G (PKA, PKG) in platelets. These serine/threonine-kinases phosphorylate particular proteins to counteract the activation of platelets^4^. Platelet activation and thrombus formation can, therefore, be regarded as the predominance of either pro-thrombotic factors or a reduction of endogenous inhibitors^5^. An impairment^6^ of platelet-inhibition mechanisms, or resistance^7^ to them, may result in hyper-reactive platelets and spontaneous generation of platelet-dependent thrombosis^8,9^. Accordingly, typical platelet activation markers like P-selectin (CD62P) and activated integrin α_IIb_β_3_ (CD41/CD61) are significantly changed under these conditions and are thus used to assess the platelet activation status^10–12^. However, whether increased platelet activation results from an endogenous activation or from an insufficient inhibition remains largely unknown.

To investigate how the platelet proteome is affected by activation and inhibition in a comprehensive and unbiased manner, proteomics may be applied. Several *in-vitro* platelet proteomic studies have shed light on the complexity of signal propagation induced upon activation with thrombin receptor activating peptide-6 (TRAP-6)^13^, ADP^14^, collagen-related peptide^15^ or via the C-type lectin-like receptor 2^16^. Although an insufficient inhibition of platelets is also a significant feature in platelet-related thrombotic disorders, the first proteomic studies addressing this issue have been published just recently^17,18^. There, the dynamics of the phospho-proteome was analysed after short-term inhibition via selective stimulation of the sGC/cGMPIß (cyclic guanosine monophosphate/protein kinase GIß) pathway^18^, cyclic adenosine monophosphate/protein kinase A (cAMP/PKA) pathway^17^ or with simultaneous activation by ADP^19^. Nevertheless, a direct comparison of the platelet proteome response upon both activation and inhibition might uncover differential pathophysiological signs for hemostatic disorders. Protein profiles that reflect the activation status may enable us to target platelets pharmaceutically according to the underlying perturbations and thus to identify new therapeutic options.

Therefore, the aim of the present platelet proteome study was to reveal specific features of activated, as well as inhibited platelets, compared to their untreated controls within one biochemical method using fluorescence two-dimensional differential gel electrophoresis (2D-DIGE). This proteomics technique enables reliable quantitative results on differential protein expressions by displaying thousands of proteins, their isoforms and post-translational modifications at the same time. Previously, the terminology “protein species” has emerged to describe proteins which arise from the same amino acid sequence but have been post-translationally modified. The related term “proteoform” defines distinct protein forms originating from a single gene^20^. Especially, the generation of protein species may be of great interest in the proteome of anucleate platelets during activation and inhibition in the circulation.

Importantly, the concentrations of platelet activators and inhibitors needed to address these two main regulatory conditions were selected from typical protocols of functional platelet studies^21–24^. For platelet activation TRAP-6 (15 µM), was chosen to provoke a strong PAR1-mediated activation response while ADP (5 µM) induced a moderate one. For inactivation we used the unstable PGI_2_ (0.4 µM) and the more stable CTAD formulation (15 mM theophylline, 3.7 mM adenosine, 0.198 mM dipyridamole). Both inhibitory treatments especially increase cAMP to prevent platelet activation^25^.

Proteomic snapshots were taken after 15 minutes of activation/inhibition treatments to reveal long-lasting changes in platelet protein profiles, which may be useful for translation of these biomarkers into diagnostic applications. Notably, instead of the usual centrifugation step, proteins of *in-vitro* treated gel-filtered platelet suspension were extracted by TCA-precipitation^26^ to “freeze” them in their particular activation status. It was thanks to this method that we quantified the non-secretory platelet proteome changes since the granule release was not separated by this protein extraction protocol. Using 2D-DIGE, most of the affected proteoforms were identified to be specific to the either activated or inhibited platelet status. By quantifying their abundances, some of these proteins were found to be more powerful than established biomarkers in defining platelet reactivity.

## Results

### Quality control of platelet *in-vitro* models and 2D-DIGE analysis

**Figure 1:**
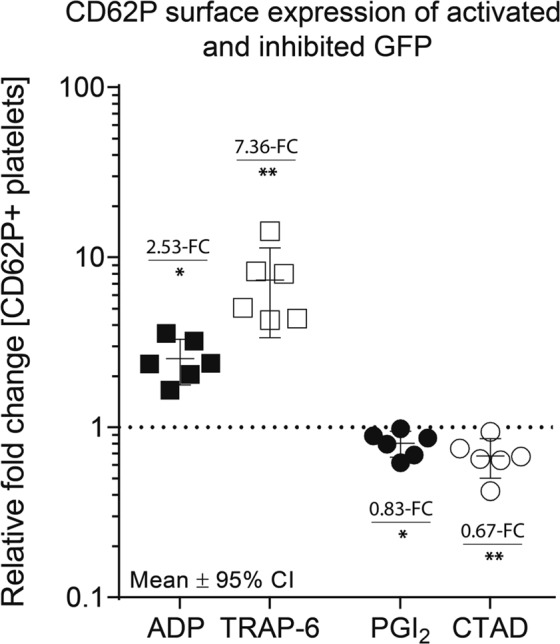
CD62P surface expression of activated and inhibited gel-filtered platelets. Activation status of platelets subjected to 2D-DIGE was determined by quantifying percentage of platelet CD62P- (P-selectin) surface expression using flow cytometry. Gel-filtered platelets treated with either ADP □, TRAP-6 ■, PGI_2_ ● or CTAD ○ were incubated for 15 minutes with a phycoerythrin (PE)-labelled antibody against CD62P. The CD62P-positive platelets of each individual are depicted as relative fold changes to untreated gel-filtered platelets (dashed line at y = 1). For each comparison, the average fold change (FC) and the significance level are given in the graph. The null hypothesis was tested by a one-sample t-test with an α-level set at 0.05. n = 6 (PGI_2_, CTAD), n = 6 (ADP, TRAP-6). *p < 0.05 **p < 0.01. *PGI*_2_ *- prostacyclin, CTAD - citrate, theophylline, adenosine, dipyridamole, ADP - adenosine-diphosphate, TRAP-6 – thrombin receptor-activating peptide-6*.

To assure that our platelet proteome analysis was assessed from well-defined *in-vitro* activated and inhibited platelets, CD62P, a surface marker specific for platelet activation, was quantified by flow cytometry. A strong platelet activation (>90% CD62P-positive platelets) was induced by TRAP-6 (15 µM) and a moderate response was provoked by ADP (5 µM, on average 50% CD62P-positive platelets), which is comparable to published data of platelet activation studies^27,28^. Accordingly, we found a 7.36-fold and 2.53-fold increase of CD62P-positive platelets, as compared to untreated controls, for TRAP-6 (p = 0.009) and ADP (p = 0.031), respectively (Fig. 1). These increases imply that platelet functionality was maintained after isolation. For the assessment of platelet inhibition, CD62P surface expression was also evaluated after CTAD and PGI_2_ (0.4 µM) treatment. Inhibited gel-filtered platelets showed a reduced CD62P positivity with 0.67-fold after CTAD- (p = 0.005) and of 0.83-fold after PGI_2_ treatment (p = 0.016) compared to untreated gel-filtered platelets.

We also monitored the quality of our 2D-DIGE proteome analysis regarding the biological and technical coefficient of variation (CV_tot_ = CV_bio_ + CV_tech_)^29^. In the present study, we determined a median CV_tot_ of 12.3% in the untreated control platelet proteome (5,906 spots) from all included volunteers, plus a single CV_tot_ of 5.7% for 14-3-3γ, a low biological variation protein, confirming a solid-technical quality when compared to our previous platelet proteomic studies^30^.

### Inhibition shows stronger changes than activation in the 2D-DIGE platelet proteome

**Figure 2:**
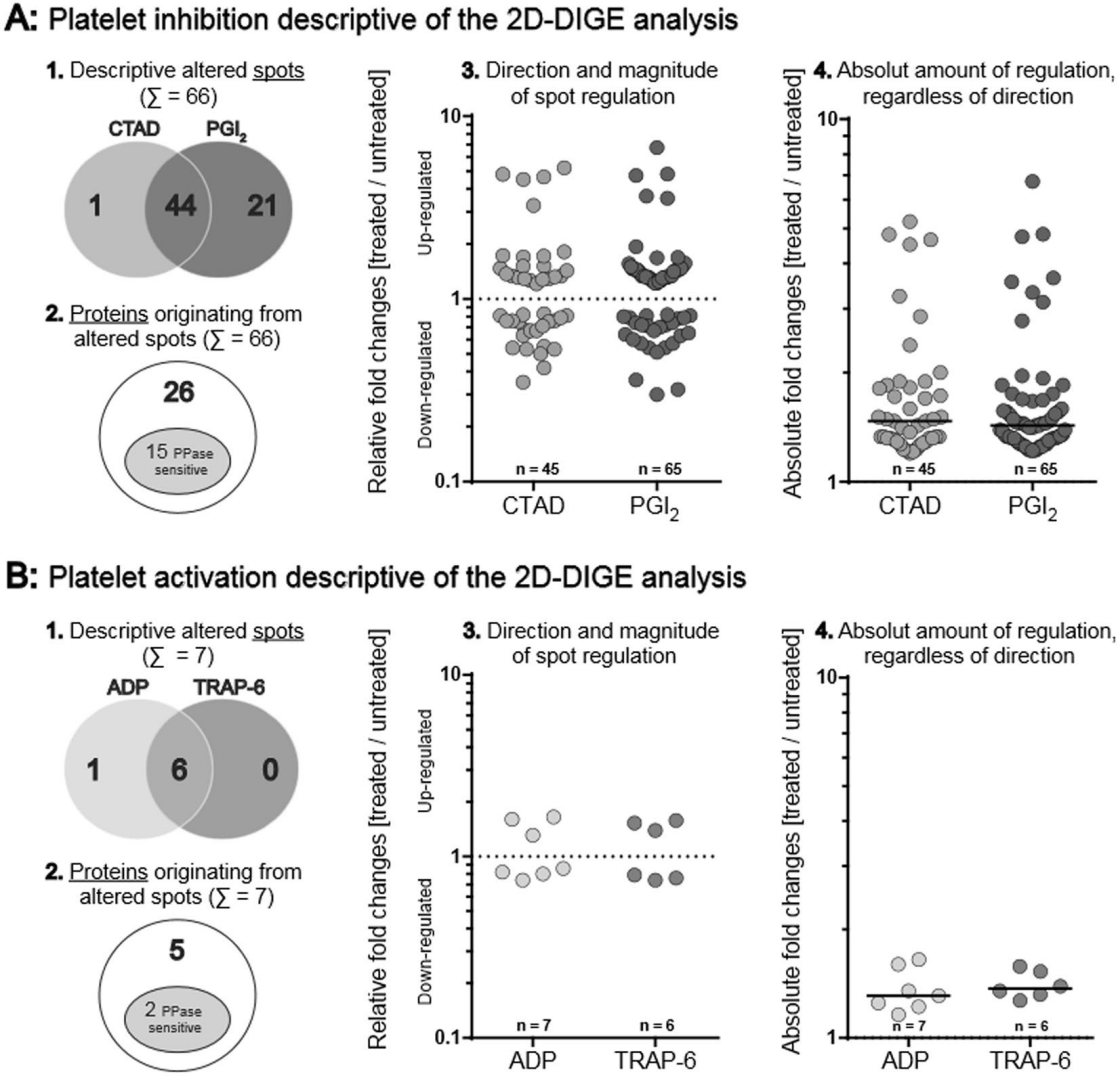
Quantitative and qualitative platelet proteome changes in response to (**A**) inhibition (CTAD/PGI_2_) and (**B**) activation (ADP/TRAP-6) compared to untreated control. In total, 66 spots for inhibition and 7 spots for activation were significantly altered. These spots were deduced from a total of 5,906 detected protein spots by applying the following criteria: [(a) protein spots matched >90% of all 2D-DIGE gels, (b) protein abundance changes >20% between treatment group (ADP, TRAP-6, CTAD or PGI_2_) and untreated control and (c) FDR-corrected ANOVA p-value < 0.05]. (1) Absolute numbers of separately and commonly altered protein spots upon inhibition (Σ = 66) and activation (Σ = 7). (2) Altered protein spots upon inhibition (Σ = 66) and activation (Σ = 7), that originated from distinct proteins: in sum, 31 proteins were identified (26-inhibition; 5-activation) of which 17 proteins were sensitive to phosphatase-treatment (15-inhibition; 2-activation) (3) The direction and magnitude of regulation is given for commonly and uniquely altered protein spots of each subgroup: CTAD = 45 (1 unique /44 common), PGI_2_ = 65 (21/44), ADP = 7 (1/6) and TRAP-6 = 6 (0/6). Relative fold changes [treated vs. untreated] are depicted and compared to the respective control (dashed line y = 1) on a logarithmic scale. (4) The absolute amount (|x|) of altered protein spots, regardless of direction, is represented as positive fold changes [treated vs. untreated] compared to the respective control with its corresponding medians. The median abundance difference was determined by a Mann-Whitney-U test with an α-level set at p < 0.05. *PGI*_2_ *- prostacyclin, CTAD - citrate, theophylline, adenosine, dipyridamole, ADP - adenosine-diphosphate, TRAP-6 – thrombin receptor-activating peptide-6*.

The proteome of these activated and inhibited platelets was compared with untreated controls to gain a deeper insight into phenotypic changes reflecting their activation status. Short-time acting PGI_2_ and the more stable, long lasting CTAD mixture were applied to inhibit platelets via the cAMP/PKA pathway. TRAP-6 and ADP were added for platelet activation. Platelet protein extraction was performed by TCA-precipitation of the gel-filtered platelet suspension to prevent any additional activation during pelleting by centrifugation. This precipitation step, however, prohibited the separation of the platelet secretome. Therefore, our experiment focused on events leading to changes within the non-secretory platelet proteome. To yield a high electrophoretic resolution quality these samples were separately investigated at the pH ranges of 4–7 and 6–9. Protein abundances were evaluated by DeCyder-image analysis software and quantified as fold changes compared to untreated controls. These analyses revealed 73 differentially regulated protein spots according to our statistical selection criteria. Upon PGI_2_ addition, 65 protein spots were changed, 45 were changed by adding CTAD, with a broad overlap (44 spots) (Fig. 2A). In contrast, six protein spots were regulated differently following TRAP-6 treatment compared to seven ADP-related protein spots (Fig. 2B). In summary, ten times more protein spots were affected by inhibition with a median abundance difference of 11% (+1.45- vs. +1.34-median FC, p = 0.03 Fig. 2A,B). Hence, in the non-secretory proteome inhibition footprints were qualitatively and quantitatively more prominent than activation ones, a finding that encouraged us to investigate the molecular characteristics for this difference.

### Platelet proteome changes after inhibition/activation are mainly related to phosphorylation

**Figure 3:**
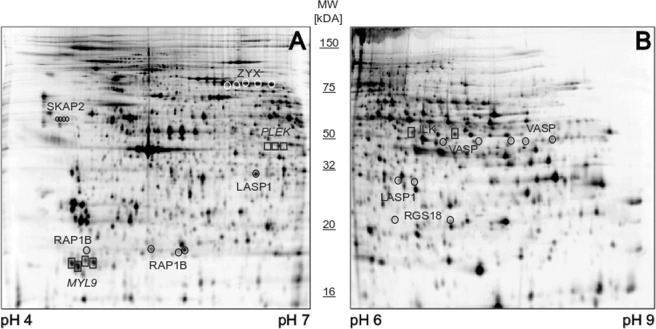
Representative 2D-DIGE gels pH 4–7 and pH 6–9 with significantly altered platelet proteins upon inhibition and activation are delineated. These exemplary monochrome-displayed 2D-DIGE images show significantly altered platelet protein spots upon inhibition and activation which are discussed in detail in the manuscript. In Supplementary Fig. S2 coloured 2D-DIGE images are displayed. A total of 36 µg (12 µg sample Cy3-, 12 µg sample Cy5-, and 12 µg internal standard Cy2-labeled) platelet protein extracts were separated according to the isoelectric point (pI) and the molecular weight (MW) in the pH ranges (**A**) pH 4–7 and (**B**) pH 6–9. Altered proteins were identified by mass spectrometry and are outlined for inhibition and for activation with their respective UniProt “Gene” name. Protein spots of interest were selected according to (a) protein spots matched >90% of all 2D-DIGE gels, (b) protein abundance changes >20% between treatment group and untreated control and (c) FDR-corrected ANOVA p-value <0.05.

**Figure 4:**
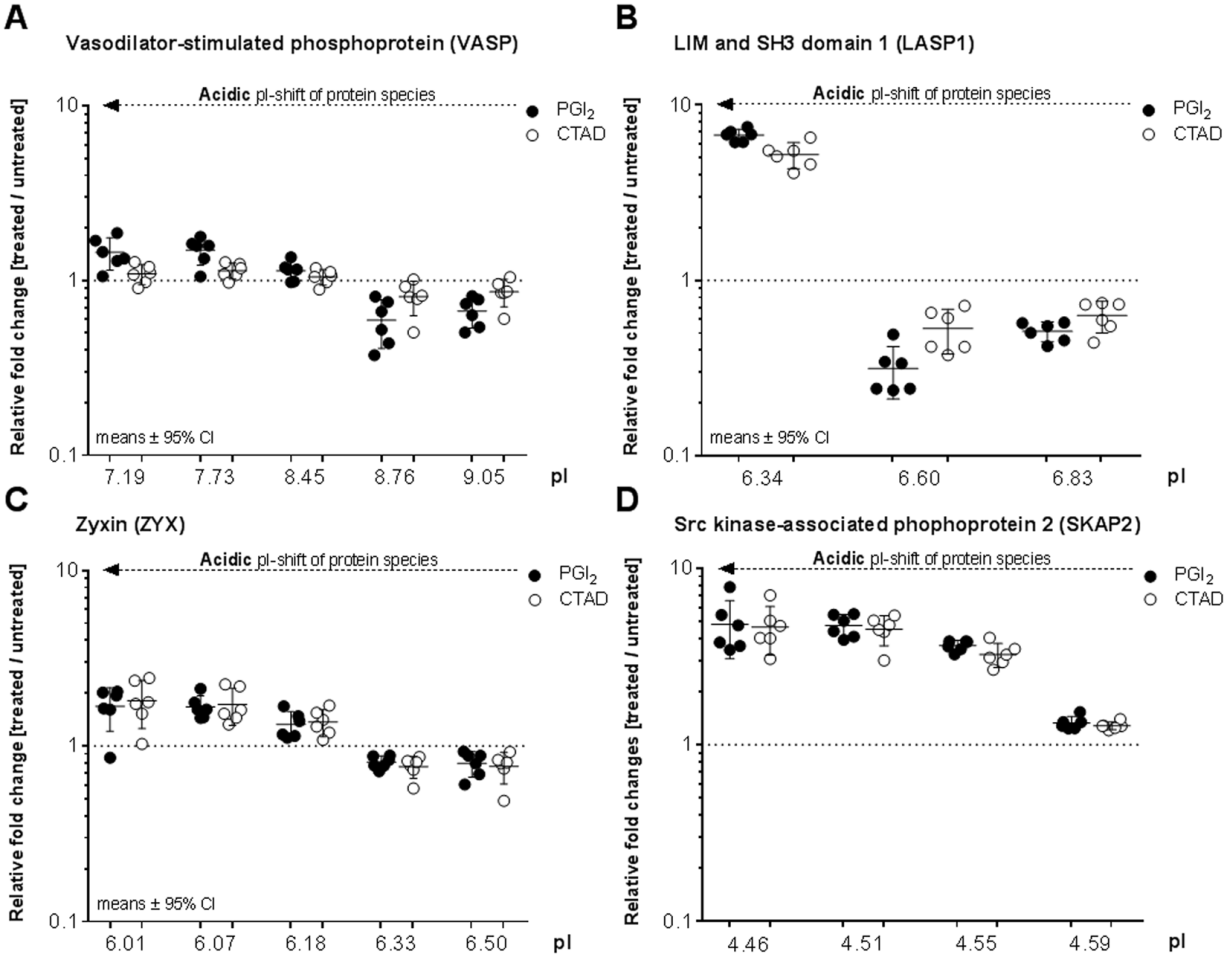
Platelet inhibition (by PGI_2_ or CTAD) induces shifts of protein spots to a more acidic pI. Significantly changed protein species, belonging to (**A**) VASP, (**B**) LASP1, (**C**) ZYX and (**D**) SKAP2 are depicted along their pI ranges. Values of each protein spots are given as relative fold changes between treated gel-filtered platelets (PGI_2_ ●, CTAD ○) and untreated control (dashed line y = 1) with their means and 95% confidence interval (n = 6 in each subgroup). Statistical significance was set at p < 0.05 and was determined by post-hoc testing between subgroups (PGI_2_, CTAD) and untreated controls. PGI_2_- and CTAD-dependent protein profiles of VASP were also validated by flow cytometry (Supplementary Fig. 11). *PGI*_2_ *- prostacyclin, CTAD - citrate, theophylline, adenosine, dipyridamole, pI - isoelectric point*.

To uncover the plethora of proteome changes seen in inhibited platelets compared to activated ones, the differentially regulated protein spots were identified by mass spectrometry. These results showed that several identified proteins appeared in more than one spot, giving a total of 31 distinct proteins (Tables 1 and 2, Fig. 2A2,B2). These proteoforms, originating from a single protein, had the same molecular weight, however, migrated across the pI range, thereby displaying a chain-like spot pattern (Fig. 3, Supplementary Fig. S4). For example, five different proteoforms with the same molecular weight but different pIs ranging from 7.19 to 9.05 were identified as VASP (Fig. 4A). A similar pattern was observed for LASP1, ZYX, and SKAP2 (Fig. 4B–D).

**Table 1:** Platelet proteins affected by inhibition (PGI_2_, CTAD)

Platelet Inhibition (n = 6)			Treated vs. untreated^*^		PGI_2_ vs. untreated^†^		CTAD vs. untreated^†^		λ-PPase^‡^	
UniProt - Gene	Protein ID	Isoelectric point (pI)	Average fold change (PGI_2_, CTAD/untreated)	cor. p-value (FDR < 0.05)	Fold change (PGI_2_/untreated)	cor. p-value (FDR < 0.05)	Fold change (CTAD/untreated)	cor. p-value (FDR < 0.05)	Fold change (PGI_2_ + λ − PPase/PGI_2_)	Fold change (CTAD + λ − PPase/CTAD)
ACTB	Actin, cytoplasmic 1	5.16	**1.30**	4–6E-03	**1.45**	2.5E-03	**1.15**	2.4E-01	1.10	0.84
		5.18	**1.28**	1.7E-02	**1.43**	9.5E-05	**1.12**	4.9E-01	1.08	0.78
ENO1	Alpha-enolase	6.70	**0.81**	5.9E-03	**0.79**	3.6E-03	**0.83**	1.3E-02	1.20	0.73
		6.60	**0.85**	9.3E-03	**0.81**	4.4E-03	**0.88**	5.4E-02	0.90	0.80
ACLY	ATP-citrate synthase	7.15	**0.82**	3.2E-03	**0.78**	8.1E-03	**0.88**	2.7E-02	1.68	1.34
CALD1	Caldesmon	6.15	**0.53**	4.0E-04	**0.54**	3.0E-04	**0.55**	2.5E-03	1.63	1.84
		6.24	**0.54**	7.0E-04	**0.54**	3.7E-04	**0.53**	2.8E-03	1.52	1.82
		6.41	**0.55**	3.4E-04	**0.57**	3.0E-04	**0.54**	4.4E-03	1.41	1.51
		6.47	**0.79**	2.2E-03	**0.81**	2.8E-03	**0.76**	6.6E-03	1.36	1.61
PRKG1	cGMP-dependent protein kinase 1	6.72	**0.78**	1.9E-03	**0.72**	9.9E-04	**0.81**	8.3E-03	2.06	2.21
CFL1	Cofilin-1	6.30	**0.70**	2.4E-03	**0.71**	1.1E-01	**0.68**	1.2E-03	0.12	0.24
DUSP3	Dual specificity protein phosphatase 3	7.10	**1.40**	7.4E-02	**1.45**	5.4E-03	**1.33**	1.1E-02	0.63	1.02
EIF4A1	Eukaryotic initiation factor 4A-I	5.44	**1.15**	1.4E-02	**1.26**	8.6E-03	**1.05**	6.1E-01	1.12	0.98
FGG	Fibrinogen gamma chain	5.39	**1.15**	1.6E-04	**1.25**	7.4E-05	**1.05**	9.1E-02	0.92	1.19
		5.49	**1.10**	8.7E-02	**1.22**	1.3E-02	**0.96**	4.1E-01	0.79	1.93
GSN	Gelsolin	5.77	**1.28**	5.9E-03	**1.46**	3.7E-03	**1.11**	2.1E-01	0.95	0.56
		6.10	**1.32**	9.6E-03	**1.31**	9.9E-03	**1.33**	8.4E-03	0.71	1.63
GAPDH	Glyceraldehyde-3-phosphate dehydrogenase	7.52	**1.38**	1.5E-02	**1.33**	2.6E-02	**1.43**	9.2E-03	1.05	0.75
SAR1A	GTP-binding protein SAR1a	7.20	**0.74**	4.6E-02	**0.74**	4.4E-03	**0.75**	2.7E-02	1.75	1.59
ILK	Integrin-linked protein kinase	6.74	**1.54**	5.9E-03	**1.56**	5.1E-02	**1.51**	5.7E-03	0.30	0.16
		7.10	**1.45**	4.1E-04	**1.43**	8.4E-03	**1.47**	3.7E-04	0.54	0.38
LASP1	LIM and SH3 domain protein 1	6.34	**5.97**	1.3E-09	**6.73**	1.3E-09	**5.22**	3.0E-09	0.15	0.21
		6.60	**0.42**	2.7E-04	**0.32**	2.0E-04	**0.53**	1.1E-02	3.13	2.34
		6.83	**0.57**	4.7E-03	**0.51**	3.0E-04	**0.63**	1.2E-03	1.70	1.69
LDHB	L-lactate dehydrogenase B chain	5.72	**1.18**	1.5E-02	**1.32**	1.1E-02	**1.04**	7.4E-01	0.92	1.31
MAPRE2	Microtubule-associated protein RP/EB	5.40	**1.35**	5.5E-03	**1.53**	3.0E-03	**1.17**	2.2E-01	0.68	0.70
MSN	Moesin	6.67	**1.43**	2.5E-03	**1.57**	3.6E-03	**1.29**	1.7E-02	1.13	0.99
		6.86	**0.66**	7.3E-04	**0.57**	9.8E-04	**0.75**	7.6E-03	2.88	2.56
		7.12	**1.28**	4.0E-04	**1.30**	3.0E-04	**1.26**	6.4E-04	0.95	0.79
NAP1L1	Nucleosome assembly protein 1-like 1	4.46	**0.81**	1.9E-03	**0.79**	1.4E-03	**0.82**	2.8E-03	1.01	0.95
		4.51	**0.33**	5.5E-07	**0.30**	4.0E-07	**0.35**	1.1E-06	0.81	0.70
		4.59	**0.39**	8.4E-06	**0.36**	8.7E-06	**0.42**	1.2E-03	5.96	6.29
PDLIM1	PDZ and LIM domain protein 1	6.38	**1.85**	5.9E-03	**1.93**	5.6E-03	**1.73**	5.0E-03	0.56	0.45
PLEK	Pleckstrin	6.47	**0.65**	9.7E-03	**0.64**	7.0E-03	**0.67**	1.3E-02	0.37	0.21
		6.60	**0.65**	5.7E-03	**0.63**	4.1E-03	**0.67**	8.0E-03	0.59	0.54
PPM1A	Protein phosphatase 1A	5.16	**1.32**	2.9E-04	**1.47**	1.1E-04	**1.16**	2.0E-02	0.75	0.94
		5.26	**1.19**	2.9E-04	**1.32**	1.6E-03	**1.05**	2.4E-01	1.03	1.25
RAP1B	Ras-related protein Rap-1b	4.75	**1.28**	5.9E-03	**1.45**	3.2E-03	**1.21**	7.0E-02	1.05	1.11
		5.40	**1.54**	1.8E-03	**1.38**	1.1E-02	**1.70**	8.4E-04	0.87	0.72
		5.61	**1.29**	2.5E-03	**1.27**	4.3E-03	**1.32**	1.9E-03	0.98	0.94
		5.64	**4.18**	8.4E-06	**3.56**	3.1E-05	**4.81**	2.4E-05	0.44	0.27
RGS18	Regulator of G-protein signaling 18	6.51	**0.71**	1.8E-03	**0.70**	1.5E-03	**0.71**	1.8E-03	0.54	0.51
		7.27	**1.32**	5.9E-03	**1.32**	6.2E-03	**1.31**	5.7E-03	0.56	0.66
SKAP2	Src kinase-associated phosphoprotein 2	4.46	**4.73**	2.4E-03	**4.82**	3.7E-04	**4.65**	1.4E-04	0.35	0.23
		4.51	**4.63**	2.6E-08	**4.74**	3.0E-08	**4.51**	3.2E-08	0.30	0.20
		4.55	**3.45**	1.6E-06	**3.65**	1.2E-06	**3.25**	7.6E-05	0.25	0.31
		4.59	**1.31**	9.4E-06	**1.33**	8.7E-06	**1.28**	2.4E-05	1.09	0.85
VASP	Vasodilator-stimulated phosphoprotein	7.19	**1.27**	7.8E-03	**1.45**	5.5E-03	**1.09**	2.4E-01	0.83	0.78
		7.73	**1.32**	1.2E-03	**1.50**	5.9E-04	**1.14**	8.6E-02	0.37	0.41
		8.45	**1.09**	4.6E-02	**1.13**	2.6E-02	**1.05**	9.1E-01	0.68	0.73
		8.76	**0.70**	3.2E-03	**0.60**	1.7E-03	**0.81**	7.3E-02	2.05	1.13
		9.05	**0.77**	2.2E-03	**0.67**	1.1E-03	**0.86**	7.2E-02	1.93	1.08
ZYX	Zyxin	6.01	**1.75**	3.3E-03	**1.69**	4.9E-03	**1.81**	2.7E-03	0.41	0.36
		6.07	**1.70**	4.5E-04	**1.68**	6.4E-04	**1.72**	4.4E-04	0.57	0.89
		6.18	**1.35**	2.3E-03	**1.33**	3.3E-03	**1.37**	1.9E-03	0.85	0.93
		6.33	**0.82**	1.5E-02	**0.84**	2.1E-02	**0.79**	5.7E-02	1.23	1.32
		6.50	**0.78**	1.6E-02	**0.80**	2.3E-02	**0.76**	1.0E-02	1.65	1.79

^*^Mixed ANOVA (PGI_2_ vs. CTAD vs. untreated).

^†^Alterations within subgroups by post-hoc contrasts (PGI_2_, CTAD vs untreated).

^‡^λ-PPase-treated (λ-PPase + PGI_2_, λ-PPase + CTAD) and λ-PPase-untreated gel-filtered platelets (PGI_2_, CTAD).

The tables summarise alphabetically (*Protein-ID*) altered 2D-DIGE protein spots with their corresponding protein abundance changes upon inhibition (PGI_2_/CTAD). Alterations are given as average fold changes between subgroups (PGI_2_, CTAD) and their respective untreated control (baseline). P-values corrected for multiple comparisons were obtained by post-hoc testing between the respective treatment subgroup and untreated controls. The sample size of each subgroup was n = 6. Proteins of interest were defined as (a) matched in 90% of all gels and (b) average fold change >20% between at least one treatment condition and untreated (PGI_2_ and/or CTAD vs. untreated) and c) a FDR-corrected p-value <0.05 obtained by a mixed ANOVA (PGI_2_ vs. CTAD vs. untreated). Fold changes between λ-PPase-treated (λ-PPase + PGI_2_, λ-PPase + CTAD) and -untreated (PGI_2_, CTAD) samples are given to outline the protein phosphorylation status (n = 1). Fold changes >20% between λ-PPase-treated and –untreated samples are considered as relevantly phosphorylated.

PGI_2_ - prostacyclin, CTAD - citrate, theophylline, adenosine, dipyridamole, ADP - adenosine-diphosphate, TRAP-6 – thrombin receptor-activating peptide-6, λ-PPase - lambda-phosphatase.

**Figure 5:**
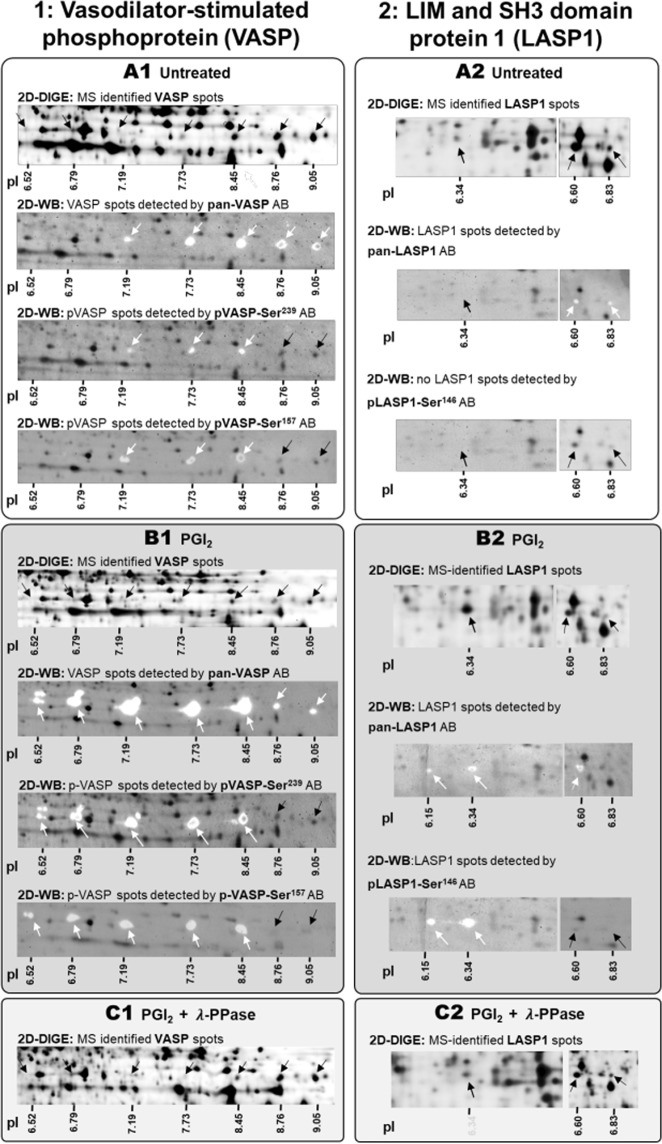
Detection of phosphorylated platelet protein species of VASP and LASP1 using phospho-site specific antibodies and λ-phosphatase treatment. Proteoform abundances of (**1**) VASP (left panel) and (**2**) LASP1 (right panel) are shown in (**A**) untreated control, (**B**) PGI_2_- treated, and (**C**) PGI_2_ + λ phosphatase (λ-PPase) treated platelets (**A–C**) detectable by two-dimensional gel electrophoresis in the pH range 4–7 and 6–9 along their isolelectric point (pI). The arrows indicate the respective protein spot and respective pI´s are subscribed. The same protein preparations as analysed by 2D-DIGE were used for 2D-WBs and the applied protein amount was 36 µg. Images (**A–C**) constitute 2D-DIGE and 2D-WB images from one platelet sample. The gel-sections were obtained from scanned 2D-DIGE gels or 2D-WBs and represent the respective protein area. (1) VASP and (2) LASP1 proteoforms obtained following CyDye-labelled 2D-DIGE and 2D-WB are indicated as black spots and arrows. VASP and LASP1 proteoforms detected via specific antibodies and visualised following HRP generated chemoluminescence signals are shown as white spots and indicated with white arrows. Antibody signals were overlaid with the corresponding CyDye-labeled two-dimensional separated platelet proteome to evaluate which protein spots are recognised by the pan as well as phospho-site specific antibodies. (**A**) Platelet protein extracts from citrate anticoagulated blood constitute the untreated control. (**B**) PGI_2_-treatment shifts and increased protein spot abundances towards the acidic pH-range with a concomitant reduction of the corresponding alkaline spot abundances. (**C**) Λ-PPase treatment removes the phosphorylation of respective protein species thereby shifting its pI towards the alkaline direction. The LASP1 images (2) overlap (vertical solid line) between pH 6–7 due to its wide-spreading across the pI-range (pH 6–7), resprective non-cropped 2D images are displayed in Supplementary Fig. S7. *VASP - Vasodilator-stimulated phosphoprotein, LASP1 - LIM and SH3 domain protein 1, λ-PPase -lambda phosphatase, pI - isoelectric point*.

Subsequently, biological database analysis (Reactome) was applied to identify the most probable underlying biological processes. Knowledge-based Ingenuity Pathway Analysis alongside literature research revealed that one key node of this network was PKA upon inactivation. Since almost all proteins with the strongest changes in abundance (>1.5-fold; LASP1, SKAP2, NAP1L1, RAP1B, CALD1, ZYX, and VASP) belonged to the cAMP/PKA-pathway (Supplementary Fig. S5), an involvement of post-translational modifications such as phosphorylation was anticipated. To provide biochemical evidence, protein lysates of PGI_2_ and CTAD-treated platelets were treated with λ-phosphatase (λ-PPase)^31^. Phosphorylated proteins lose their phospho-groups after λ-PPase treatment (treated + PPase/treated < 0.8), thereby causing a pI-shift towards an alkaline pH accompanied by an increase in the protein abundance of the corresponding “native” protein spots (treated + PPase/treated > 1.2). Using this approach, we were able to identify phosphorylation of protein species due to pI-dependent abundance changes (Table 1). Following the addition of λ-PPase, phosphorylation patterns were confirmed for VASP (pI 7.19–9.05; Fig. 5C1), LASP1 (pI 6.34–6.83; Fig. 5C2), alongside other potential PKA substrates such as RAP1B and ZYX (Supplementary Fig. S6).

Protein spot identities and phosphorylation profiles of VASP and LASP1 were further characterised using pan and phosphosite-specific antibodies by 2D Western blots. The pan VASP antibody detected spots at five pI positions in the untreated control (Fig. 5A1) and at seven pI positions in the Western blot from PGI_2_-treated platelets (Fig. 5B1). Thus, all MS identifications were verified and additional PGI_2_-inducible VASP spots located at pI 6.52 and 6.79, were immunologically identified. Moreover, the PGI_2_ induced VASP protein profile displayed several spots with molecular weight shifts at the particular pIs between 6.52 and 8.45. The pVASP-Ser^239^ as well as pVASP-Ser^157^ antibody recognised pre-phosphorylated VASP spots at the pIs 7.19, 7.73 and 8.45 in the control proteome and detected additional phosphorylated protein species, visible by more acidic spots at the pIs 6.52 and 6.97 in the PGI_2_ treated samples (Fig. 5B1; white arrows). The two VASP spots with the most akaline pIs (8.76 and 9.05) were not detected by any of the phosphosite-specific VASP antibodies (Fig. 5B1; black arrows), which confirmed their unphosphorylated state as already indicated by 2D-DIGE analysis. These spots displayed a decreased abundance after PGI_2_ treatment and an increased abundance after λ-PPase treatment (Table 1). A molecular shift from 46 kDa to 50 kDa is described for VASP after phosphorylation at Ser157^32^. Thus, the pVASP-Ser^157^ antibody should only recognize VASP signals at the molecular weight of 50 kDa. To proof these observations using the current samples, 2D Western blots were repeated with 12.5% SDS-PAGE in the second dimension to achieve a better resolution at this MW region (Supplementary Fig. S8). These 2D Western blots clearly showed separated pVASP-Ser^157^ antibody spot signals only at 50 kDa with different pIs (Supplementary Fig. S8**)** indicating a sequence and different combinations of multi-site phosphorylation events. A hypothesis for VASP multisite phosphorylation chronology is delineated in Supplementary Fig. S9.

The pan LASP1 antibody recognised two spots in the control (pI 6.60 and 6.83; Fig. 5A2) and two additional, more acidic spots after PGI_2_ treatment (pI 6.15 and 6.34) (Fig. 5B2). These two spots, based on PGI_2_ (and CTAD) induced PKA phosphorylation were verified by the phosphosite-specific antibody pLASP-Ser^146^ (Fig. 5B2). No signals were detetced in the controls by pLASP-Ser^146^ immunostaining (Fig. 5A2). Phosphorylation profiles of RAP1B were also validated with a panRAB1B antibody and 1D Western blot (Supplementary Fig. S10).

Platelet activation by ADP and TRAP-6 induced significant abundance changes in MYL9, ILK, and PLEK. A pathway analysis allocated these proteins to the phospholipase C/protein kinase C (PKC-) pathway (Supplementary Fig. S5*)*. Thus, potential PKC-mediated phosphorylation of these spots could also be identified by λ-PPase treatment (Table 2).

**Table 2:** Platelet proteins affected by activation (ADP, TRAP-6).

Platelet Activation (n = 6)			Treated vs. untreated^*^		ADP vs. untreated^†^		TRAP-6 vs. untreated^†^		λ-PPase^‡^	
UniProt - Gene	Protein ID	Isoelectric point	Average fold change (ADP, TRAP-6/untreated	cor. p-value (FDR < 0.05)	Fold change (ADP/untreated	cor. p-value (FDR < 0.05)	Fold change (TRAP-6/untreated)	cor. p-value (FDR < 0.05)	Fold change (PGI_2_ + λ − PPase/PGI_2_)	Fold change (CTAD + λ − PPase/CTAD)
UQCRC2	Cytochrome b-c1 complex subunit 2	7.81	**0.82**	1.4E-04	**0.85**	7.9E-04	**0.79**	7.9E-03	1.24	1.19
ILK	Integrin-linked protein kinase	7.10	**0.80**	1.4E-04	**0.72**	1.7E-04	**0.89**	2.7E-02	0.30	0.16
IDH2	Isocitrate dehydrogenase	8.37	**0.74**	1.4E-04	**0.74**	2.5E-04	**0.74**	1.9E-04	1.11	1.05
MYL9	Myosin regulatory light polypeptide 9	4.74	**1.31**	3.6E-04	**1.23**	6.6E-03	**1.39**	6.6E-03	1.15	0.84
PLEK	Pleckstrin	6.52	**1.65**	1.4E-04	**1.76**	7.9E-04	**1.53**	5.4E-04	0.32	0.21
		6.60	**1.60**	1.4E-04	**1.62**	1.7E-04	**1.58**	1.7E-04	0.59	0.54

^*^Mixed ANOVA (ADP vs. TRAP-6 vs. untreated).

^†^Alterations within subgroups by post-hoc contrasts (ADP, TRAP-6 vs. untreaed).

^‡^λ-PPase-treated (λ-PPase + PGI_2_, λ-PPase + CTAD) and λ-PPase-untreated gel-filtered platelets (PGI_2_, CTAD).

The tables summarise alphabetically (*Protein-ID*) altered 2D-DIGE protein spots with their corresponding protein abundance changes upon activation (ADP/TRAP-6). Alterations are given as average fold changes between subgroups (ADP, TRAP-6) and their respective untreated control (baseline). P-values corrected for multiple comparisons were obtained by post-hoc testing between the respective treatment subgroup and untreated controls. The sample size of each subgroup was n = 6. Proteins of interest were defined as (a) matched in 90% of all gels and (b) average fold change >20% between at least one treatment condition and untreated (ADP and/or TRAP-6 vs. untreated) and c) a FDR-corrected p-value < 0.05 obtained by a mixed ANOVA (ADP vs. TRAP-6 vs. untreated). Fold changes between λ-PPase-treated (λ-PPase + PGI_2_, λ-PPase + CTAD) and -untreated (PGI_2_, CTAD) samples are given to outline the protein phosphorylation status (n = 1). Fold changes >20% between λ-PPase-treated and –untreated samples are considered as relevantly phosphorylated.

In conclusion, these experiments confirm that phosphorylation is a predominant proteomic feature in defining platelet activation status and especially their inhibition. Beyond that, these findings propose more powerful protein biomarker candidates compared to the established platelet reactivity marker pVASP.

### 2D-DIGE analysis of inhibited platelets reveals stronger phosphorylated PKA targets than pVASP

**Figure 6:**
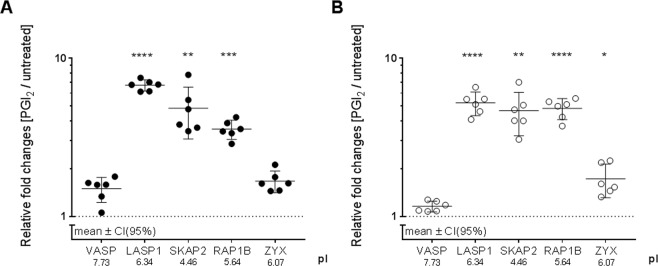
Platelet inhibition causes stronger phosphorylation changes in LASP1, SKAP2 and RAP1B than in VASP. The acidic protein spot abundances of VASP, LASP1, SKAP2, RAP1B, and ZYX are depicted as fold changes between treated gel-filtered platelets after (**A**) PGI_2_ ● and (**B**) CTAD ○ treatment with their means, 95% confidence interval (CI) and isoelectric point (n = 6 in every group). Statistical significance was set at p < 0.05 and determined by a repeated ANOVA with single post-hoc contrast compared to VASP. Statistical significance is marked and represents the respective acidic protein abundance compared to VASP abundance. *p < 0.05 **p < 0.01 ***p < 0.001 ****p < 0.0001.

To evaluate the phosphorylation degree, protein abundances of phosphorylation-sensitive protein spots were compared between PGI_2_/CTAD treated and untreated platelets. Notably, 15 out of 26 inhibition-affected proteins originated from two or more protein spots, only differing in their pI. Λ-PPase treatment and use of phosphosite-specific antibodies revealed that phosphorylation is responsible for this phenomenon. VASP, the most frequently described phosphorylation target of PKA/PKG, was affected in its phosphorylation profile by PGI_2_ treatment (+1.45-FC at pI 7.19; p = 5.5E-03 and +1.50-FC at pI 7.73; p = 5.9E-04). CTAD treatment, on the other hand, did not significantly change the abundance of phosphorylated VASP protein species (+1.09-FC at pI 7.19; p = 2.4E-01 and +1.14-FC at pI 7.73; p = 8.6E-02) (Table 1, Fig. 4 and 6). These findings were also confirmed by 1D Western blot (Supplementary Fig. S11) and flow cytometry (Supplementary Fig. S12), which indicate that especially pVASP-Ser^157^ are weaker phosphorylated by CTAD compared to PGI_2_ treatment. Interestingly, most of the other potential PKA-targets showed stronger abundance changes in their phosphorylated protein species than VASP. Moreover, this stronger degree of phosphorylation was induced by PGI_2_- as well as by CTAD-treatment, like pLASP1-Ser^146^ (pI 6.34, +6.73-FC after PGI_2_ and +5.22-FC after CTAD), SKAP2 (pI 4.46, +4.82-FC and +4.65-FC), RAP1B (pI 5.64, +3.56-FC and +4.81-FC), and ZYX (pI 6.07, +1.68-FC and +1.72-FC) (Table 1, Fig. 4 and 6). For pLASP1-Ser^146^ (pI 6.34), RAP1B (pI 5.64) and SKAP2 (pI 4.46), these changes were still present additional 25 minutes after PGI_2_ addition to platelet rich plasma and gel-filtration. (Supplementary Fig. S13 and S1*)*. Likewise, these quantitative differential phosphorylation profiles were also apparent at the expense of the corresponding unphosphorylated proteoforms. For example, the FC of unphosphorylated VASP spot at pI 8.76 was 0.60 after PGI_2_ treatment while LASP1 (pI 6.60) exhibited an FC of 0.32 (Table 1, Fig. 4A,B). Thus, we demonstrated that platelet inhibition by PGI_2_ or CTAD induced stronger phosphorylation profiles in LASP1, RAP1B, ZYX and SKAP2 species than in VASP. Additionally, the findings of differential phosphorylation degree of RAP1B, VASP and LASP1 were also confirmed and visualised by 1D Western blots analysis (Supplementary Fig. S10C and S11A).

Finally, we were also interested in identifying platelet protein markers for activation and comparing them to the established activation marker CD62P.

### The platelet activation marker CD62P is strongly associated with phosphorylated species of PLEK and ILK

**Figure 7:**
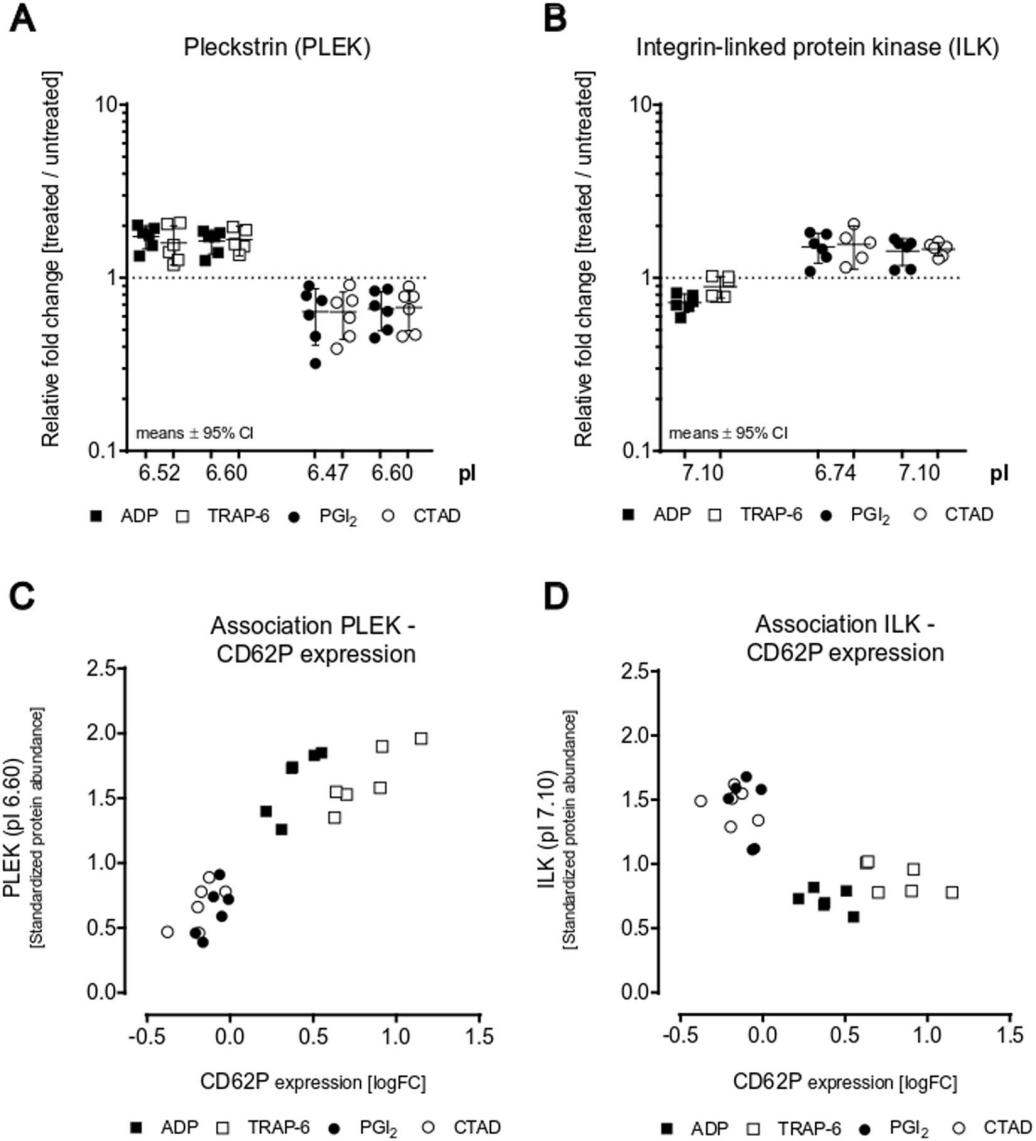
Both platelet inhibition and activation regulate phosphorylated PLEK and ILK protein species. The protein abundance changes of (**A**) PLEK and (**B**) ILK are depicted as fold changes between treated gel-filtered platelets (ADP □, TRAP-6 ■, PGI_2_ ● or CTAD ○) and untreated control (dashed line y = 1) with their means and 95% confidence interval (CI). Since (**C**) PLEK (pI 6.60), (**D**) ILK (pI 7.10) and platelet CD62P expression are affected by both activation and inhibition, the association between protein levels and functional parameters was assessed showing a scatter dot plot of the relation between protein levels [relative fold changes] and the logarithmic platelet CD62P surface expression [relative fold change]. n = 6 (PGI_2_, CTAD), n = 6 (ADP, TRAP-6). *PGI*_2_ *- prostacyclin, CTAD - citrate, theophylline, adenosine, dipyridamole, ADP - adenosine-diphosphate, TRAP-6 – thrombin receptor-activating peptide-6, pI - isoelectric point, logFC - logarithmic fold change*.

After ADP or TRAP-6-mediated platelet activation, a considerably lower number of seven significantly changed protein spots were detected in the non-secretory proteome compared to platelet inhibition (Fig. 2). However, PLEK, the major substrate of PKC, was found to be significantly upregulated with two different protein spots (pI 6.52 and pI 6.60) and λ-PPase treatment confirmed this phosphorylation (Table 2). Interestingly, ILK, as well as PLEK, were the only two proteins that were affected by both, activation and inhibition of platelets. PLEK phosphorylation at pI 6.60 significantly increased upon activation and in addition, showed a reduced spot signal upon inhibition when compared to the untreated controls (Table 2, Fig. 7A). Inversely, ILK at pI 7.10 was dephosphorylated upon activation and phosphorylated after inhibition (Fig. 7B). To assess whether the phosphorylation profiles of PLEK and ILK might be related to the so far used platelet activation marker CD62P, we evaluated its association. Such a correlation could be confirmed as illustrated in Fig. 7C,D. Moreover, despite the potent effect size of 2.35 Cohen’s d for CD62P, the quantitative discrimination power between activation and inhibition was even more pronounced for the phosphorylated PLEK (3.77 Cohen´s d; pI 6.60) and ILK (3.79 Cohen’s d; pI 7.10) protein spots. These results indicate that phosphorylated ILK and PLEK species can distinguish more effectively between activation and inhibition when compared to the commonly used platelet activation marker CD62P.

## Discussion

Increased platelet reactivity may result in thrombosis and other vascular diseases. By investigating the platelet proteome after *in-vitro* activation and inhibition, we attempted to uncover discriminative signs for these modulations in platelet activation state. Here, we expose for the first time that platelet inhibition was associated with more pronounced alterations in the non-secretory platelet proteome as compared to activation. Moreover, we showed that these changes were mainly caused by phosphorylation, suggesting a predominant role of this post-translational modification in maintaining platelets in their resting state. Novel diagnostic options were revealed, since PGI_2_ or CTAD inhibited platelets showed stronger phosphorylation changes in PKA targets than the benchmark platelet reactivity marker pVASP. Additionally, phosphorylated species of PLEK and ILK were associated with CD62P surface expression and showed an even larger effect size than this established activation marker.

In our representative 2D-DIGE platelet proteome analysis, comprising 5,906 protein spots, 66 and 7 protein spots were significantly changed in response to 15 minutes inhibition and activation stimuli, respectively. However, changes caused by platelet protein secretion or protein dynamics in the early phase of inhibition, as previously shown^17^, are not captured based on our study design. On a physiological basis, the disparity between inhibition and activation induced changes in the proteome could be due to platelets being more responsive to inhibitory stimuli. Potent platelet inhibition is constantly needed in vascular system due to the exposure to shear stress, acting as a strong platelet activator^33^. Shear stress is permanently present under physiological conditions and is much more pronounced in pathophysiological conditions, e.g. carotid or coronary vessel stenosis^34,35^. Accordingly, the present findings demonstrate that the addition of PGI_2_ or CTAD, to obtain resting platelets, strongly influences the non-secretory platelet proteome. Thus, after inhibition, 15 out of 26 proteins were altered in their phosphorylation levels. Knowledge-based pathway analysis demonstrated that the unbiased 2D-DIGE proteomics technology specifically accentuated the cAMP-induced PKA-signaling pathway in PGI_2_ or CTAD-treated platelets. Finally, we characterised multiple proteoforms at seven pI positions of the well-known PKA phosphorylation target VASP – a negative regulator of platelet activation^36^ and a clinically established platelet reactivity marker^28,37,38^. Remarkably, CTAD-treated platelets did not show a significant phosphorylation in any 2D-DIGE detected VASP species. Intriguingly, other spots showed substantially stronger abundance changes in their phosphorylation levels than phosphorylated VASP species: LASP1 6.7-fold, RAP1B 4.1-fold, SKAP2 4.6-fold, ZYX 1.7-fold, and RGS18 1.5-fold compared to VASP with 1.3-fold. Previously, a 6.7-fold stronger phosphorylation of RAP1B compared to VASP was reported in platelets treated with cAMP-elevating reagents^39^, which supports the reliability of our data. This latter study showed that VASP phosphorylation was reversible with maximum phosphorylation after 10 minutes, dropping to baseline after 60 minutes. Conversely, RAP1B phosphorylation was irreversible with constant levels over 2 hours^39^. Beyond RAP1B, the stability of PGI_2_- and CTAD-induced changes in LASP1 and SKAP2 phosphorylation was confirmed for at least 40 minutes in our study (Supplementary Fig. S13). This long-lasting stability of these phosphorylation events is of additional interest, since it is generally accepted that after about 25 minutes at room temperature the biologically active PGI_2_ is degraded and the inhibitory influence of PGI_2_ comes to an end.

The phosphorylated protein species of LASP1, RAP1B and RGS18^40^ have already been described as targets for PKA in the inhibitory pathways of platelets^41,42^. For ZYX, phosphorylation after stimulation of the cAMP/PKA pathway by forskolin has recently been shown in endothelial cells^43^. Until now, SKAP2 is not shown as a target of PKA, but its phosphorylation upon iloprost and PGI_2_ treatment in resting platelets has been reported^17,44^. These actual novel findings in cAMP-mediated quantitative differential phosphorylation degree of PKA targets suggest that e.g. phosphorylated LASP1, RAP1B, and SKAP2 could be technically more robust biomarkers to measure the platelet activation status than phosphorylated VASP. A previous proteome analysis of inactivated platelets employing gel-free MS analysis from TiO_2_-enriched phospho-peptides revealed 300 proteins with differential phosphorylation patterns between 10 to 60 seconds after stimulation with the stable PGI_2_ analogue iloprost^17^. However, a stronger upregulation in phosphorylation of LASP1, ZYX, SKAP2, RGS18, and RAP1B in comparison to a weaker phosphorylation of VASP was not reported. The authors just stated that pVASP-Ser^239^ had the highest degree of phosphorylation with a 7.1-fold up-regulation after 60 s platelet inhibition in the presence of 5 nM Iloprost^17^. The PKA phosphorylation targets pLASP1-Ser^146^ and pSKAP2-Ser^283^ or Ser^286^ were found to be less phosphorylated (both 3.6-fold upregulation) in comparison to phosphorylated pVASP-Ser^239^. Phosphorylated peptides from RAP1B were not detected and RGS18 phosphorylation decreased. However, these discrepancies regarding the phosphorylation degree of these PKA targets may be related to their earlier measuring points (10, 30 and 60 s) in addition to the completely different methodology employed. Compared to the number of 26 inhibition-affected platelet proteins detected in our study, a considerably higher number with 300 altered phosphorylated proteins from iloprost-inactivated platelets was described in the study by Beck *et al*.^17^. This higher count of identified potential PKA targets was achieved since the used TiO_2_ affinity-based method enriches phospho-peptides and enables the detection of phosphorylated low-abundant proteins^17^. With this enrichment step, however, a direct quantitative assessment of phosphorylation events compared to the global platelet proteome is lost^17^. Our 2D-DIGE snapshot, on the other hand, displayed the relationship of protein phosphorylation to the overall proteome and, thereby also highlights the specificity of platelet inhibition pathways.

Despite a prominent CD62P surface upregulation, the actual 2D-DIGE based analysis revealed unexpected weaker changes in the non-secretory proteome of activated platelets compared to inhibited platelets. In contrast to previous studies investigating the activated platelet proteome by two-dimensional gel electrophoresis^13,24,45,46^, our experimental design could only measure non-secretory proteome changes but cannot dissect alterations based on granula and microvesicle release. However, our statistical stringent platelet proteome analysis by Benjamini-Hochberg correction showed in total only seven protein spots changed after platelet activation. Without correction for multiple comparisons (FDR), we would have detected 32 additional proteins upon ADP- and TRAP-6 activation. Nevertheless, these changes in spot counts are quite comparable to the explorative 2D-DIGE based proteomics study of gel-filtrated platelets from Schulz *et al*. with 13 differentially regulated protein spots after activating the glycoprotein VI receptor^47^. In this study, the degree of platelet stimulation was also quantified by CD62P upregulation^47^ and is comparable to our *in-vitro* platelet activation CD62P profiles. Evidence for the specific stimulation of platelet activation pathways in our study is the phosphorylation of well-known PKC substrates such as PLEK and MYL9, proteins that were also detected in most of the published 2D gel-based proteomics studies of *in-vitro* activated platelets^13,24,47,48^. Recently, Beck *et al*.^19^ characterized 302 phosphorylated proteins following ADP-treatment with kinetics between 10- and 60 seconds. Despite the high sensitivity of TiO_2_ enrichment, some important PKC substrates in platelet activation, such as MYL9, have not been detected^19^. These differences in results indicate that each technical proteomic approach has distinct strengths and limitations. For the present proteomics study by 2D-DIGE analysis the following limitations need to be considered: under-representation of low abundant proteins, very hydrophobic proteins and proteins with a high molecular weight. However, 2D-DIGE analysis also provides several advantages like highly reliable qualitative and quantitative accurate profiling of differentially abundant proteins by the inclusion of an internal standard which enables a low technical variation with a CV of 7%^29^. Finally, only protein-based “top-down” proteomics as 2D-DIGE can provide reliable quantitative results from different proteoforms^49^. For LC-MS/MS mass spectrometry-based proteome analysis tryptic digestion of the protein samples is necessary but causes a loss of information for the correct assignment of peptides and post-translational modifications to the appropriate proteoform^49^.

In the present study, PLEK (pI 6.60) and ILK (pI 7.10) species were the only two proteins that were altered by both platelet activation and inhibition. Apart from that, a pronounced overlap in the affected proteome was not found. This seems plausible since ADP and thrombin inhibit adenylyl cyclase activity via G_i_, the central enzyme in the inhibitory cAMP/PKA pathway^50^. PKC-dependent phosphorylation of PLEK is a sensitive marker for platelet activation^51^. Although ILK is described as a substrate for PKC and PI3K^52^ to induce platelet adhesion^53^, we found an explicit dephosphorylation pattern for ILK (pI 7.10) in activated platelets that has not been reported before. Despite a potent effect size of 2.35 (Cohen’s d) for the established platelet activation marker CD62P, the quantitative discrimination power between activation and inhibition was even more pronounced for phosphorylated PLEK (3.77 Cohen’s d; pI 6.60) and ILK (3.79 Cohen’s d, pI 7.10) species. As platelet CD62P expression may decrease in severe clinical states of platelet activation^54^, potentially due to receptor- and microvesicle-shedding and/or platelet adhesion to endothelial cells and leukocytes, phosphorylated ILK and PLEK species may represent further more robust diagnostic targets for platelet activation.

These stronger phosphorylation changes of the non-secretory proteome from inhibited compared to activated platelets may provide fundamental new insights into quantitative functional regulations of platelets. From there, new and more robust biomarkers for platelet inhibition and activation as well as new therapeutic options could follow, which may enable to treat abnormal platelet function more targeted in accordance with underlying pathophysiological mechanisms.

## Methods

### Platelet preparation and *in-vitro* conditions for platelet inhibition and activation

The study included twelve healthy volunteers aged 31.7 ± 7.1 years (mean ± SD), who had not taken any antiplatelet medication for at least 14 days prior to blood sampling. Individuals had a 1:1 gender ratio and were all normal weight (BMI 23.6) non-smokers. The study was approved by the Ethics Committee of the Medical University of Vienna (1076/2016). Each participant was advised of the purpose of the study and written informed consent was obtained prior in accordance with the principles of the Declaration of Helsinki. Blood was drawn from the antecubital vein (21 G needle) without stasis into sodium-citrate tubes (0.129 mM citrate) as well as CTAD tubes (0.129 mM trisodium citrate, 15 mM theophylline, 3.7 mM adenosine, 0.198 mM dipyridamole), a mixture of cAMP-elevating agents. These commercially available CTAD blood tubes are used to prevent platelet activation during blood sampling and subsequent processing steps^51^. Blood tubes were centrifuged at 120 xg for 20 minutes to obtain platelet-rich plasma (PRP). A graphical experimental design for *in-vitro* treatments is displayed in the Supplementary Fig. S1. Briefly, for inhibition, PRP was incubated for 5 minutes with PGI_2_ (0.4 µM final at RT) before gel-filtration, while PRP from CTAD blood tubes was left untreated. A PGI_2_ concentration of 0.1–0.5 μM is normally used to generate washed platelets^21,23,24^. To gently purify platelets from plasma proteins by size-exclusion chromatography^25^, 1 ml PRP was applied onto a 12 cm sepharose-2B packed column pre-equilibrated and eluted in PBS buffer. Gel-filtered platelets were caught at the end of the column and precipitated by mixing 3:1 (v/v) with 6.1 N TCA solution (containing 80 mM DTT). Thus, the inhibition of platelets by PGI_2_ was stopped after altogether 15 minutes and by CTAD after 40 minutes. Alternatively, PGI_2_ was added not to the PRP, but to the gel-filtered platelet suspension, which induced comparable changes in the proteome as seen in PRP + PGI_2_ (Supplementary Fig. S2). For activation, gel-filtered platelets derived from PRP were incubated either with ADP (5 µM) or TRAP-6 (15 µM) as described previously^20,27^ to evoke a moderate or strong activation response, respectively. Gel-filtration constitutes a gentle method to isolate platelets^52^, however, activated platelets cannot pass the sepharose column and get stuck, resulting in a diminished platelet yield (Supplementary Fig. S3). Therefore, we decided to activate platelets after gel-filtration. Stimulations were stopped by TCA/DTT precipitation after 15 minutes at RT. For baseline values, corresponding citrated PRP was gel-filtered and immediately precipitated. After one hour at 4 °C, the TCA-extracted platelet proteins from all conditions were pelleted at 20,000 × g for 10 minutes at 4 °C and washed four times (20,000 × g, 10 minutes, 4 °C) with acetone containing 20 mM DTT.

### Platelet proteome analysis by fluorescence two-dimensional gel electrophoresis (2D-DIGE)

Resolubilisation of precipitated proteins, determination of protein concentration, preparation of the internal standard and labelling with fluorescent cyanine dyes (Cy2, Cy3, Cy5) were performed as described previously^29^. Briefly, the TCA-precipitated and acetone-washed platelet proteins were re-solubilised in urea-sample buffer (7 M urea, 2 M thiourea, 4% CHAPS, 20 mM Tris-HCl pH 8.5) and fluorescence-labeled prior to 2D-separation. For the normalisation process, an internal standard (IS) of platelet proteins from all treatment conditions was prepared. This IS, labelled in Cy2 and added to every 2D-gel, enables a highly accurate qualitative and quantitative comparison of all samples and treatments^28,53^. The 2D-DIGE analysis was performed in the pH-ranges 4–7 and 6–9, respectively. For the acidic side, 36 μg protein (2 × 12 μg sample +1 × 12 μg internal standard) were passively rehydrated on 24 cm pH 4–7 IPG-Dry-Strips in a solution containing 7 M urea, 2 M thiourea, 4% CHAPS, 70 mM DTT and 0.5% ampholyte pH 4–7. For the alkaline side, 24 cm pH 6–9 IPG-Dry-Strips (GE Healthcare, Uppsala, Sweden) were soaked in a rehydration solution containing 7 M urea, 2 M thiourea, 4% CHAPS, 150 mM DTT and 2% ampholytes pH 6–9 prior to isoelectric focusing. Afterward, 3 × 12 μg of labeled protein samples were applied by “cup-loading” via the acidic side and the isoelectric focusing was carried out until 30 kVh was reached. The following SDS-PAGE was performed with an 11.5% acrylamide gel at 35 V for 1 hour, 50 V for 1.5 hours and 110 V for 16.5 hours at 10 °C in an Ettan DALTsix electrophoresis chamber (GE Healthcare, Uppsala, Sweden). The gels were scanned afterward with a resolution of 100 μm using a Typhoon 9410 imager (GE Healthcare, Uppsala, Sweden). The experimental study design of the 2D-DIGE analysis is delineated in Table S1.

### Identification of protein spots by mass spectrometry

For MS-based identifications, 250 µg unlabeled proteins were separated on a preparative two-dimensional gel. Proteins were visualised by MS-compatible silver staining^8^. Spots differentially regulated on the basis of 2D-DIGE analysis were excised with a scalpel, destained, reduced, alkylated and tryptically digested. Peptides were applied onto a Dionex Ultimate 3000 RSLC nano-HPLC system (Thermo Scientific) equipped with a pre-concentration and desalting cartridge where 0.1% TFA was used as transport liquid. Peptides were separated on a Acclaim PepMap RSLC column (250 mm × 75 μm, C18, 2 μm, 100 Å; Thermo Scientific) through elution with a 59 minute linear gradient from 4 to 45% of solvent B using flow rate of 500 nL/minute (solvent A: 0.1% TFA, solvent B: acetonitrile/ddH_2_O/formic acid, 80/20/0.1% (v/v/v)) and directly subjected to the mass spectrometer. In-line bottom-up proteomics was performed on a QqTOF mass spectrometer oTOF compact from Bruker Daltonics (Billerica, MA, United States) equipped with nano-flow CaptiveSpray ionization device and using the oTOF control software v3.4 (build16). High mass accuracy was achieved by using an internal calibrant (hexakis-(1 H,−1H,−4H-hexafluorobutyloxy)-phosphazine (Agilent Technologies)) during the runs. The cycle time for MS1 and MS/MS fragmentation was 3 seconds, scan range for precursor recording was set to 50−2,200 m/z, spectra rate was set to 2 Hz, dry gas was set to 3 L/minute and the dry temperature was set to 150 °C. The peptide assignment to proteins was conducted with the Proteinscape software v.3.1.5 474 (Build 0140711–1459, Bruker Daltonics) which used the Mascot algorithm version 2.5 (Matrixscience, MA) and peak lists generated with the Compass Data Analysis software v4.2 (Build 395, Bruker Daltonics). Parameters for the protein search were: enzyme specificity: trypsin; species: human; database: UniProtKB/Swiss-Prot; peptide tolerance ± 10 ppm; MS/MS tolerance ±0.05 Da; number of considered 13 C atoms 1; charge states 1 + –3+; up to two missed cleavage; fixed modifications carbamidomethylation (C), variable modifications: deamidation (N, Q), oxidation (M), phosphorylation (S, T, Y), acetylation (K, N-term). The search algorithm probability score was set at p < 0.05.

### Characterisation of the platelet phospho-proteome by λ-phosphatase treatment

To determine phosphorylated protein species, we employed λ-PPase treatment as described previously^30^. Briefly, precipitated gel-filtered platelets aliquots were resolubilised in lysis buffer (7 M Urea, 2 M thiourea, 4% Chaps, 1% DTT), followed by a sonication step (10 minutes, 4 °C), overnight incubation (800 rpm, 4 °C), and determination of the protein concentration using a Coomassie Plus assay kit (Pierce, Thermo Scientific, Rockford, IL, USA). Subsequently, two aliquots à 200 µg of each sample condition (PGI_2_/CTAD) were mixed with 5 µl of 10% SDS. Thereafter, samples were filled up to 500 µl with a reaction mix containing 5 mM DTT, 2 mM MnCl_2_, and 1 × λ-PPase buffer. One PGI_2_ and CTAD aliquot were incubated overnight with 100 units of λ-PPase (30 °C, 300 rpm). Subsequently, all samples (±λ PPase) were precipitated with TCA/DTT.

Detailed description of blood sampling, platelet isolation, *in-vitro* platelet inhibition and activation, 2D-DIGE image analysis, flow cytometry analysis, VASP phosphorylation assay, biological pathway analysis and Western blot analysis is provided in the Supplemental Material and Methods.

### Statistics

Fold changes (FC) of standardised protein values (treatment/IS) were calculated between intervention groups (PGI_2_, CTAD, ADP, TRAP-6) and the untreated control group. Protein spots were considered relevant if 1) spots were matched in more than 90% of all 2D images (n = 24) and 2) if FC were >20% as detected between untreated control and at least one of the intervention groups PGI_2_, CTAD, ADP, TRAP-6. After this filtering, 495 of 5,906 protein spots in the pH range 4–9 were considered relevant. Log-transformed data of 495 spots were evaluated by a repeated ANOVA conducted as a linear mixed model with “group” (untreated control, PGI_2_, CTAD, ADP, TRAP-6) as repeated factor. The multiplicity was controlled by the Benjamini-Hochberg procedure (FDR < 0.05). For a direct comparison of different parameters the effect size was calculated by Cohen’s D = (mean^1^-mean^2^)/standard deviation^pooled^. SPSS Statistics 20.0 (IBM, Chicago, USA) was used for the statistical analysis. Unless stated otherwise, all fold changes represent means.

## Supplementary information

Supplementary Informations

## Data Availability

The datasets generated during and/or analysed during the current study are available from the corresponding authors on reasonable request.
