# Supplementary material for: Comparative proteomics reveals unexpected quantitative phosphorylation differences linked to platelet activation state
Source: Sci Rep. 2019 Dec 12;9:19009. doi: 10.1038/s41598-019-55391-5 (PMC6908631; doi:10.1038/s41598-019-55391-5)
Supplement: Supplementary file 1 — Supplementary Informations [file 41598_2019_55391_MOESM1_ESM.pdf]

Supplementary information

Comparative proteomics reveals unexpected quantitative phosphorylation differences linked to platelet activation state

G.J. Schmidt[1#], C.M. Reumiller[2#], H. Ercan[2], U. Resch[2], E. Butt[3], S. Heber[4], Z. Liutkevičiūte[5], J. Basílio[2], J.A. Schmid[2], A. Assinger[2], B. Jilma[1§] and M. Zellner[2§]

[#]equally contributing authors

Affiliations:
[1]Department of Clinical Pharmacology, Medical University of Vienna, Vienna, Austria
[2]Center for Physiology and Pharmacology, Institute of Vascular Biology and Thrombosis Research, Medical University of Vienna, Vienna, Austria
[3]Institute for Experimental Biomedicine II, University Clinic, Wuerzburg, Germany
[4]Center for Physiology and Pharmacology, Institute of Physiology, Medical University of Vienna, Vienna, Austria
[5]Center for Physiology and Pharmacology, Institute of Pharmacology, Medical University of Vienna, Vienna, Austria

Corresponding authors:
[§]Maria Zellner and [§]Bernd Jilma
E-mail: maria.zellner@meduniwien.ac.at, bernd.jilma@meduniwien.ac.at
Tel.: +43-1-40160-31420; Fax: +43-1-40160 9623; Tel.: +43-1-40400-29810; Fax: +43-1-40400-29980

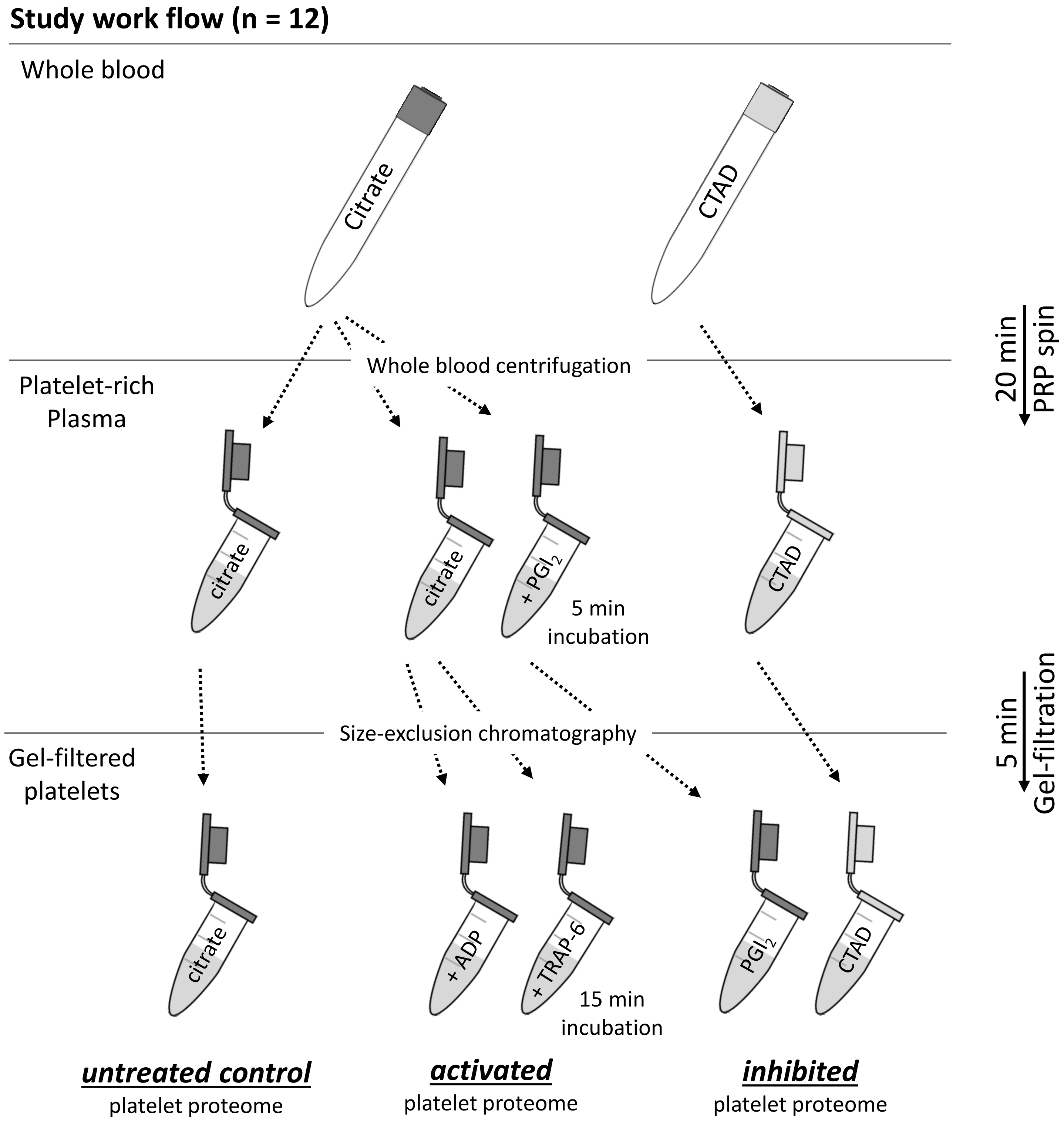

Supplementary Figure S1. Study work flow. Whole blood was drawn into citrate and CTAD blood tubes from 12 healthy volunteers. Blood was then centrifuged at 120 xg for 20 minutes at room temperature to obtain platelet-rich plasma (PRP). For platelet inhibition, PRP generated from citrate-anticoagulated blood was incubated with PGI_2 (0.4 µM final concentration) for 5 minutes prior to gel-filtration (size-exclusion chromatography), while PRP from CTAD-treated whole blood was left untreated. For platelet activation, gel-filtered citrated platelets were incubated with 5 µM ADP and 15 µM TRAP-6 for 15 minutes. Gel-filtered platelets obtained from citrate-anticoagulated whole blood were used as untreated control for activated and inhibited platelet proteome analysis. n = 6 (PGI_2, CTAD), n= 6 (ADP, TRAP-6).

ADP - adenosine-diphosphate, TRAP-6 – thrombin receptor-activating peptide-6, PGI_2 - prostacyclin, CTAD - citrate, theophylline, adenosine, dipyridamole

Supplementary Figure S2

A PRP generated from citrated whole blood was incubated with PGI_2 prior to gel-filtration

B PRP generated from citrated whole blood was gel-filtered and subsequently incubated with PGI_2

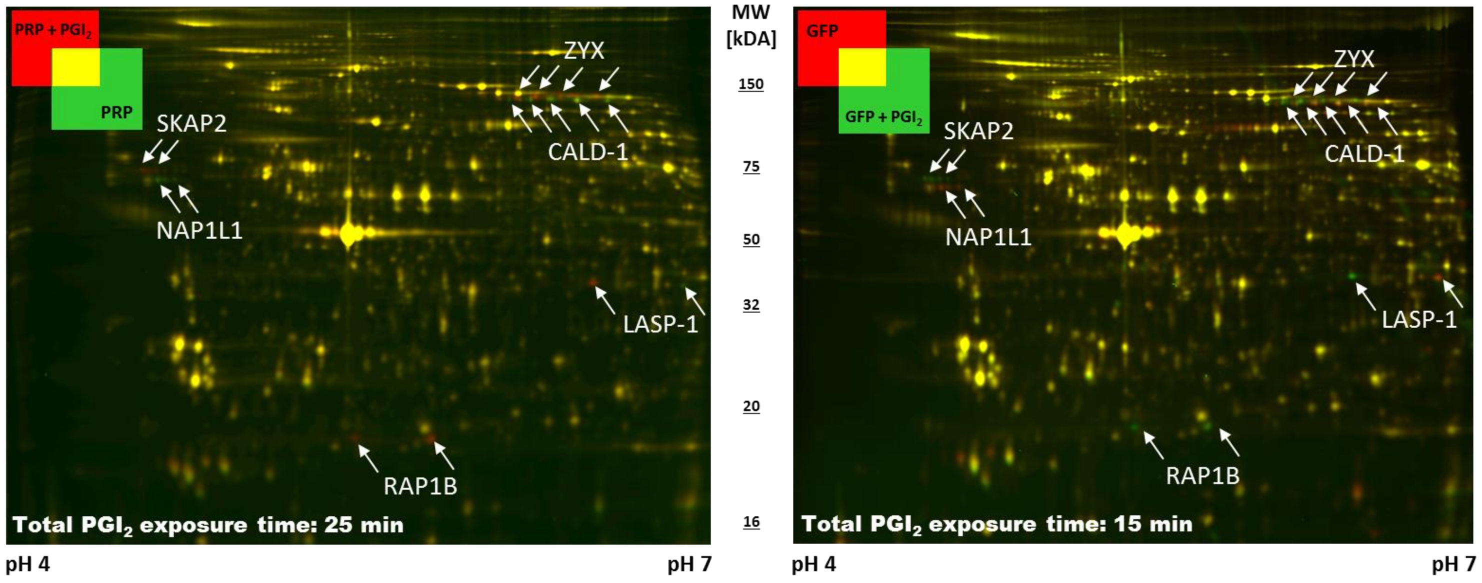

Supplementary Figure S2. Comparison of platelet proteome changes caused by different PGI_2 exposure condition and timing. These exemplary 2D-DIGE images show a selection of significantly changed platelet protein spots upon PGI_2 incubation. To exclude that these observed protein abundance changes derive from platelet handling during gel-filtration, our experimental setup of (**A**) PGI_2 incubation in platelet-rich plasma (PRP) prior to gel-filtration was compared to (**B**) PGI_2 incubation after gel-filtration for 15 minutes in gel-filtered platelets (GFP). A total of 36 µg platelet protein extracts were separated according to the isoelectric point (pI) and the molecular weight (MW) in the pH range 4-7. Protein abundances were normalized by the same internal standard as used in all previous experiments. Platelet inhibition was performed using PGI_2 (0.4 µM final concentration) which is comparable to many platelet function and proteomic studies.

Supplementary Figure S3

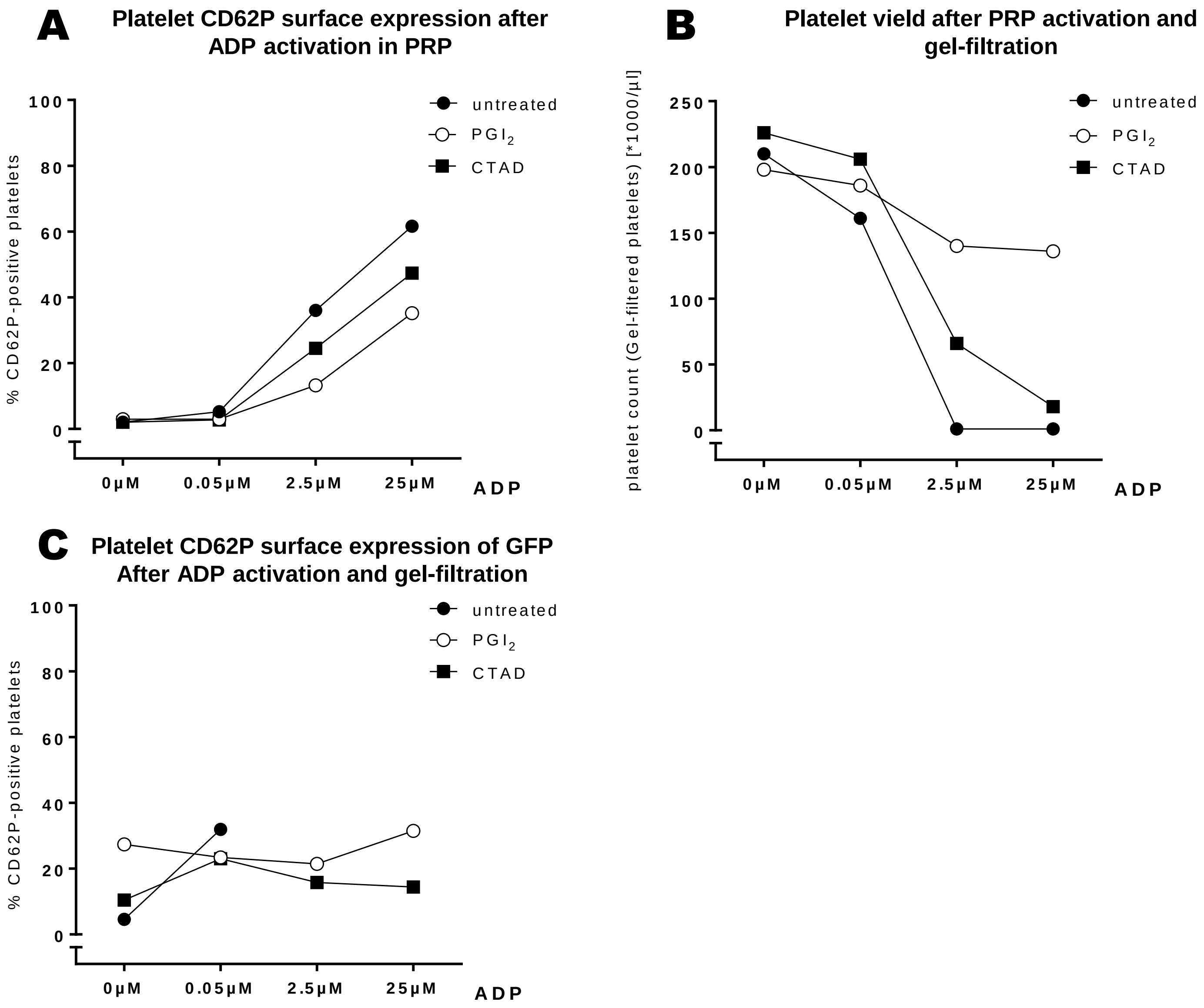

Supplementary Figure S3. Platelet yield and CD62P surface expression after ADP-stimulation of platelet-rich plasma. Platelet-rich plasma (PRP) was obtained by whole-blood centrifugation of citrated and CTAD blood (120 xg for 20 minutes at room temperature). PRP from citrated blood was incubated with PGI_2 (0.4 µM final concentration) for 5 minutes. Citrated PRP, citrated PRP+PGI_2 and CTAD-PRP were then stimulated with three concentrations of ADP (0.05 µM, 2.5 µM, 25 µM) or left untreated as control (0 µM ADP). (**A**) CD62P surface expression of citrate-, PGI_2- and CTAD- treated platelets after ADP challenge. The expression was determined by a phycoerythrin (PE)-labeled antibody against CD62P in one individual. These stimulated platelets were then gel-filtered by sequarose-2B size-exclusion chromatography. (**B**) Corresponding platelet yield after gel-filtration of citrate-, PGI_2- and CTAD- treated platelets (0 µM, 0.05 µM, 2.5 µM and 25 µM ADP). Citrated platelets were not detectable upon 2.5 µM and 25 µM ADP-stimulation via cell counting (Sysmex KX-21N). (**C**) The CD62P surface expression of gel-filtered platelets after ADP-stimulation (0 µM, 0.05 µM, 2.5 µM, 25 µM) in PRP. Values for citrated platelets treated with either 2.5 µM or 25 µM ADP are missing due to an undetectable platelet yield after gel-filtration.

ADP - adenosine-diphosphate, PGI_2 - prostacyclin, CTAD - citrate, theophylline, adenosine, dipyridamole

Supplementary Figure S4

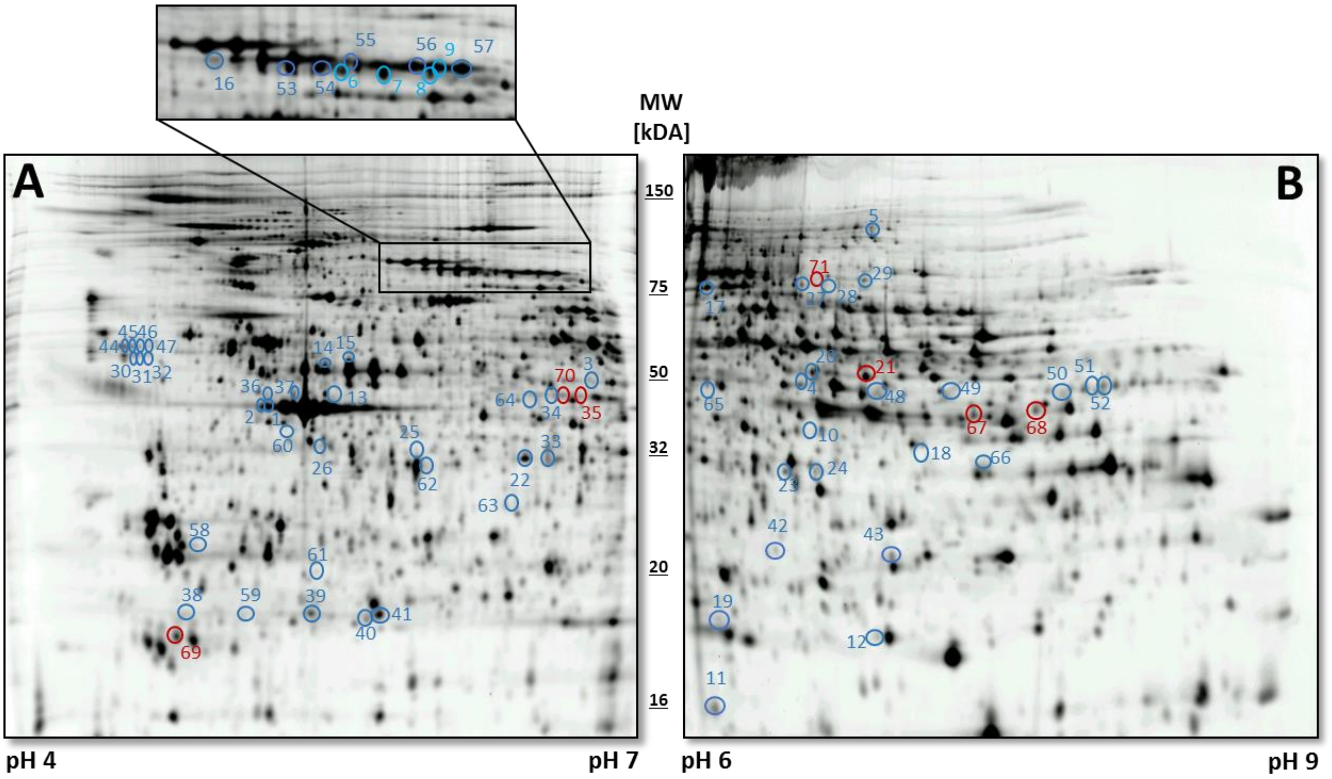

Supplementary Figure S4. Addition to Figure 3 with all significantly altered platelet proteins upon inhibition and activation delineated. These exemplary 2D-DIGE images show all significantly altered platelet protein spots detected upon inhibition and activation. A total of 36 µg platelet protein extracts were separated according to the isoelectric point (pI) and the molecular weight (MW) in the pH ranges (**A**) 4-7 and (**B**) 6-9. Altered proteins were identified by mass spectrometry and are outlined in blue for inhibition and in red for activation. Protein spots were numbered consecutively according to *Supplementary Table S1* (protein identification #) and fit the following criteria (a) protein spots matched > 90% of all 2D-DIGE gels, (b) protein abundance changes > 20% between treatment group and citrated control and (c) FDR-corrected ANOVA p-value < 0.05. Protein spots °21 and °35 were both significantly affected by activation and inhibition.

Supplementary Figure S5

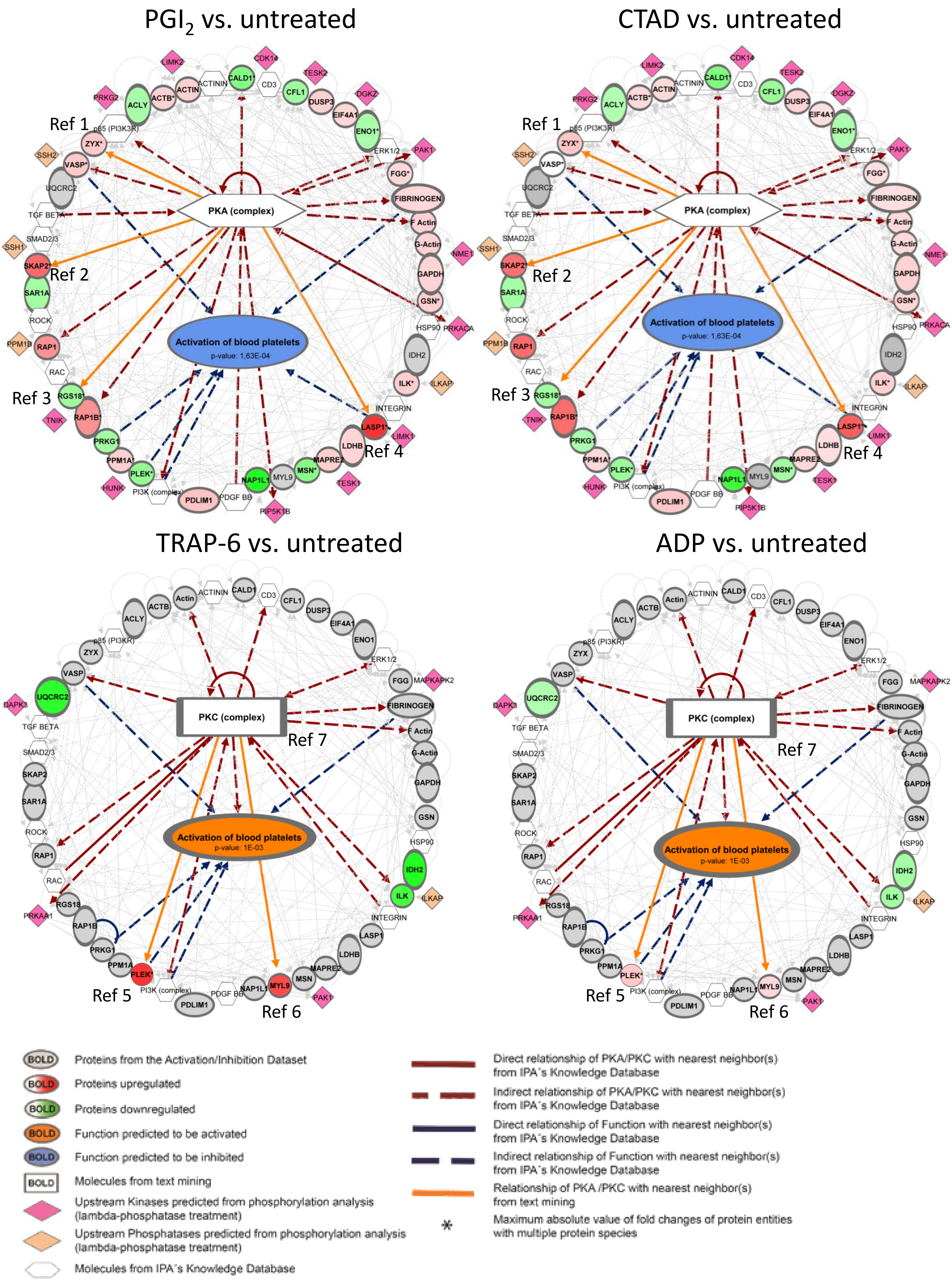

Supplementary Figure S5. Network analysis of inhibited and activated platelet proteins. Top network linked to differentially regulated (**A,B**) inhibition-dependent and (**C,D**) activation-dependent platelet proteins upon integrated analysis with the IPA software and corresponding p-values for the enriched biological function term. Proteins with ellipse shaped nodes are proteins from the inhibition-and activation datasets. Proteins with a hexagonal form were joined through the network analysis from the Ingenuity Pathway Knowledge Database (QIAGEN Inc., https://www.qiagenbioinformatics.com/products/ingenuity-pathway-analysis). In diamond shape are the nodes which resulted from the upstream regulators module of the phosphorylation analysis of the λ-PPase+PGI_2 - and λ-PPase+CTAD treated samples. In rectangular shapes, the nodes which resulted from text mining. Edges connecting the central nodes, PKA or PKC, and the enriched function to their nearest neighbours are highlighted. All edges are supported by at least one reference from the literature, or from canonical information stored in the Ingenuity Pathways Knowledge Base. The lines between molecules represent known interactions, with solid lines representing direct interactions and dashed lines representing indirect interactions.

PGI_2 - prostacyclin, CTAD - citrate, theophylline, adenosine, dipyridamole, ADP - adenosine-diphosphate, TRAP-6 – thrombin receptor-activating peptide-6, λ-PPase - lambda phosphatase, pI -isoelectric point, PKA - protein kinase A, PKC - protein kinase C, IPA - Ingenuity Pathway Analysis

Supplementary Figure S6

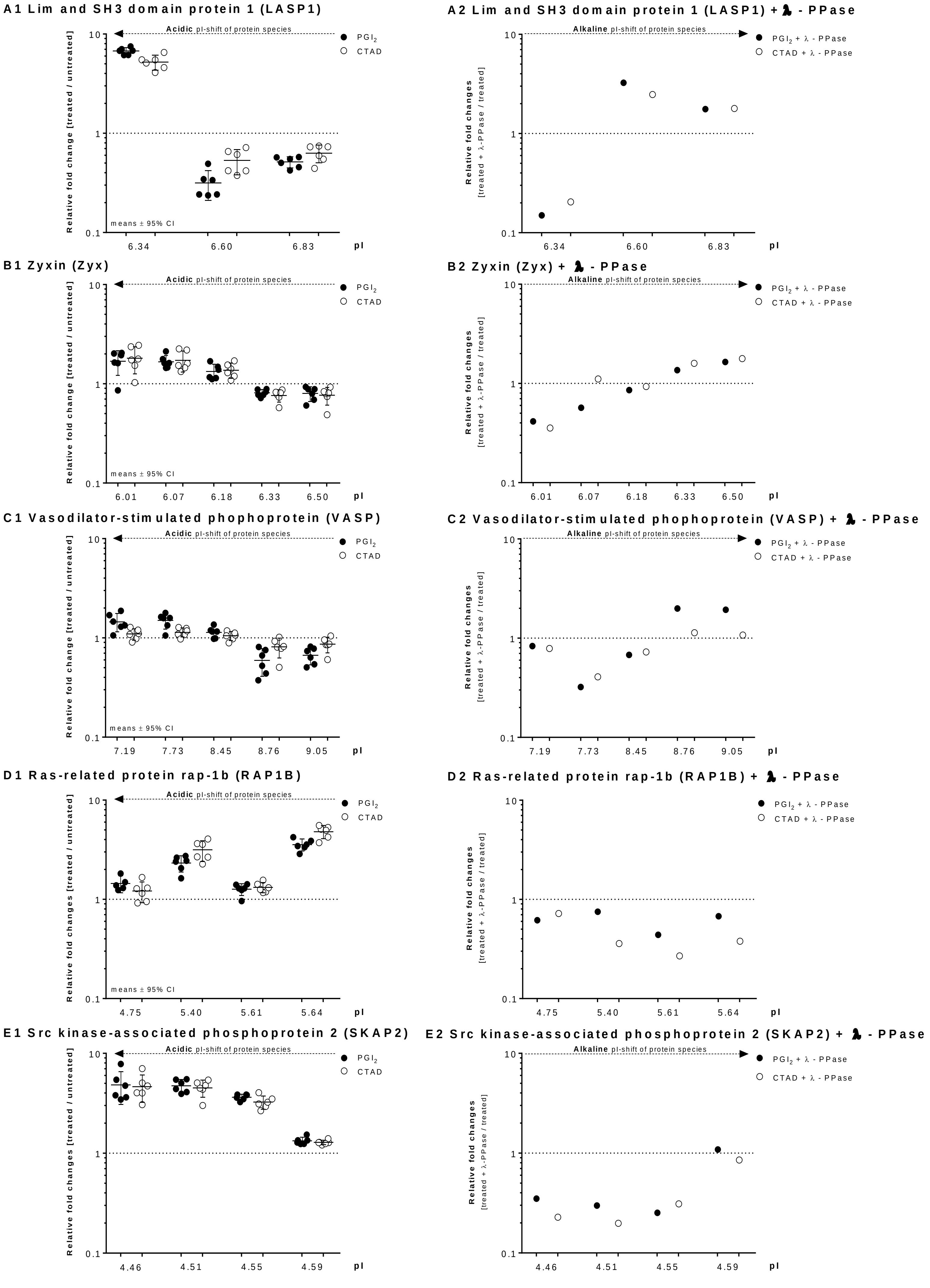

Supplementary Figure S6. Detection of phosphorylated protein species of the known cAMP/PKA targets LASP1, ZYX, VASP, RAP1B and potential cAMP/PKA target SKAP2 by λ-phosphatase treatment. (**A**) Upon inhibition (PGI_2, CTAD) significantly changed protein species of known and potential cAMP/PKA targets LASP1, ZYX, VASP RAP1B and SKAP2 are depicted along their pI ranges. Values of each protein species are given as relative fold changes between treated gel–filtered platelets (PGI_2, CTAD) and untreated control (dashed line y = 1) with their means and 95% confidence interval (CI). For LASP1, ZYX, VASP and SKAP2 an acidic pI shift of protein species upon inhibition was detectable. (**B**) To determine if a possible post-translational phosphorylation of proteins is responsible for this shift, PGI_2- and CTAD- treated gel-filtered platelet were incubated with λ-phosphatase (λ-PPase) and compared to PGI_2- and CTAD-treated gel-filtered platelets, respectively. The λ-PPase removes the phosphorylation from serine, threonine and tyrosine residues, thereby shifting its isoelectric point (pI) to the alkaline direction, displaying a mirror-inverted picture to the abundance changes of protein species seen in (**A**). The phosphorylation profile of RAP1B was additionally validated via 1D Western blot (*Supplementary Figure S7*). The relative fold changes between PGI_2/CTAD+λ-PPase and PGI_2/CTAD-treated platelets [PGI_2/CTAD+λ-PPase / PGI_2 or CTAD] are depicted (n = 1).

PGI_2 - prostacyclin, CTAD - citrate, theophylline, adenosine, dipyridamole, λ-PPase - lambda phosphatase, pI - isoelectric point, cAMP/PKA - cyclic adenosine monophosphate / protein kinase A

A. 2D-DIGE Proteome of untreated control

Supplementary Figure S7

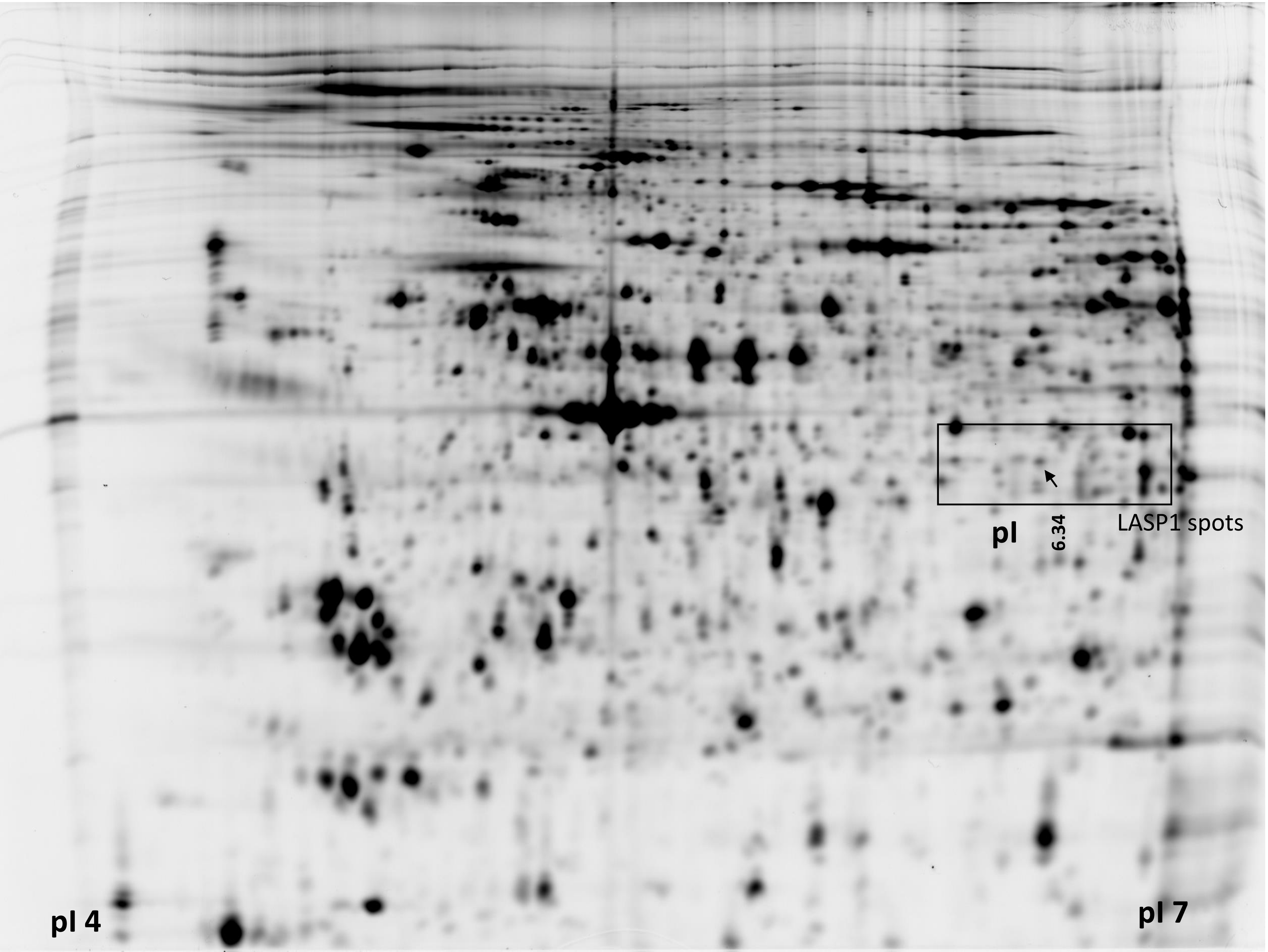

A. 2D-DIGE Proteome of untreated control

Supplementary Figure S7

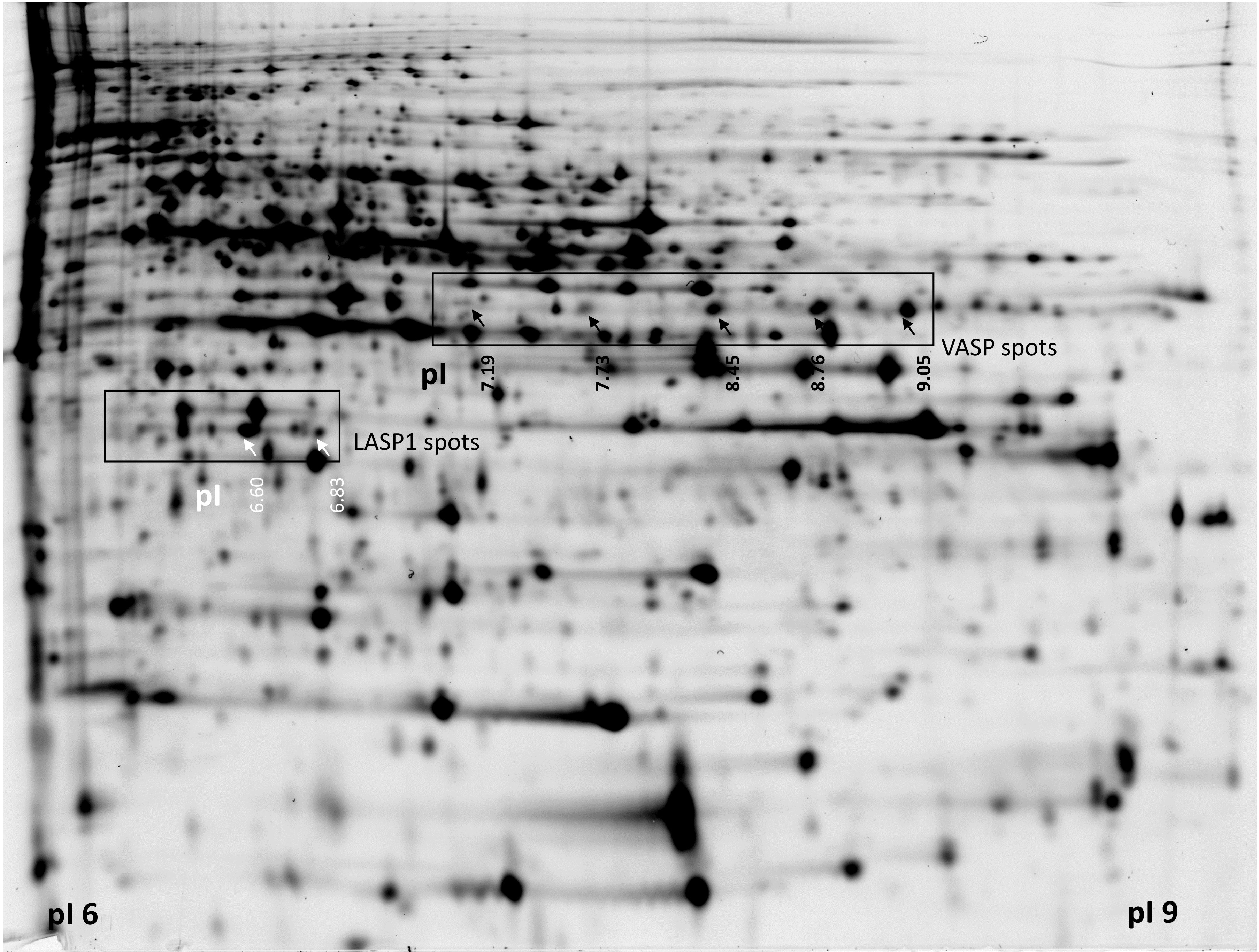

Supplementary Figure S7

1: Vasodilator-stimulated phosphoprotein (VASP)

A1a. 2D-WB: Cy2 –labeled platelet proteins – untreated control

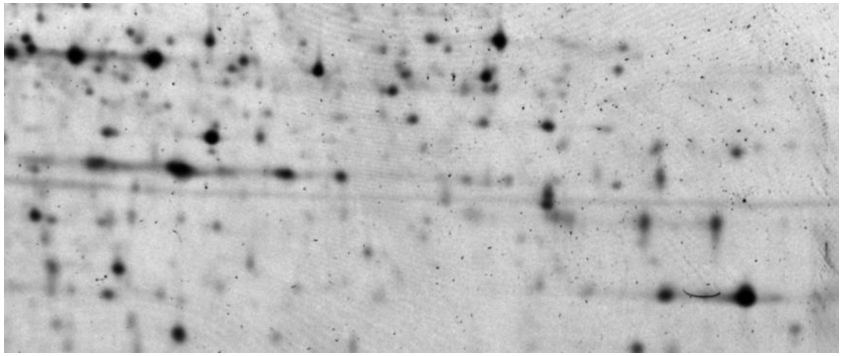

A1a. 2D-WB: VASP spots detected by **pan-VASP antibody** – untreated control

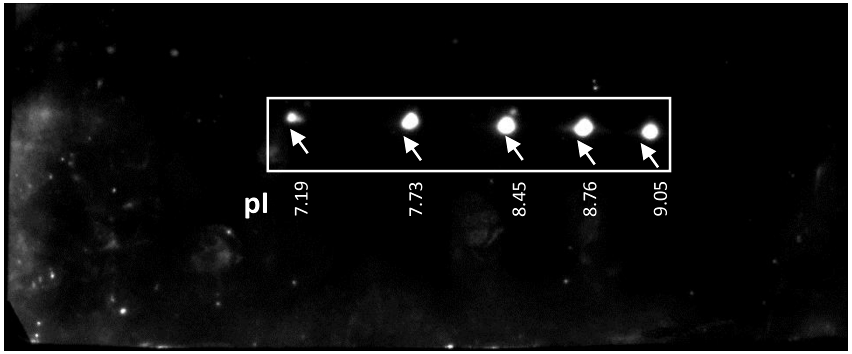

A1a. 2D-WB: Cy2 –labeled platelet proteins overlaid with VASP spots detected by **pan-VASP antibody** – untreated control

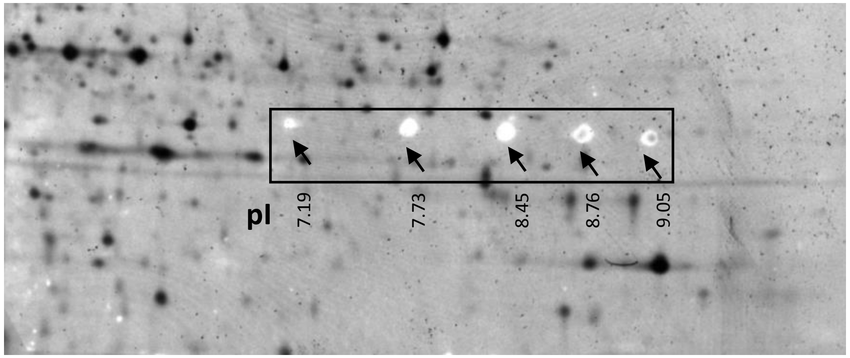

A1b. 2D-WB: 2D-DIGE gel (Cy2) – untreated control

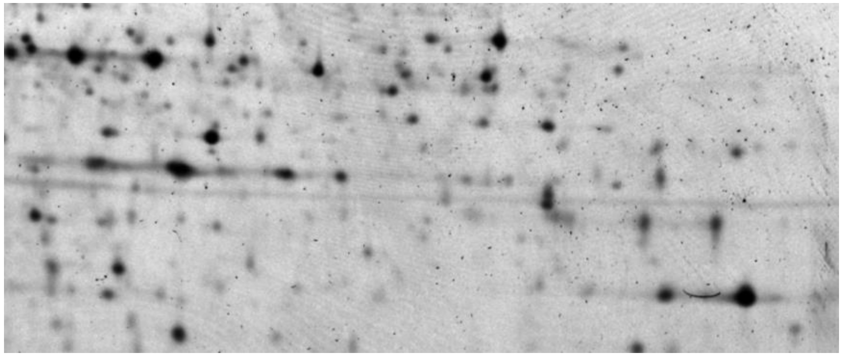

A1b. 2D-WB: VASP spots detected by **pVASP-Ser^{239} antibody** – untreated control

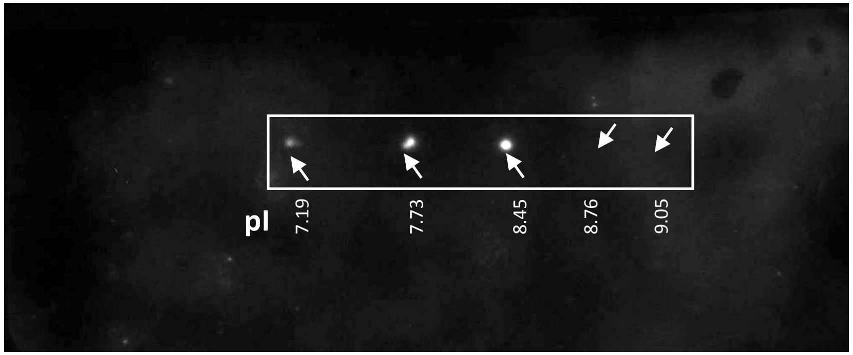

A1b. 2D-WB Cy2 –labeled platelet proteins overlaid with VASP spots detected by **pVASP-Ser^{239} antibody** – untreated control

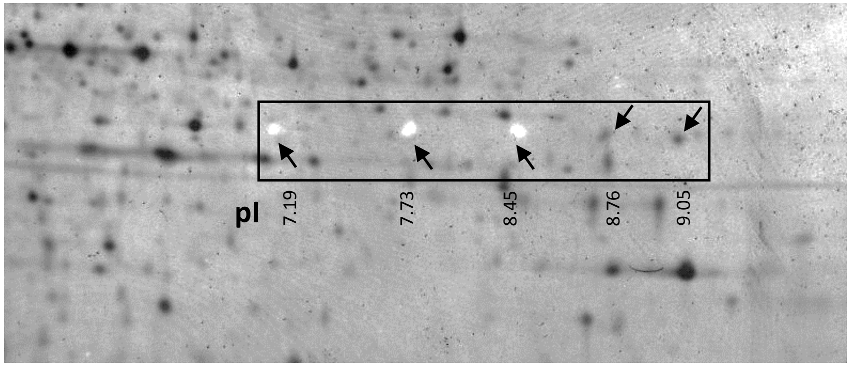

1: Vasodilator-stimulated phosphoprotein (VASP)

Supplementary Figure S7

A1a. 2D-WB: Cy2 –labeled platelet proteins – untreated control

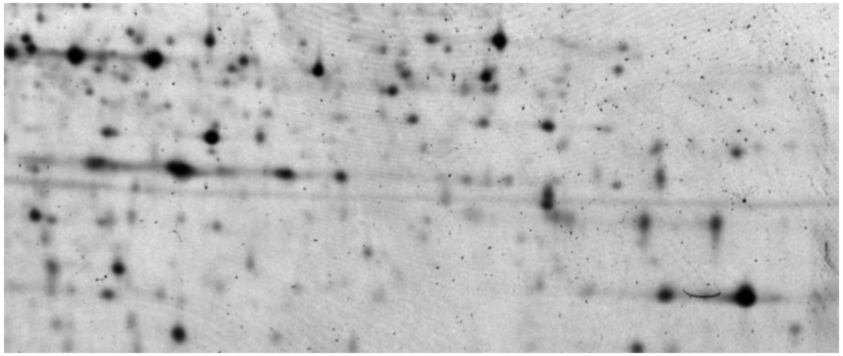

A1a. 2D-WB: VASP spots detected by **pan-VASP antibody** – untreated control

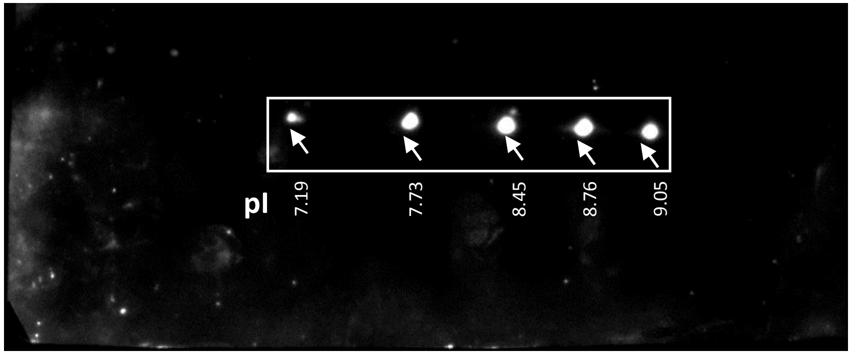

A1a. 2D-WB: Cy2 –labeled platelet proteins overlaid with VASP spots detected by **pan-VASP antibody** – untreated control

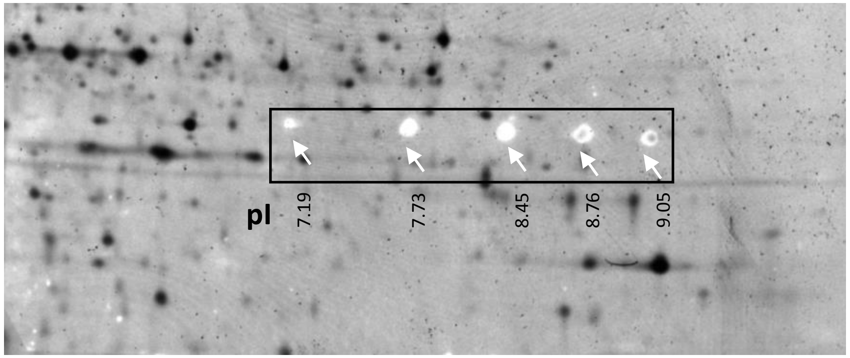

A1c. 2D-WB: Cy2 –labeled platelet proteins – untreated control

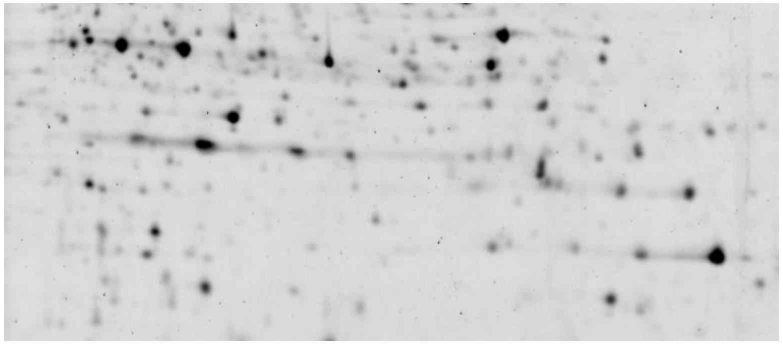

A1c. 2D-WB: VASP spots detected by **pVASP-Ser157 antibody** – untreated control

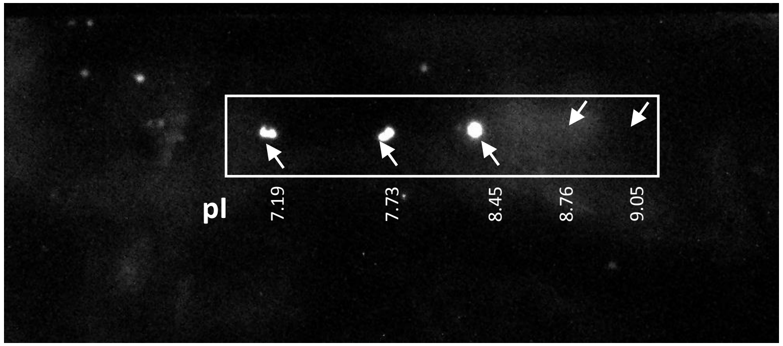

A1c. 2D-WB: Cy2 –labeled platelet proteins overlaid with VASP spots detected by **pVASP-Ser157 antibody** – untreated control

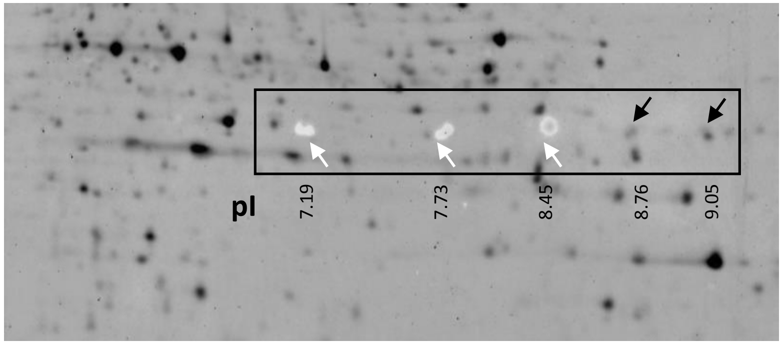

2: LIM and SH3 domain protein 1 (LASP1)

Supplementary Figure S7

A2a. 2D-WB: Cy2 –labeled platelet proteins – untreated control

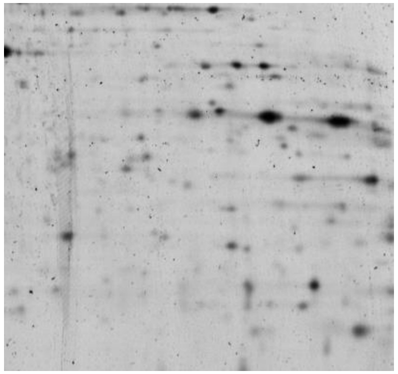

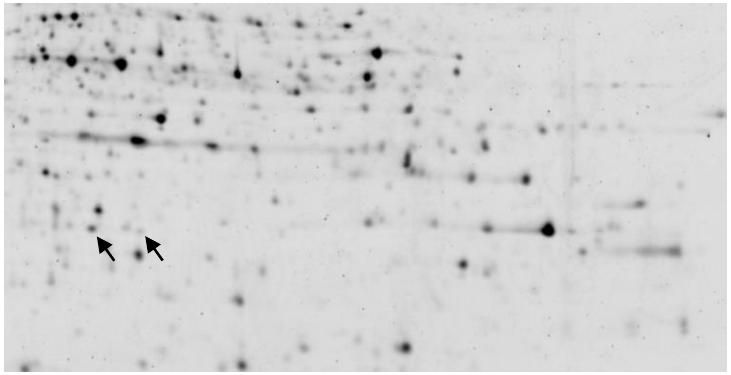

A2a. 2D-WB: : LASP1 spots detected by **pan-LASP1 antibody** – untreated control

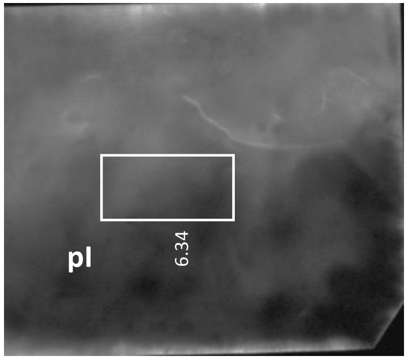

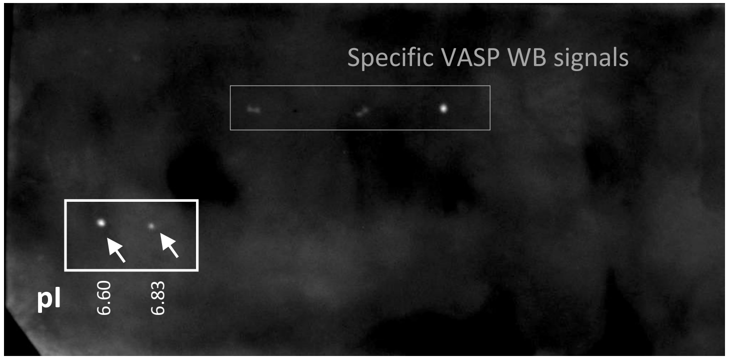

A2a. 2D-WB: Cy2 –labeled platelet proteins overlaid with LASP1 spots detected by **Pan-LASP1 antibody** – untreated control

no spots detected
in untreated control
pH 4 -7

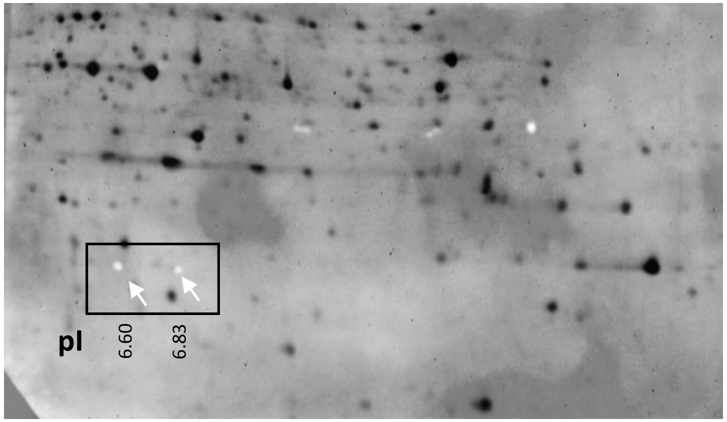

2: LIM and SH3 domain protein 1 (LASP1)

Supplementary Figure S7

A2b. 2D-WB: Cy2 –labeled platelet proteins – <u>untreated control</u>

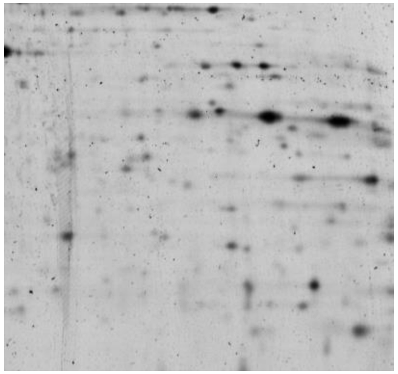

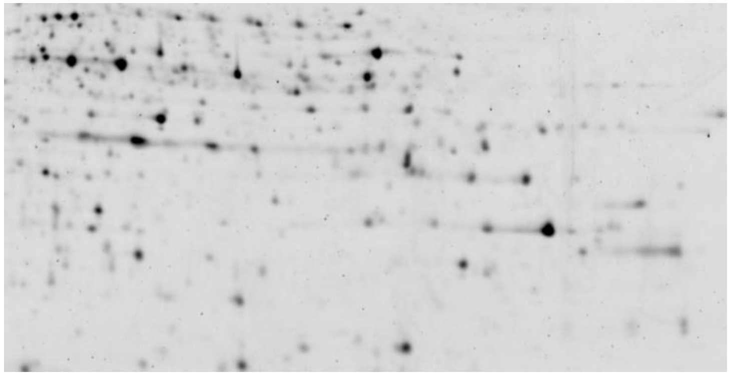

A2b. 2D-WB: no LASP1 spots detected by **pLASP1-Ser^{146} antibody** – <u>untreated control</u>

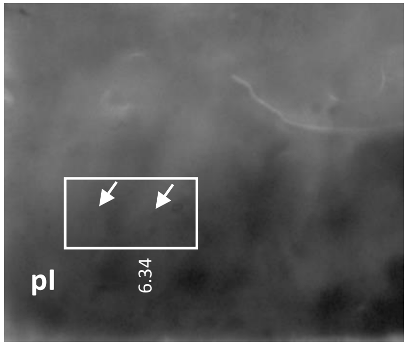

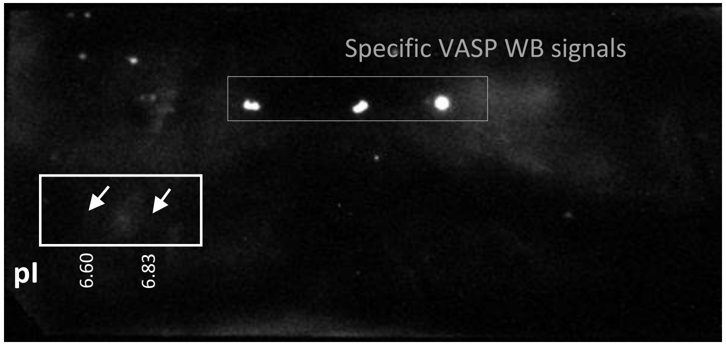

no spots detected
in untreated control
pH 4 - 7

no spots detected
in untreated control
pH 6 - 9

B. 2D-DIGE Proteome of PGI_2 treated platelets **Supplementary Figure S7**

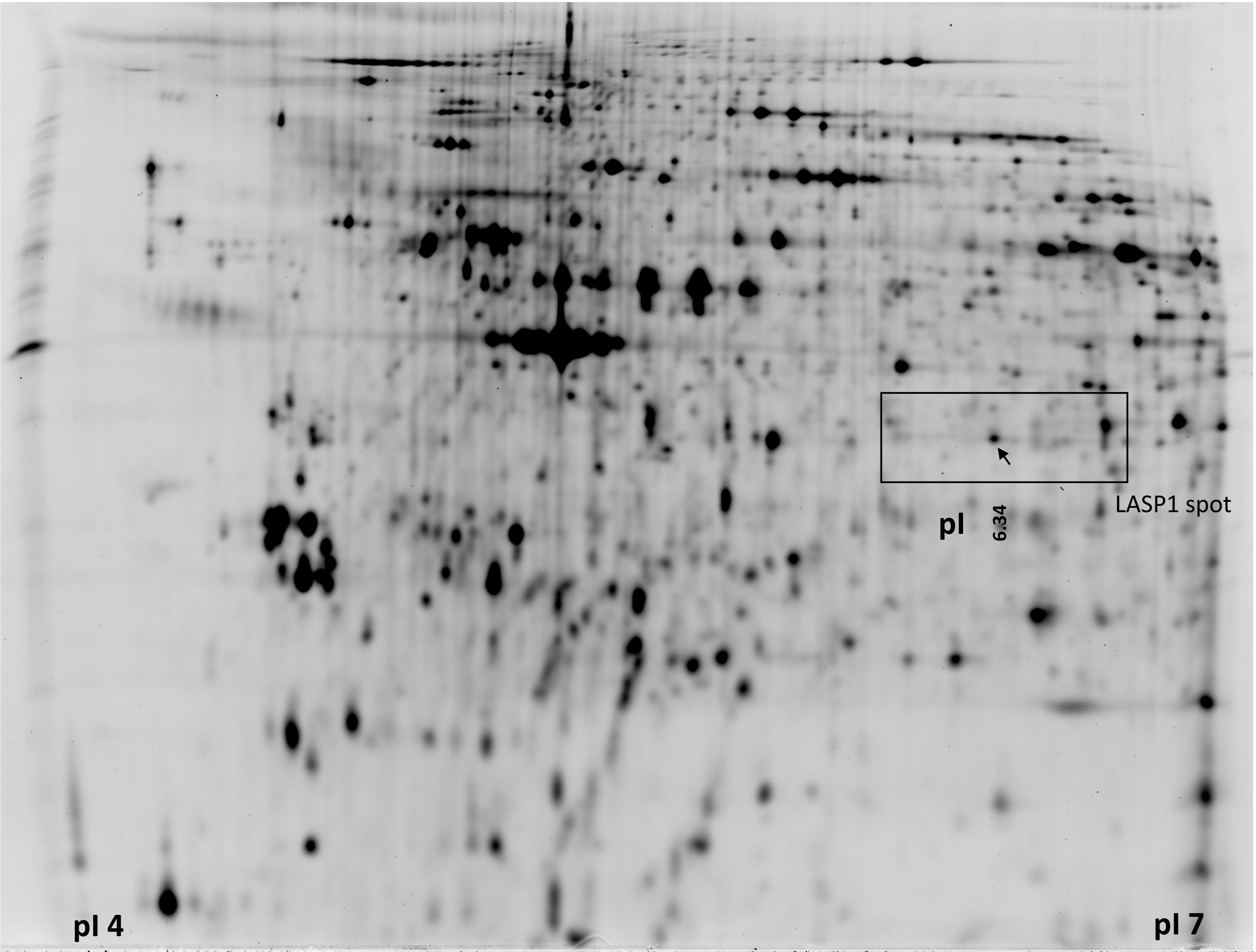

B. 2D-DIGE Proteome of PGI$_2$ treated platelets

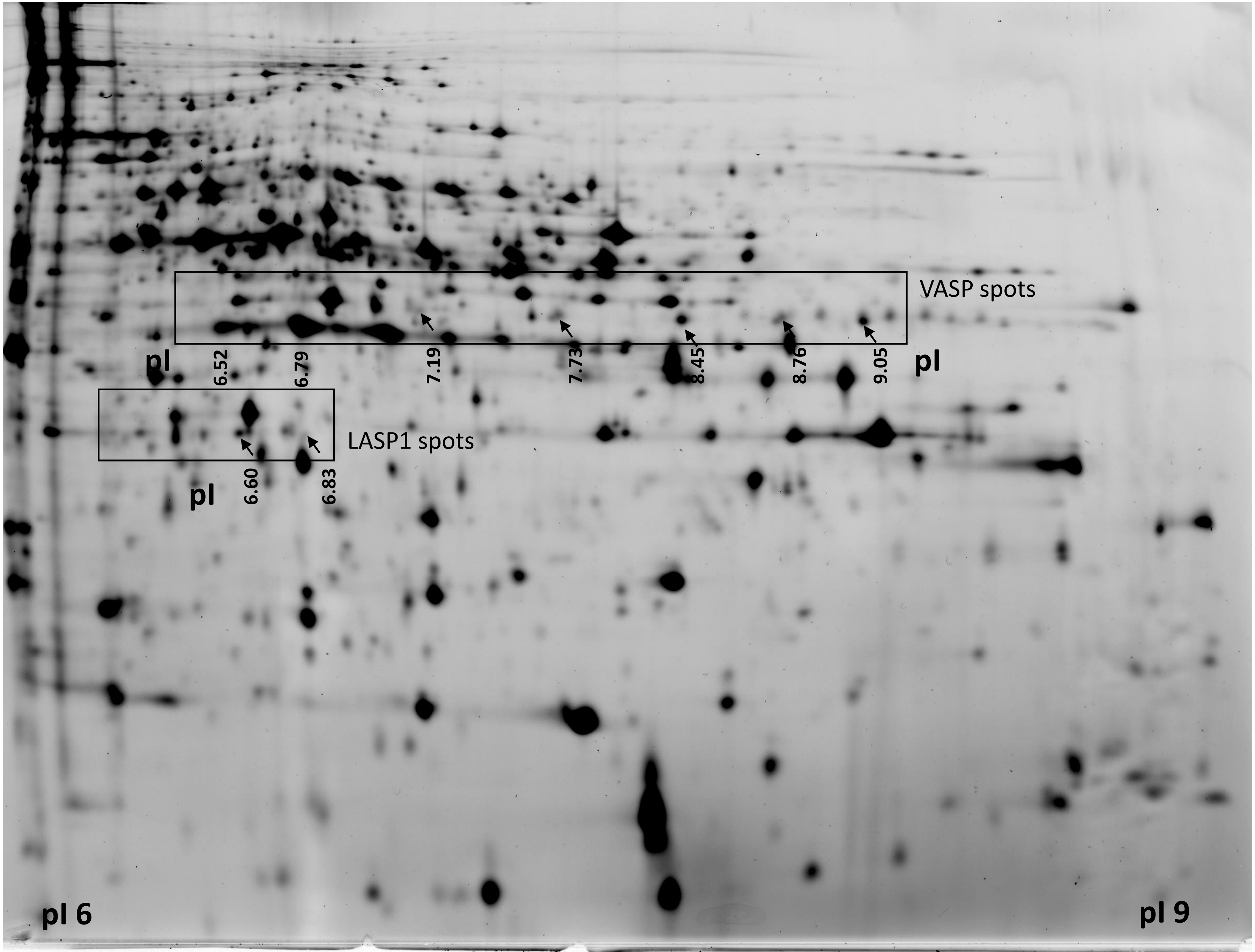

1: Vasodilator-stimulated phosphoprotein (VASP)

Supplementary Figure S7

B1a. 2D-WB: Cy2 –labeled platelet proteins – PGI_2 treated

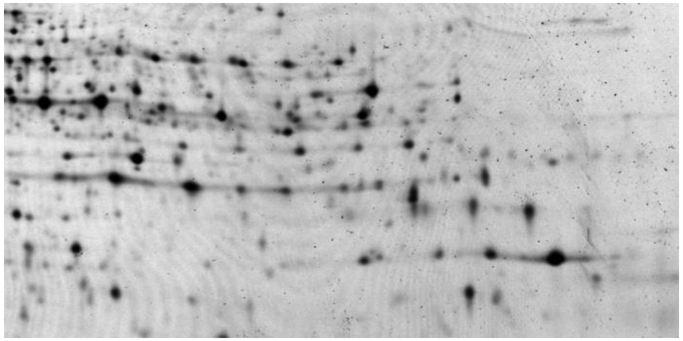

B1a. 2D-WB: VASP spots detected by **pan-VASP antibody** - PGI_2 treated

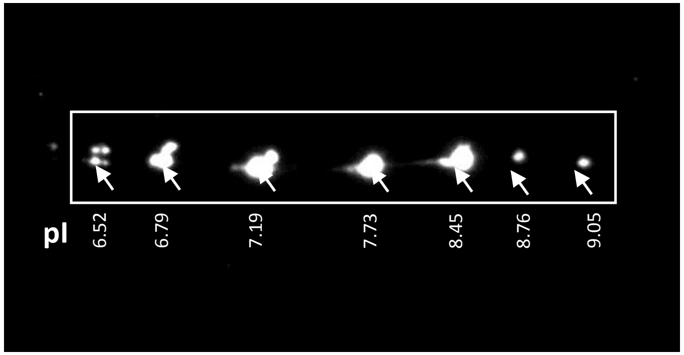

B1a. 2D-WB: Cy2 –labeled platelet proteins overlaid with VASP spots detected by **pan-VASP antibody** - PGI_2 treated

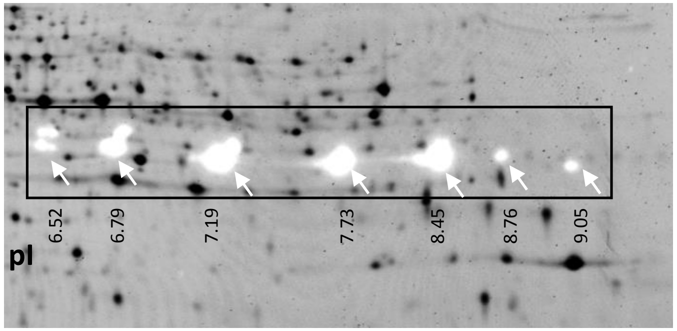

B1b. Cy2 –labeled platelet proteins – PGI_2 treated

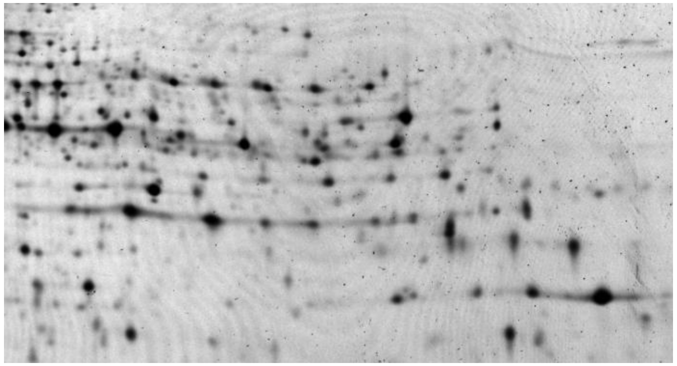

B1b. 2D-WB: VASP spots detected by **pVASP-Ser^{239} antibody** - PGI_2 treated

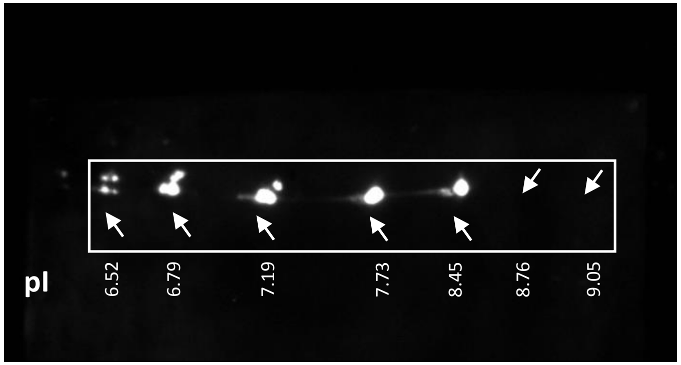

B1b. 2D-WB: Cy2 –labeled platelet proteins overlaid with VASP spots detected by **pVASP-Ser^{239} antibody** - PGI_2 treated

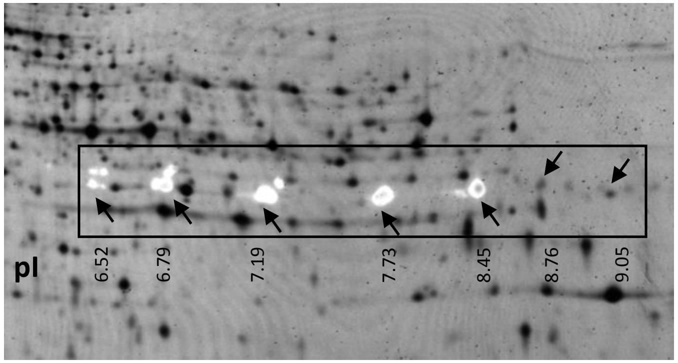

1: Vasodilator-stimulated phosphoprotein (VASP)

Supplementary Figure S7

B1a. 2D-WB: Cy2 –labeled platelet proteins – PGI$_2$ treated

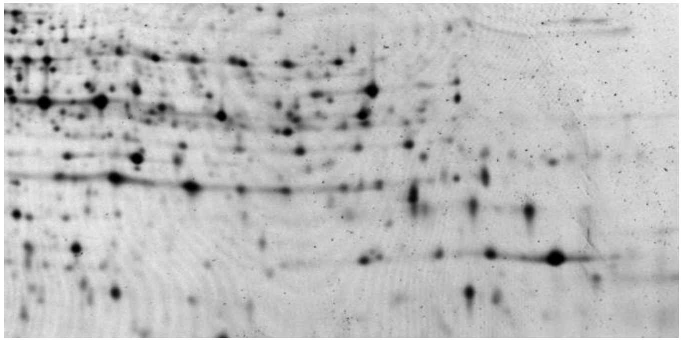

B1a. 2D-WB: VASP spots detected by **pan-VASP antibody** - PGI$_2$ treated

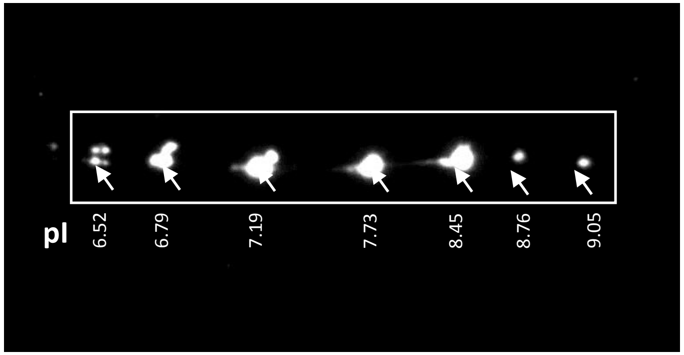

B1a. 2D-WB: Cy2 –labeled platelet proteins overlaid with VASP spots detected by **pan-VASP antibody** - PGI$_2$ treated

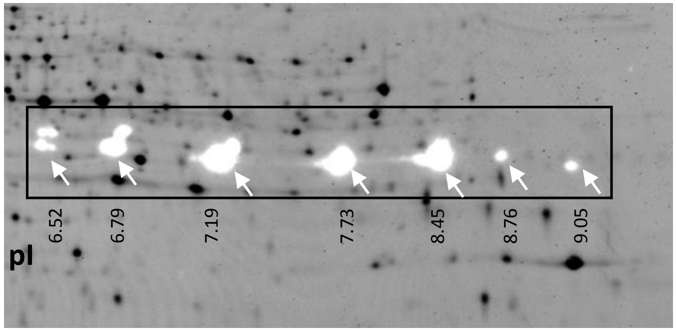

B1c. 2D-WB: Cy2 –labeled platelet proteins – PGI$_2$ treated

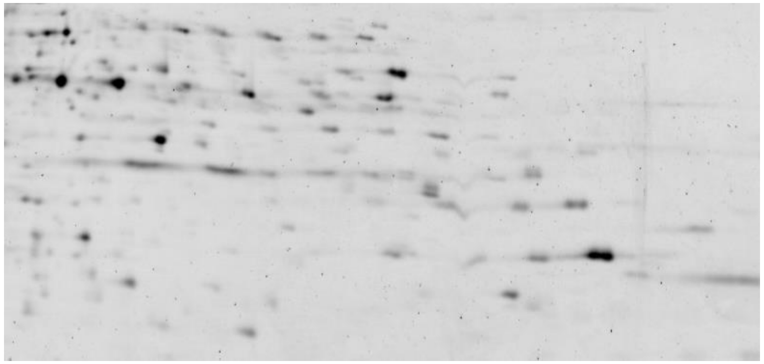

B1c. 2D-WB: VASP spots detected by **pVASP-Ser^{157} antibody** - PGI$_2$ treated

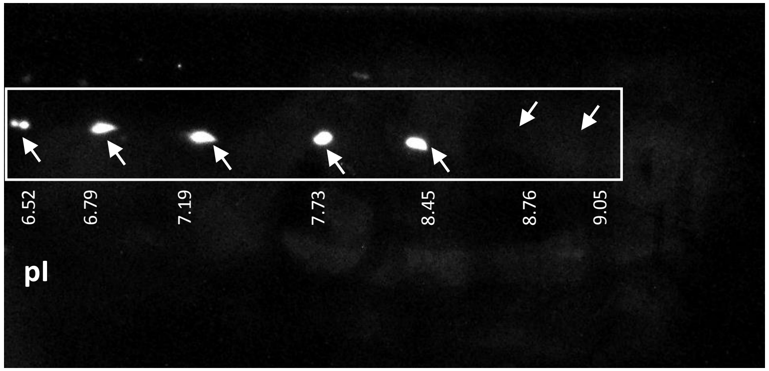

B1c. 2D-WB: : Cy2 –labeled platelet proteins overlaid with VASP spots detected by **pVASP-Ser^{157} antibody** - PGI$_2$ treated

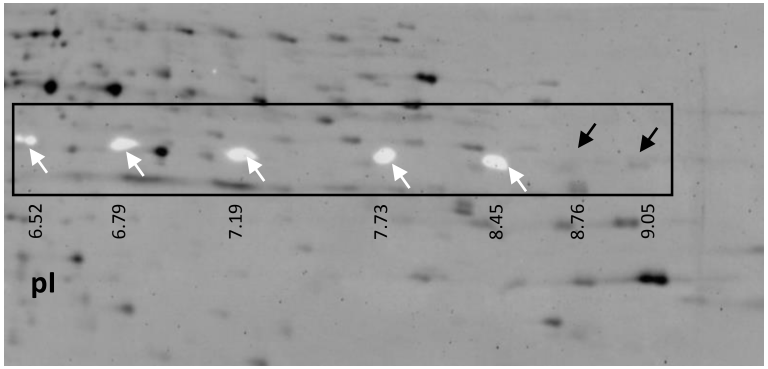

2: LIM and SH3 domain protein 1 (LASP1)

Supplementary Figure S7

B2a. 2D-WB: Cy2 – labeled platelet proteins - PGI$_2$ treated

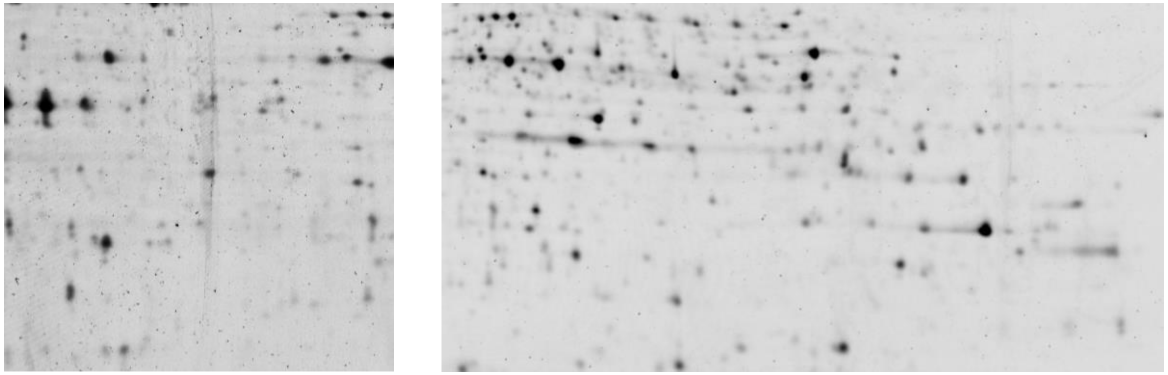

B2a. 2D-WB: LASP1 spots detected by **pan-LASP1 antibody** - PGI$_2$ treated

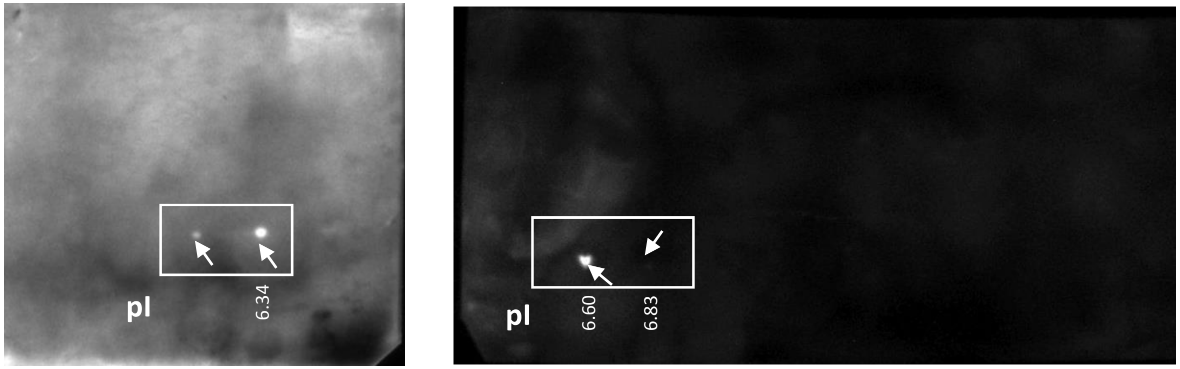

B2a. 2D-WB: Cy2 – labeled platelet proteins overlaid with LASP1 spots detected by **pan-LASP1 antibody** - PGI$_2$ treated

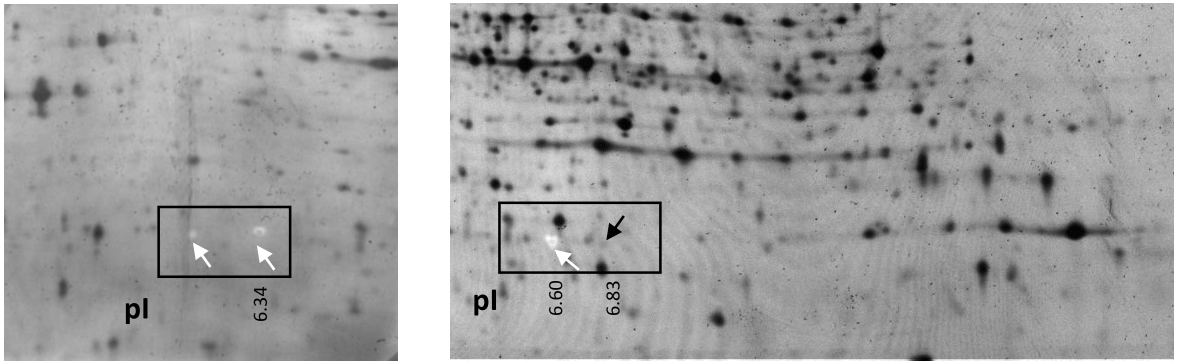

2: LIM and SH3 domain protein 1 (LASP1)

Supplementary Figure S7

B2b. 2D-WB: Cy2 – labeled platelet proteins - PGI$_2$ treated

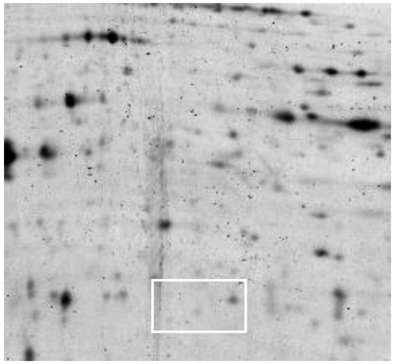

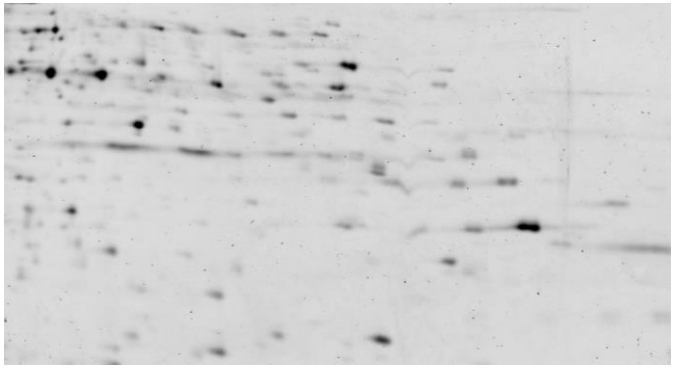

B2b. 2D-WB: LASP1 spots detected by **pLASP1-Ser^{146} antibody** - PGI$_2$ treated

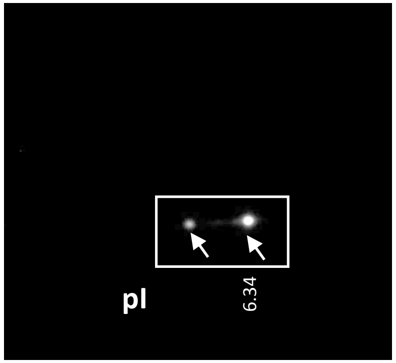

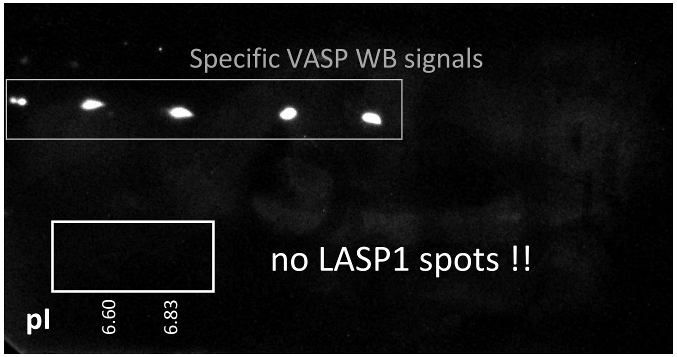

B2b. 2D-WB: Cy2 – labeled platelet proteins overlaid with LASP1 spots detected by **pLASP1-Ser^{146} antibody** - PGI$_2$ treated

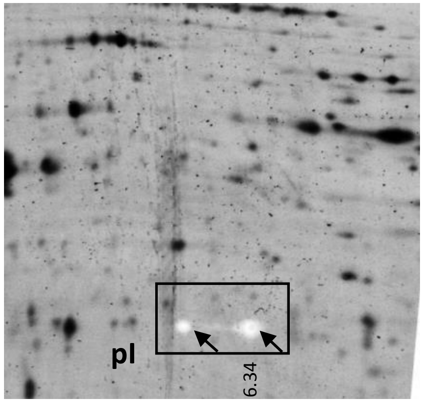

no spots detected
in PGI$_2$ treated control
pH 6 - 9

C. Proteome PGI_2 treated platelets + λ-PPase treatment

Supplementary Figure S7

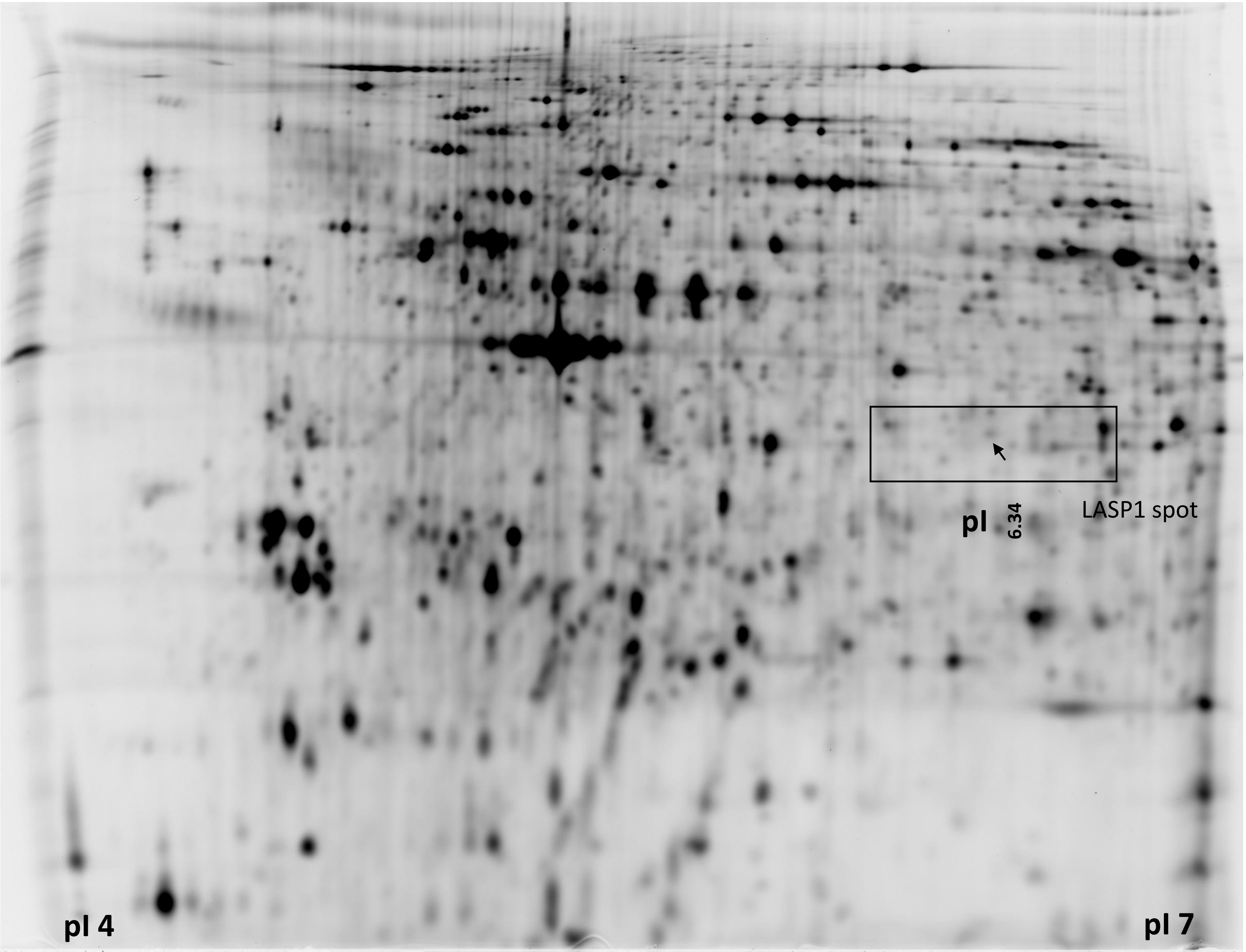

C. Proteome of PGI_2 treated platelets + λ-PPase treatment

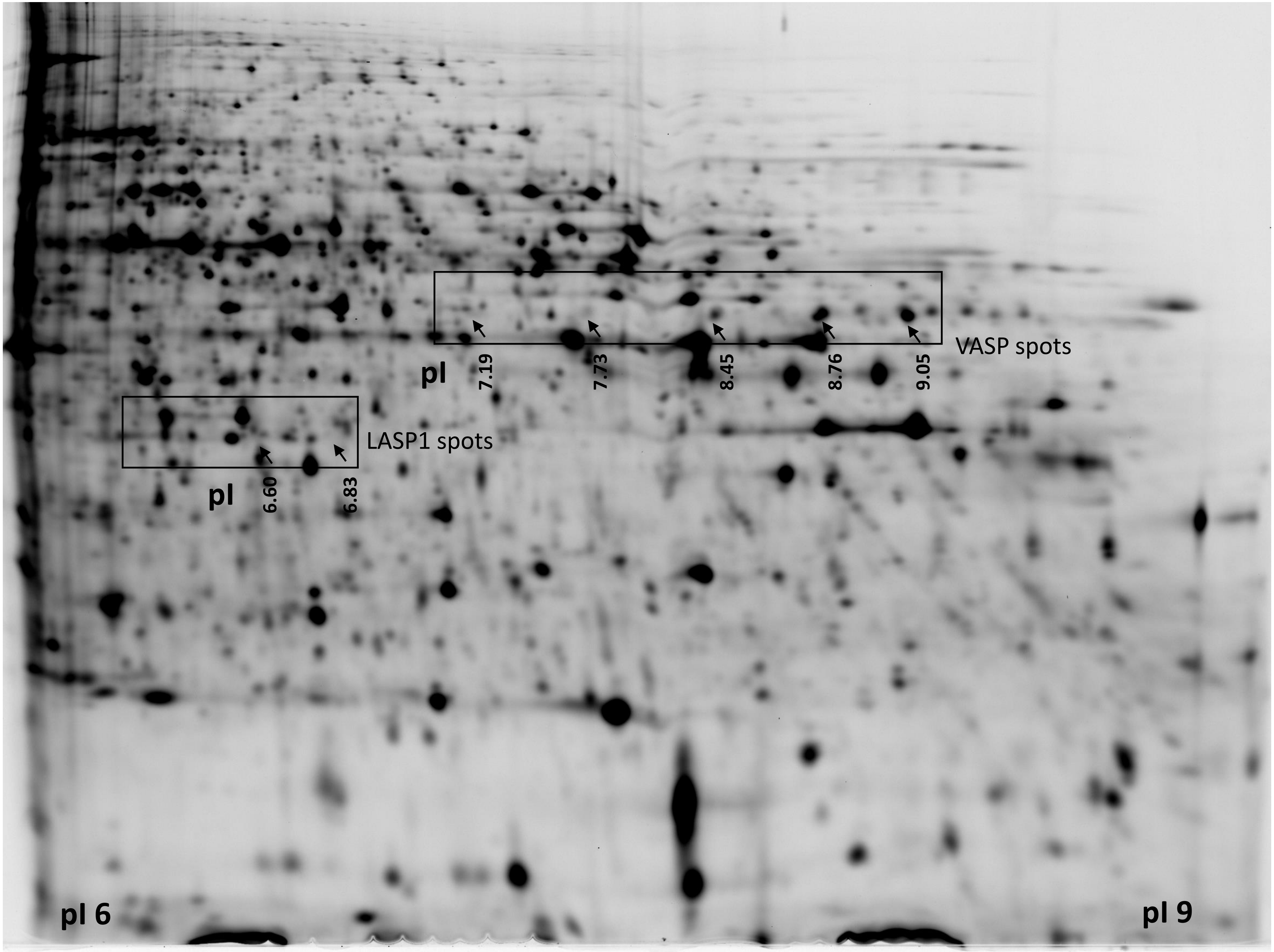

Supplementary Figure S7. Full-length images of 2D-DIGE and 2D Western blot details from figure 5 - Detection of phosphorylated platelet protein species of VASP and LASP1 by pan, phospho-site specific antibodies and λ-phosphatase treatment. Spot abundances of (**1**) VASP spots and (**2**) LASP1 spots are shown in (A) untreated control, (B) PGI_2-, and (C) PGI_2+λ-phosphatase treated platelets along their pI ranges, detectable by 2D-DIGE analysis in the pH range 4-7 and 6-9. (**A**) Platelet protein extracts from citrated anticoagulated blood constitute baseline condition. (**B**) PGI_2-treatment shifts increased protein spot abundances towards the acidic pH-range with a concomitant reduction of the corresponding alkaline spot abundances. (**1**) VASP and (**2**) LASP1 spots are indicated from CyDye-labeled and two-dimensional separated platelet proteins. Detection of primary antibody binding towards blotted platelet protein was done with horse-radish peroxidase (HRP) conjugated secondary antibody. Proteoforms immunodetected and HRP generated chemoluminescence signal are shown in white. Antibody signals were exactly overlaid with CyDye-labeled two-dimensional separated platelet proteom to evaluate which protein spots are recognised by the pan as well as phosphosite specific antibodies. (**C**) Λ-phosphatase treatment removes the phosphorylation of respective protein species thereby shifting its isoelectric point (pI) towards the alkaline direction. Images (A-C) constitute a representative picture from one individual platelet sample. The gel-sections were obtained from scanned 2D gels and 2D WBs the respective protein area are indicated within the plotted rectangle.

VASP - Vasodilator-stimulated phosphoprotein, LASP1 - LIM and SH3 domain protein 1, λ-PPase -lambda phosphatase, pI - isoelectric point

Supplementary Figure S8

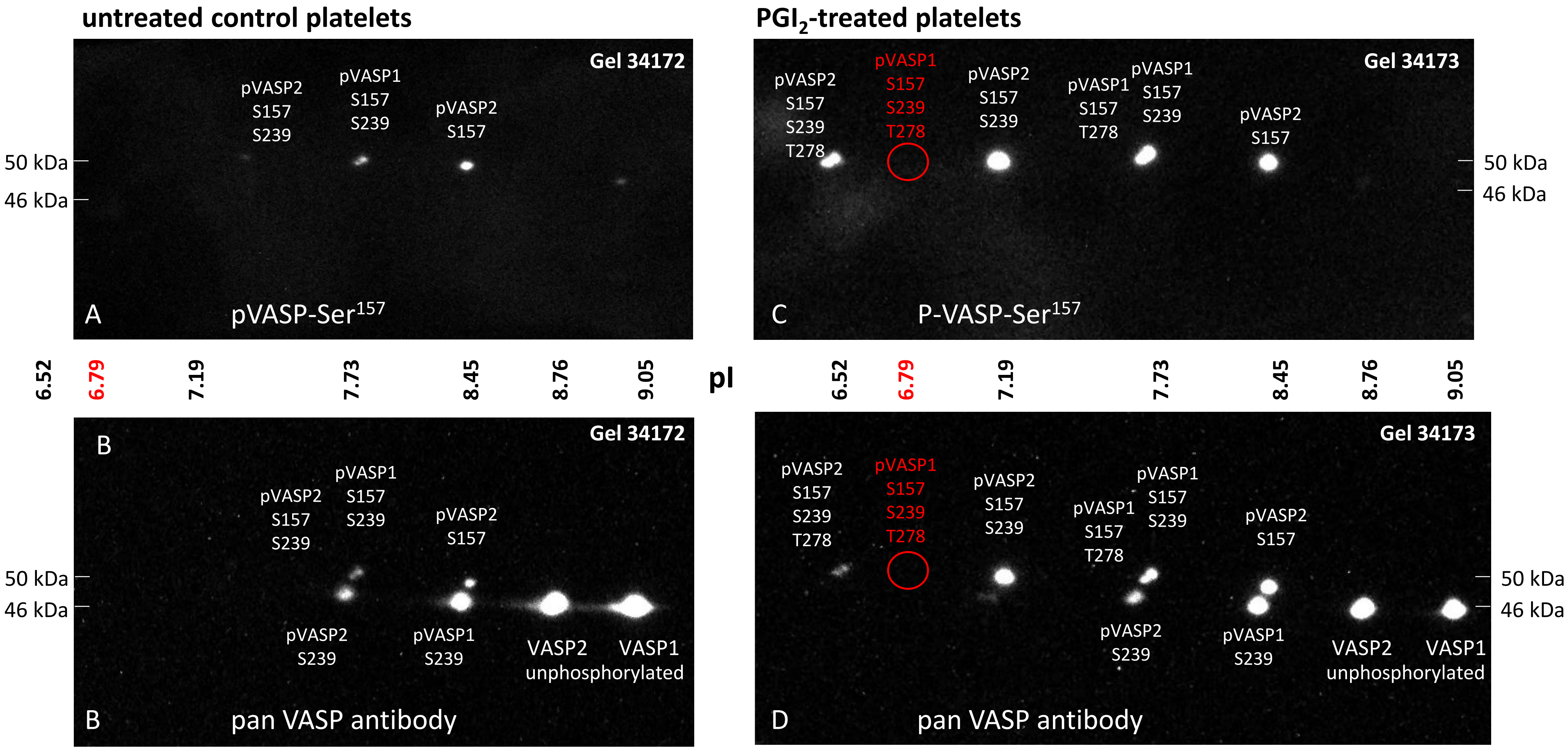

Supplementary Figure S8. High resolution 2D Western blot from VASP proteoforms by 12.5 % polyacrylamide. (**A+B**) TCA-precipitated GFP protein extracts from citrated anticoagulated blood constitute the untreated control. (**C+D**) PGI_2-treatment shifts increased protein spot abundances towards the acidic pH-range with a concomitant reduction of the corresponding alkaline spot abundances. Detection of primary antibody binding towards blotted platelet protein was done with horse-radish peroxidase (HRP) conjugated secondary antibody. Proteoforms immunodetected with HRP are shown in white. The red circle indicates the missing spots at pI 6.79 in this particular probe. In two other biological replicates of PGI_2 treated platelet samples these spots were detectable, like the presented PGI_2-treated platelet sample from figure 5 B1a, B1b and B1c.

Supplementary Figure S9

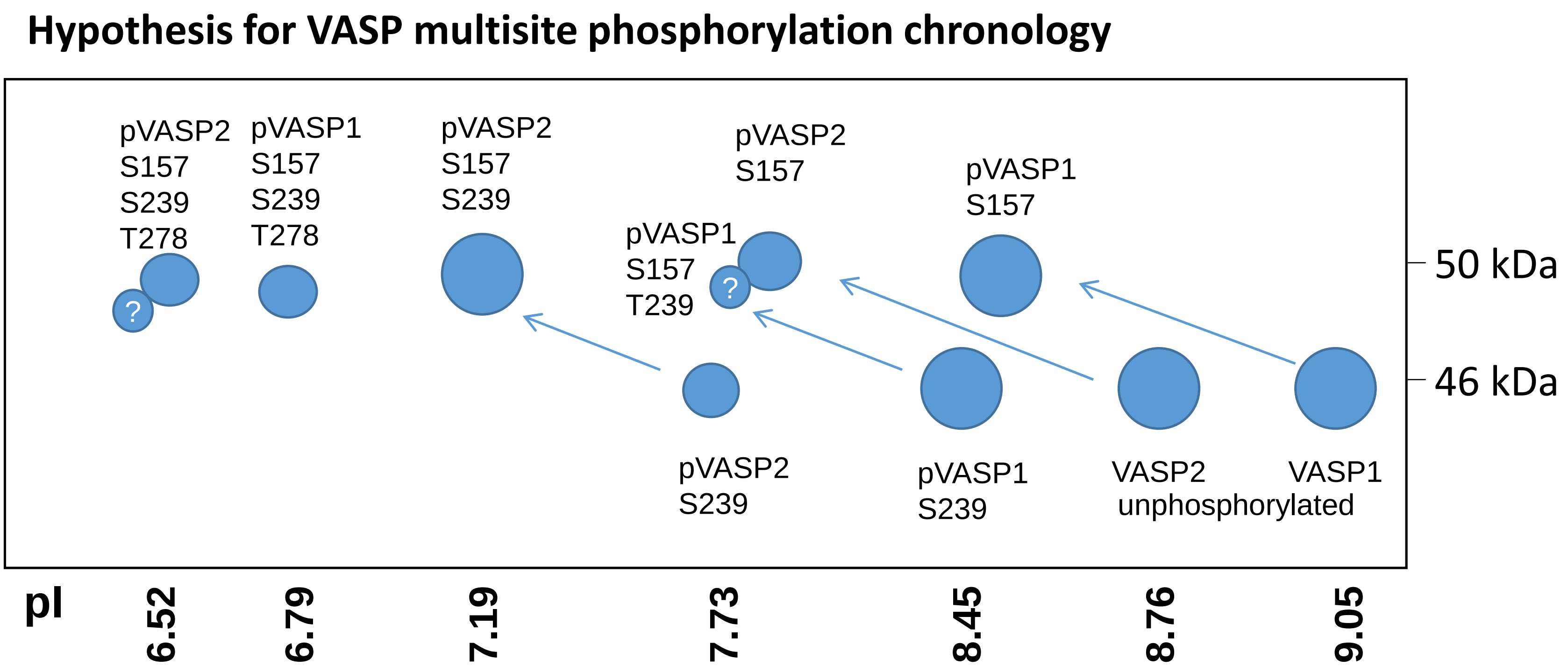

Supplementary Figure S9. Model for VASP multisite phospho-proteoforms after platelet inhibition by PGI_2 treatment

The two observed dephospho-forms of VASP can be explained by combinatory acetylation at positions 2 and 286 as describend by Uniprot (https://www.uniprot.org/uniprot/P50552). These two proteoforms lead to the complex phospho protein spot pattern in 2D PAGE. Phosphorylation at S157 results in the MW shift from 46 to 50 kDa.

Supplementary Figure S10

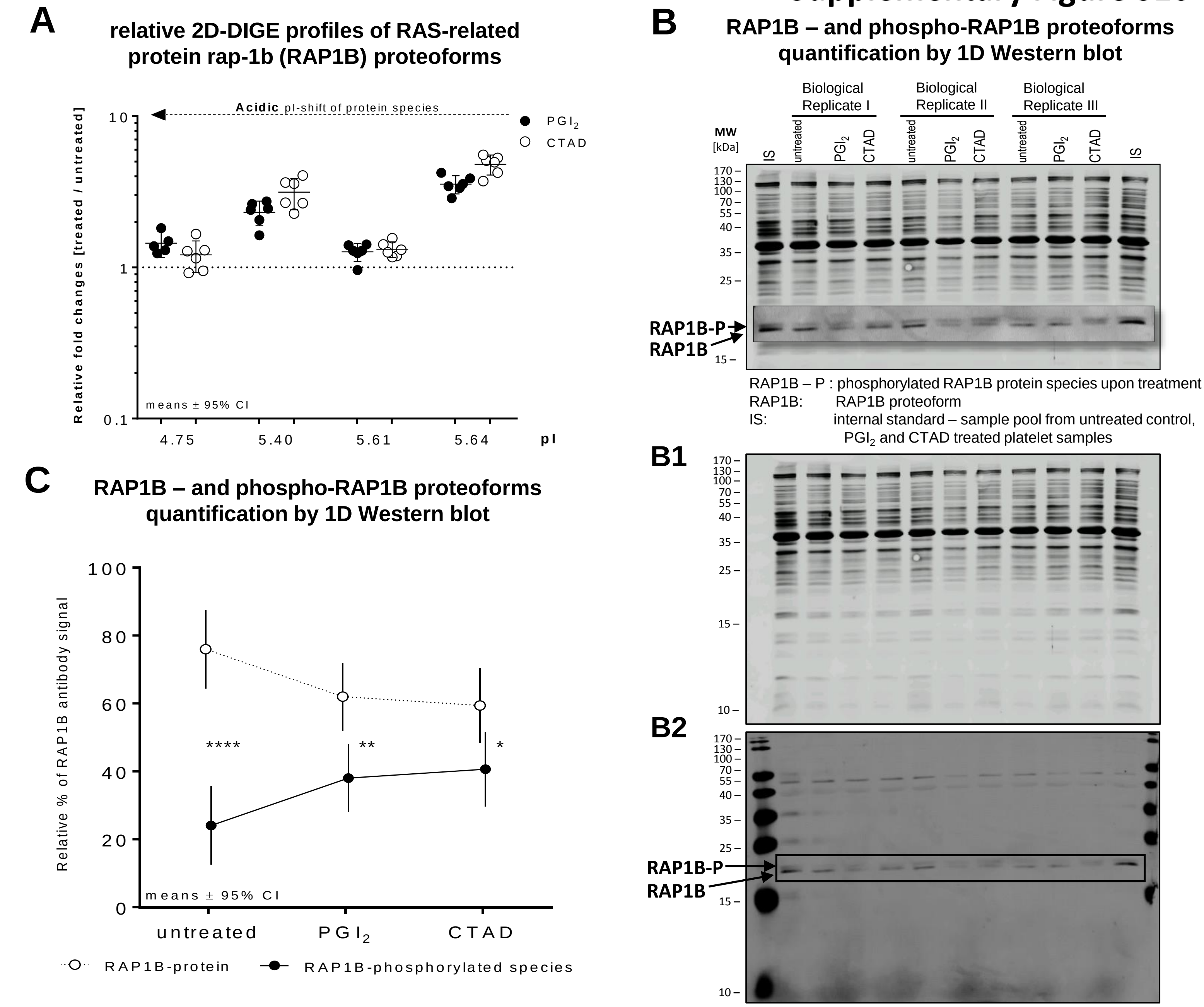

Supplementary Figure S10. Validation of platelet RAP1B - protein abundance and phosphorylation profile determined by 1D-Western blot. The 2D-DIGE analysis revealed a considerable change in phosphorylation of RAP1B species upon PGI_2-mediated inhibition. (**A**) Significantly changed RAP1B proteoforms identified by 2D-DIGE analysis are depicted along their pI range. Values are given as relative fold change between treated gel-filtered platelets (PGI_2, CTAD) and untreated control (dashed line y = 1) with their means and 95% confidence interval (CI), n = 6 (PGI_2, CTAD). However, since the acidic shift of phosphorylated RAP1B spots was different from those seen e.g. in LASP1, ZYX, SKAP2 and VASP (**Figure 4**), the phosphorylation of RAP1B proteoforms was validated via 1D-Western blot analysis. (**B**) Representative protein profiles and (phospho-)RAP1B protein species present in untreated, PGI_2- or CTAD-treated platelets presented as composite image made of B1 and B2. Platelet proteins from 3 out of 6 individuals (biological replicate I-III) were separated by 15% SDS-PAGE (12µg protein per lane), electroblotted onto NC-membranes and protein profiles were visualized by ion-based ruthenium whole-protein stain (RuBPS) to assess for equal protein amount (**B1**). In addition, a loading control (internal standard, IS) across multiple Western Blot membranes, representing a mixture of all used samples, was also applied. Uncropped, pan-RAP1B-antibody immunostained blot is shown in (**B2**). Since no phosphosite specific antibodies for RAP1B (RAP1B Ser^{171} or Ser^{587}) were available, pan-RAP1B antibodies in conjunction with high-resolution SDS-PAGE were used to differentiate the RAP1B proteoforms based on their electrophoretic migration velocity. The polyclonal pan-RAP1B antibodies recognized two protein bands with molecular weight of about 24 kDa, well corresponding to the MW positions of the RAP1B protein-spots detected by 2D-DIGE analysis. The upper band was assigned to the phosphorylated RAP1B protein species (RAP1B-P) due to its higher molecular weight and deceleration in the electrophoretic separation[10]. The bands around 50kDa are considered as unspecific binding of this antibody. The intensity of the phospho-protein band increased upon inhibition (PGI_2, CTAD), along with a decrease of the lower RAP1B protein band. Here, we confirmed a protein modification in favor of the phosphorylated protein species after inhibition. (**C**) Densitometric analysis of RAP1B-proteoforms. Unaffected and phosphorylated protein species of RAP1B are depicted as percentages of the total detected antibody signal (Cy5 at 670 nm) in the untreated control, PGI_2 and CTAD group (n = 6). The intensity of the phospho-band increased upon inhibition (PGI_2, CTAD), along with a decrease of the lower RAP1B protein band. Here, we confirmed a protein modification in favour of the phosphorylated protein species after inhibition. Differences between subgroups (untreated, PGI_2 and CTAD) and protein bands (RAP1B and RAP1B-P) were analyzed by a two-way-ANOVA (interaction $p < 0.001$). Differences within subgroup were evaluated by a Student´s t-test with α-level set at 0.05. **** $p < 0.0001$, ** $p < 0.01$, * $p < 0.05$. *IS – Internal standard, PGI_2 – prostacyclin, CTAD – untreated, theophylline, adenosine, dipyridamole*

Supplementary Figure S11

A **Chemoluminescence 1D – Western blots**

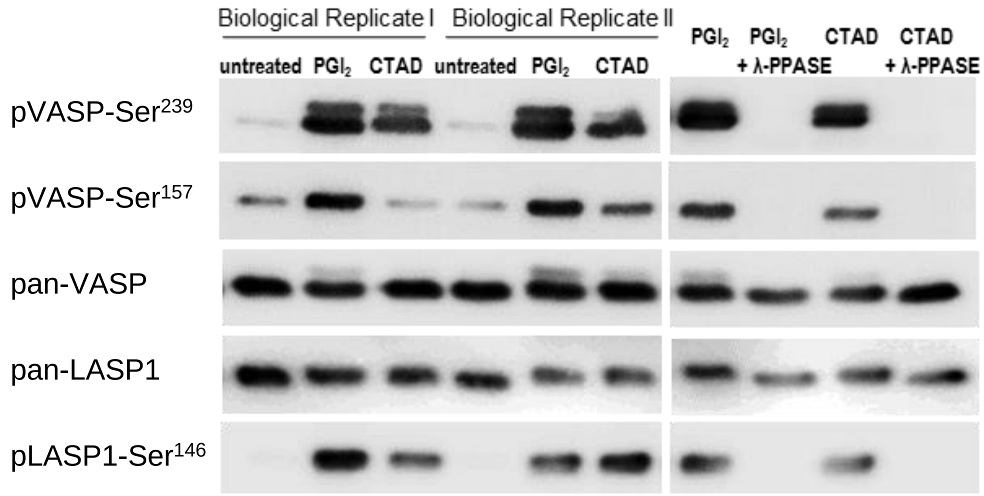

B **WB membrane with whole protein stain (RuBPS)**

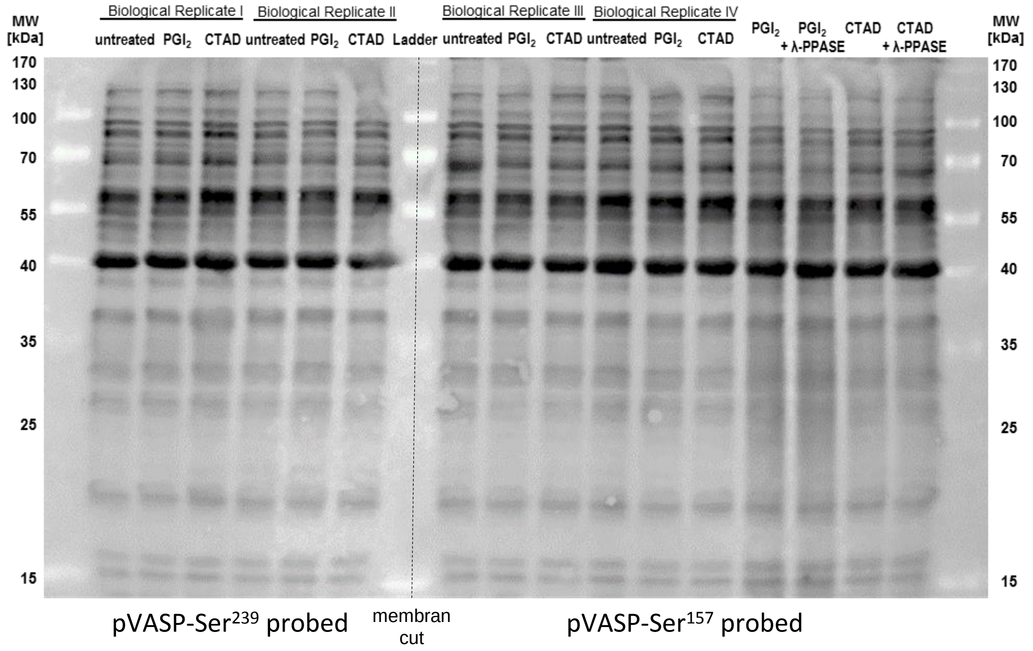

C **Full-length chemoluminescence images of 1D – Western blots**

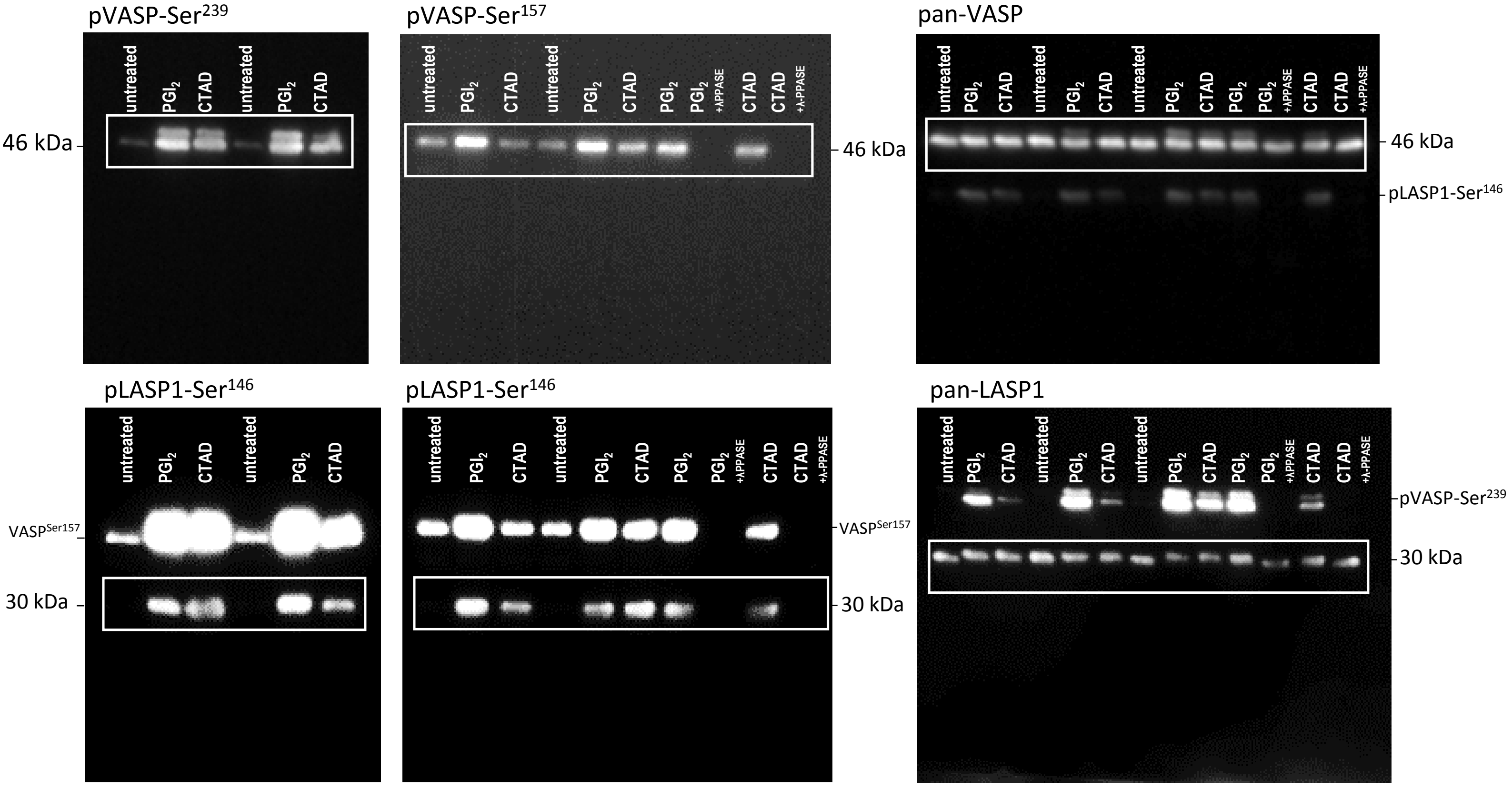

Supplementary Figure S11. Comparative 1D Western blot analysis of pVASP-Ser^{239}, pVASP-Ser^{157}, pan VASP, pLASP1-Ser^{146} and pan LASP1 proteoforms in platelets ensuing cAMP-induced PKA-signaling pathway. The 2D-DIGE analysis revealed a considerable stronger change in phosphorylation of the LASP1 spot with the pI 6.34 during inhibition as compared to several phosphorylated VASP spots. (**A**) For later translational steps into routine applications the phosphosite specific and pan protein levels of these spots were also characterized by conventional chemoluminescence 1D Western blotting. Representative 1D-Western Blot analysis (**A**), protein profiles (**B**) and (**C**) full-length 1D-Western Blot images obtained following pan- and site-specific antibody-incubations are shown for 2 individual (Biological Replicates I and II) untreated-, PGI_2- and CTAD-treated platelet proteomes (12µg protein/lane loaded) obtained following electrophoretic separation (12.5% SDS-PAGE) and electroblotting onto PVDF-membranes. To confirm phospho-reactivity of phospho-specific antibodies, pooled PGI_2 and CTAD-treated platelet protein samples were treated with λ-phosphatase (+ λ-PPASE) or buffer (right hand panels) (**A**) Cropped 1D-Western blot images obtained after incubations with P-VASP^{S239}, P-VASP^{S157}, pan-VASP, P-LASP1^{S146} and pan-LASP1 antibodies. (**B**) The Ion-based ruthenium (RuBPS) whole-protein stain of blotted proteins was performed to control for equal protein amount. (**C**) Full-length 1D-Western Blot chemiluminescent signals obtained after incubation with individual antibodies are shown. (**A**) and (**C**) The specific P-VASP^{S157} antibodies recognized a protein band with molecular weight of 50 kDa and the P-VASP^{S239} and pan-VASP antibodies recognised protein bands with 46 and 50 kDa, respectively. Pan-LASP1 and P-LASP1^{S146} antibodies recognised protein bands at 30 kDa. Of note, 1D-Western blot can not show the quantitative relations of LASP1 and VASP proteoforms to each other as it is possible with 2D-DIGE analysis. Thus, unphosphorylated LASP1 spots appear to be in higher abundance present in 2D-DIGE analysis compared to unphosphorylated VASP spots. The same phenomenon was detectable with the abundances of the phosphorylated VASP and LASP1 spots. However, as it is visible in 1D Western blots images (**C**), the used VASP antibodies seems to have a higher affinity to their antigens as compared to LASP1 antibodies. Thus, signals of phosphorylated VASP proteoforms were already detectable with P-VASP^{S239} and VASP^{S157} antibodies in untreated control platelets samples, whereas no signal of LASP1 phosphorylation was measureable with P-LASP^{S146} antibodies. Since no P-LASP1^{S146} band was detectable in untreated control samples, it was also not possible to perform comparative quantitative, densitometric evaluation of the degree in phosphorylation between VASP and LASP1 bands from untreated to PGI_2 or CTAD treated platelets.

PGI_2 – prostacyclin, CTAD - untreated, theophylline, adenosine, dipyridamole

Supplementary Figure S12

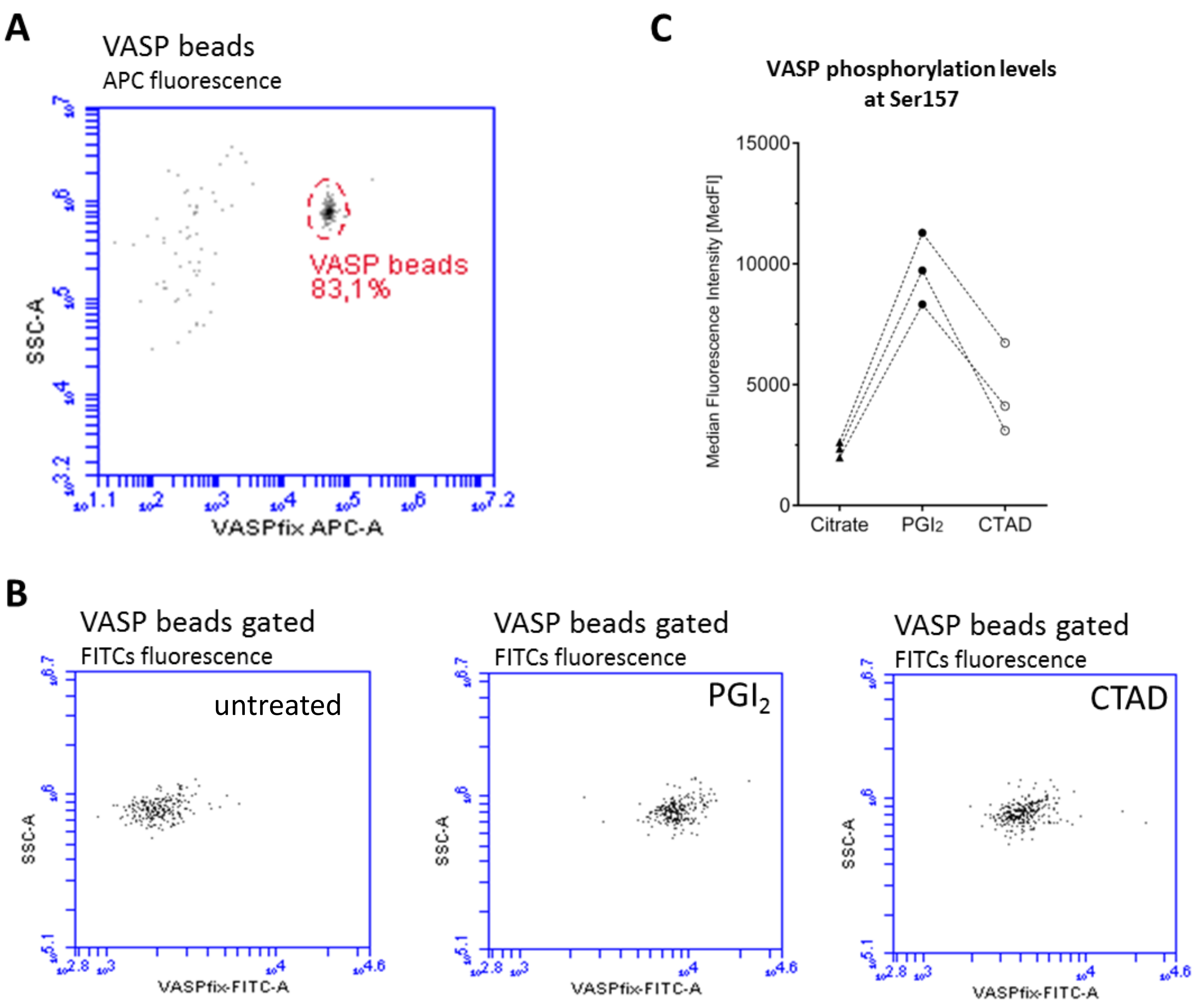

Supplementary Figure S12. Validation of inhibition-related VASP phosphorylation profiles by flow cytometry after PGI_2 and CTAD treatment. To validate our 2D-DIGE findings of a significant increase of VASP phosphorylation in PGI_2, but not in CTAD-treated platelets, we quantified the VASP-Ser157 phosphorylation by flow cytometry. (**A**) VASP-protein was detected in perforated gel-filtered platelets of untreated, PGI_2 and CTAD via allophycocyanin (APC-) fluorescence-labeled beads (exemplary plot of one measurement). (**B**) Gated VASP beads were then quantified via fluorescein isothiocyanate- (FITC) fluorescence-labeled antibodies at phosphorylation site VASP-Ser157 in the three treatment groups untreated, PGI_2 and CTAD. (**C**) Signals are quantitatively depicted as median fluorescence intensity (median FI) in three individuals and were compared by a Mann-Whitney-U test. The analysis showed a +4.21-FC median fluorescence intensity in PGI_2 treated platelets (p= 0.03) compared to +1.98-FC in CTAD (p = 0.44).

PGI_2 - prostacyclin, CTAD - untreated, theophylline, adenosine, dipyridamole, VASP - Vasodilator-stimulated phosphoprotein

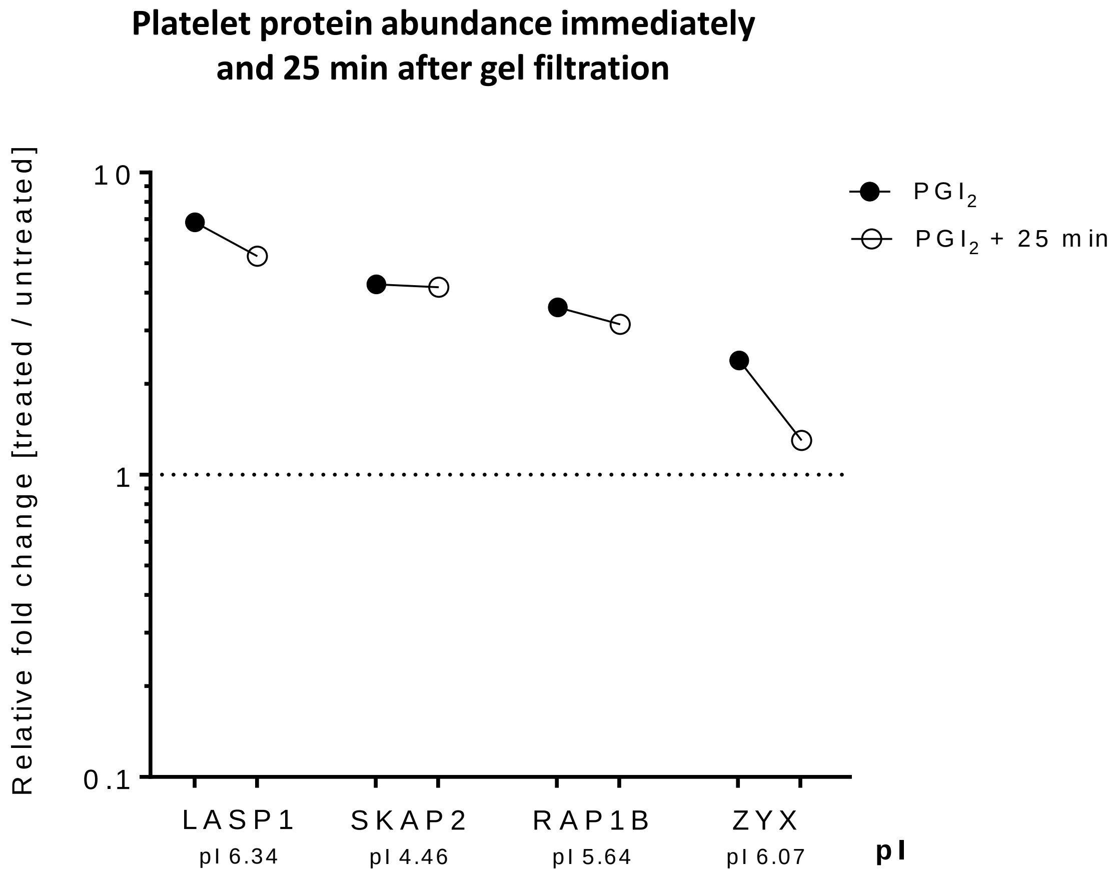

Supplementary Figure S13. Validation of phosphorylation stability of LASP1, SKAP2, RAP1B and ZYX protein species. Phosphorylation stability was determined in four known and potential cAMP/PKA targets. Protein species with the highest abundance in phosphorylation were selected. Here, PRP was incubated with 0.4 µM PGI_2 prior to gel-filtration. Obtained gel-filtered platelets were either precipitated immediately or left for another 25 minutes at room temperature before precipitation. Protein abundances were determined by 2D-DIGE analysis and normalized by the same internal standard as used in all previous experiments. Fold changes between spot abundances and citrated untreated control are depicted (n = 1).

PGI_2 – prostacyclin, cAMP/PKA - cyclic adenosine monophosphate / protein kinase A

Supplementary table S1 with all performed 2D-DIGE analysis and sample distribution

platelet samples	2D DIGE images	
	pH 4-7 (Cy3 or Cy5)	pH 6-9 (Cy3 or Cy5)
untreated controls (12 biological replicates)	12	12
inhibited by PGI_2 (6 biological replicates)	6	6
inhibited by PGI_2 + λ-PPase (1 biological replicate)	1	1
inhibited by CTAD (6 biological replicates)	6	6
inhibited by CTAD + λ-PPase (1 biological replicate)	1	1
activated by ADP (6 biological replicates)	6	6
activated by TRAP-6 (6 biological replicates	6	6

***Supplementary table S2*: Addition to table 1 with all significantly altered platelet proteins upon inhibition and activation.**

**A: Platelet Inhibition (n = 6)**					**Treated vs. untreated***			**PGI₂ vs. untreated †**			**CTAD vs. untreated †**			**λ-PPase‡(n=1)**		**MS§**
**Spot identification #**	**UniProt -Accession #**	**UniProt - Gene**	**Protein ID**	**Isoelectric point (pI)**	**Average fold change** (PGI₂, CTAD/untreated	**p- value** (mixed ANOVA)	**cor. p-value** (FDR<0.05)	**Fold change** (PGI₂/citrate)	**p-value** (subgroup comparison)	**cor. p-value** (FDR<0.05)	**Fold change** (CTAD/untreated)	**p- value** (subgroup comparison)	**cor. p-value** (FDR<0.05)	**Fold change** (PGI₂+λ-PPase/PGI₂)	**Fold change** (CTAD+λ-PPase/CTAD)	**Sequence Coverage (%)**
1	P60709	ACTB	Actin, cytoplasmic 1	5.16	**1.30**	2.8E-03	4-6E-03	**1.45**	8.9E-04	2.5E-03	**1.15**	2.2E-01	2.4E-01	1.10	0.84	9.0
2				5.18	**1.28**	1.6E-02	1.7E-02	**1.43**	6.2E-03	9.5E-05	**1.12**	4.7E-01	4.9E-01	1.08	0.78	73.1
3	P06733	ENO1	Alpha-enolase	6.60	**0.85**	7.6E-03	9.3E-03	**0.81**	2.2E-03	4.4E-03	**0.88**	4.5E-02	5.4E-02	0.90	0.80	18.9
4				6.70	**0.81**	4.1E-03	5.9E-03	**0.79**	1.6E-03	3.6E-03	**0.83**	9.5E-03	1.3E-02	1.20	0.73	48.4
5	P53396	ACLY	ATP-citrate synthase	7.15	**0.82**	1.8E-03	3.2E-03	**0.78**	4.9E-03	8.1E-03	**0.88**	2.2E-02	2.7E-02	1.68	1.34	30.0
6	Q05682	CALD1	Caldesmon	6.15	**0.53**	8.5E-05	4.0E-04	**0.54**	4.8E-05	3.0E-04	**0.55**	8.6E-04	2.5E-03	1.63	1.84	20.4
7				6.24	**0.54**	1.9E-04	7.0E-04	**0.54**	6.7E-05	3.7E-04	**0.53**	1.1E-03	2.8E-03	1.52	1.82	22.0
8				6.41	**0.55**	6.1E-05	3.4E-04	**0.57**	4.4E-05	3.0E-04	**0.54**	2.2E-03	4.4E-03	1.41	1.51	31.3
9				6.47	**0.79**	9.9E-04	2.2E-03	**0.81**	1.1E-03	2.8E-03	**0.76**	3.8E-03	6.6E-03	1.36	1.61	36.7
10	Q13976	PRKG1	cGMP-dependent protein kinase 1	6.72	**0.78**	7.6E-04	1.9E-03	**0.72**	2.4E-04	9.9E-04	**0.81**	5.1E-03	8.3E-03	2.06	2.21	67.1
11	P23528	CFL1	Cofilin-1	6.30	**0.70**	1.2E-03	2.4E-03	**0.71**	1.0E-01	1.1E-01	**0.68**	3.4E-04	1.2E-03	0.12	0.24	49.4
12	P51452	DUSP3	Dual specificity protein phosphatase 3	7.10	**1.40**	5.6E-03	7.4E-02	**1.45**	2.8E-03	5.4E-03	**1.33**	7.5E-03	1.1E-02	0.63	1.02	64.9
13	P60842	EIF4A1	Eukaryotic initiation factor 4A-I	5.44	**1.15**	1.2E-02	1.4E-02	**1.26**	5.5E-03	8.6E-03	**1.05**	5.9E-01	6.1E-01	1.12	0.98	33.7
14	P02679	FGG	Fibrinogen gamma chain	5.39	**1.15**	1.9E-05	1.6E-04	**1.25**	7.3E-06	7.4E-05	**1.05**	8.0E-02	9.1E-02	0.92	1.19	33.6
15				5.49	**1.10**	7.0E-03	8.7E-02	**1.22**	9.9E-03	1.3E-02	**0.96**	3.9E-01	4.1E-01	0.79	1.93	35.3
16	P06396	GSN	Gelsolin	5.77	**1.28**	4.4E-03	5.9E-03	**1.46**	1.7E-03	3.7E-03	**1.11**	1.9E-01	2.1E-01	0.95	0.56	33.6
17				6.10	**1.32**	8.0E-03	9.6E-03	**1.31**	6.5E-03	9.9E-03	**1.33**	5.2E-03	8.4E-03	0.71	1.63	9.1
18	P04406	GAPDH	Glyceraldehyde-3-phosphate dehydrogenase	7.52	**1.38**	1.4E-02	1.5E-02	**1.33**	2.1E-02	2.6E-02	**1.43**	5.9E-03	9.2E-03	1.05	0.75	26.6
19	Q9NR31	SAR1A	GTP-binding protein SAR1a	7.20	**0.74**	2.8E-03	4.6E-02	**0.74**	2.2E-03	4.4E-03	**0.75**	2.2E-02	2.7E-02	1.75	1.59	64.1
20	Q13418	ILK	Integrin-linked protein kinase	6.74	**1.54**	4.1E-03	5.9E-03	**1.56**	4.2E-02	5.1E-02	**1.51**	3.2E-03	5.7E-03	0.30	0.16	7.0
21				7.10	**1.45**	9.4E-05	4.1E-04	**1.43**	5.3E-03	8.4E-03	**1.47**	6.2E-05	3.7E-04	0.54	0.38	9.0
22	Q14847	LASP1	LIM and SH3 domain protein 1	6.34	**5.97**	1.9E-11	1.3E-09	**6.73**	1.0E-11	1.3E-09	**5.22**	4.6E-11	3.0E-09	0.15	0.21	63.3
23				6.60	**0.42**	3.7E-05	2.7E-04	**0.32**	2.6E-05	2.0E-04	**0.53**	7.4E-03	1.1E-02	3.13	2.34	50.6
24				6.83	**0.57**	1.2E-04	4.7E-03	**0.51**	4.8E-05	3.0E-04	**0.63**	3.1E-04	1.2E-03	1.70	1.69	21.0
25	P07195	LDHB	L-lactate dehydrogenase B chain	5.72	**1.18**	1.4E-02	1.5E-02	**1.32**	7.6E-03	1.1E-02	**1.04**	7.3E-01	7.4E-01	0.92	1.31	24.0
26	Q15555	MAPRE2	Microtubule-associated protein RP/EB	5.40	**1.35**	3.4E-03	5.5E-03	**1.53**	1.2E-03	3.0E-03	**1.17**	2.0E-01	2.2E-01	0.68	0.70	18.3
27	P26038	MSN	Moesin	6.67	**1.43**	1.3E-03	2.5E-03	**1.57**	1.6E-03	3.6E-03	**1.29**	1.3E-02	1.7E-02	1.13	0.99	45.4
28				6.86	**0.66**	2.1E-04	7.3E-04	**0.57**	2.3E-04	9.8E-04	**0.75**	4.5E-03	7.6E-03	2.88	2.56	48.5
29				7.12	**1.28**	7.8E-05	4.0E-04	**1.30**	4.1E-05	3.0E-04	**1.26**	1.4E-04	6.4E-04	0.95	0.79	56.8
30	P55209	NAP1L1	Nucleosome assembly protein 1-like 1	4.46	**0.81**	7.5E-04	1.9E-03	**0.79**	3.9E-04	1.4E-03	**0.82**	1.1E-03	2.8E-03	1.01	0.95	
31				4.51	**0.33**	2.5E-08	5.5E-07	**0.30**	1.5E-08	4.0E-07	**0.35**	4.8E-08	1.1E-06	0.81	0.70	10.0¶
32				4.59	**0.39**	7.6E-07	8.4E-06	**0.36**	5.3E-07	8.7E-06	**0.42**	3.4E-04	1.2E-03	5.96	6.29	
33	O00151	PDLIM1	PDZ and LIM domain protein 1	6.38	**1.85**	4.1E-03	5.9E-03	**1.93**	3.0E-03	5.6E-03	**1.73**	2.6E-03	5.0E-03	0.56	0.45	7.9
34	P08567	PLEK	Pleckstrin	6.47	**0.65**	8.2E-03	9.7E-03	**0.64**	4.1E-03	7.0E-03	**0.67**	9.6E-03	1.3E-02	0.37	0.21	9.1
35				6.60	**0.65**	3.7E-03	5.7E-03	**0.63**	1.9E-03	4.1E-03	**0.67**	4.8E-03	8.0E-03	0.59	0.54	22.2
36	P35813	PPM1A	Protein phosphatase 1A	5.16	**1.32**	4.6E-05	2.9E-04	**1.47**	1.3E-05	1.1E-04	**1.16**	1.5E-02	2.0E-02	0.75	0.94	42.7¶
37				5.26	**1.19**	4.8E-05	2.9E-04	**1.32**	4.6E-04	1.6E-03	**1.05**	2.3E-01	2.4E-01	1.03	1.25	

A: Continued

Spot identification #	UniProt -Accession #	UniProt - Gene	Protein ID	Isoelectric point (pI)	Treated vs. untreated [*]<br>**Average fold change** (PGI₂, CTAD/untreated)	**p- value** (mixed ANOVA)	**cor. p-value** (FDR<0.05)	PGI_2 vs. untreated [†]<br>**Fold change** (PGI_2/citrate)	**p-value** (subgroup comparison)	**cor. p-value** (FDR<0.05)	CTAD vs. untreated[†]<br>**Fold change** (CTAD/untreated)	**p- value** (subgroup comparison)	**cor. p-value** (FDR<0.05)	λ-PPase[‡](n=1)<br>**Fold change** (PGI_2+λ-PPase/PGI_2)	**Fold change** (CTAD+λ-PPase/CTAD)	MS[§]<br>**Sequence Coverage (%)**
38	P61224	RAP1B	Ras-related protein Rap-1b	4.75	**1.28**	4.2E-03	5.9E-03	**1.45**	1.3E-03	3.2E-03	**1.21**	5.9E-02	7.0E-02	1.05	1.11	30.4
39				5.40	**1.54**	6.6E-04	1.8E-03	**1.38**	7.8E-03	1.1E-02	**1.70**	1.9E-04	8.4E-04	0.87	0.72	80.4
40				5.61	**1.29**	1.3E-03	2.5E-03	**1.27**	2.0E-03	4.3E-03	**1.32**	6.4E-04	1.9E-03	0.98	0.94	17.9
41				5.64	**4.18**	6.7E-07	8.4E-06	**3.56**	2.8E-06	3.1E-05	**4.81**	1.8E-06	2.4E-05	0.44	0.27	83.2
42	Q9NS28	RGS18	Regulator of G-protein signaling 18	6.51	**0.71**	6.0E-04	1.8E-03	**0.70**	4.3E-04	1.5E-03	**0.71**	5.7E-04	1.8E-03	0.54	0.51	21.0[¶]
43				7.27	**1.32**	4.4E-03	5.9E-03	**1.32**	3.5E-03	6.2E-03	**1.31**	3.1E-03	5.7E-03	0.56	0.66	
44	O75563	SKAP2	Src kinase-associated phosphoprotein 2	4.46	**4.73**	1.2E-03	2.4E-03	**4.82**	6.8E-05	3.7E-04	**4.65**	1.7E-05	1.4E-04	0.35	0.23	33.7¶
45				4.51	**4.63**	8.0E-10	2.6E-08	**4.74**	6.9E-10	3.0E-08	**4.51**	9.8E-10	3.2E-08	0.30	0.20	
46				4.55	**3.45**	9.9E-08	1.6E-06	**3.65**	6.5E-08	1.2E-06	**3.25**	8.1E-06	7.6E-05	0.25	0.31	
47				4.59	**1.31**	1.0E-06	9.4E-06	**1.33**	5.9E-07	8.7E-06	**1.28**	2.0E-06	2.4E-05	1.09	0.85	
48	P50552	VASP	Vasodilator-stimulated phosphoprotein	7.19	**1.27**	6.0E-03	7.8E-03	**1.45**	2.9E-03	5.5E-03	**1.09**	2.3E-01	2.4E-01	0.83	0.78	6.6
49				7.73	**1.32**	3.7E-04	1.2E-03	**1.50**	1.2E-04	5.9E-04	**1.14**	7.5E-02	8.6E-02	0.37	0.41	24.0
50				8.45	**1.09**	8.4E-03	4.6E-02	**1.13**	1.3E-02	2.6E-02	**1.05**	6.3E-01	9.1E-01	0.68	0.73	34.4
51				8.76	**0.70**	1.8E-03	3.2E-03	**0.60**	5.3E-04	1.7E-02	**0.81**	6.3E-02	7.3E-02	2.05	1.13	23.7
52				9.05	**0.77**	9.2E-04	2.2E-03	**0.67**	2.7E-04	1.1E-03	**0.86**	6.2E-02	7.2E-02	1.93	1.08	36.1
53	Q15942	ZYX	Zyxin	6.01	**1.75**	1.9E-03	3.3E-03	**1.69**	2.5E-03	4.9E-03	**1.81**	9.7E-04	2.7E-03	0.41	0.36	9.4
54				6.07	**1.70**	1.1E-04	4.5E-04	**1.68**	1.4E-04	6.4E-04	**1.72**	8.4E-05	4.4E-04	0.57	0.89	29.4
55				6.18	**1.35**	1.1E-03	2.3E-03	**1.33**	1.4E-03	3.3E-03	**1.37**	6.4E-04	1.9E-03	0.85	0.93	26.4
56				6.33	**0.82**	1.3E-02	1.5E-02	**0.84**	1.6E-02	2.1E-02	**0.79**	3.2E-03	5.7E-02	1.23	1.32	61.2
57				6.50	**0.78**	1.5E-02	1.6E-02	**0.80**	1.8E-02	2.3E-02	**0.76**	6.8E-03	1.0E-02	1.65	1.79	52.8
58			Not successfully identified	4.80	**1.20**	3.6E-03	5.7E-03	**1.32**	1.2E-03	3.0E-03	**1.07**	2.4E-01	2.5E-01			
59				5.01	**1.36**	3.5E-04	1.2E-03	**1.22**	1.6E-02	2.1E-02	**1.51**	9.6E-05	4.9E-04			
60				5.24	**1.16**	1.7E-02	1.7E-02	**1.25**	1.0E-02	1.3E-02	**1.07**	4.4E-02	5.3E-02			
61				5.39	**1.16**	9.5E-04	2.2E-03	**1.30**	7.1E-04	2.1E-03	**1.01**	9.2E-01	9.3E-01			
62				5.85	**1.18**	1.5E-02	1.6E-02	**1.34**	9.6E-03	1.3E-02	**1.02**	9.3E-01	9.3E-01			
63				6.28	**0.92**	1.8E-02	1.8E-02	**0.82**	1.1E-01	1.2E-01	**1.02**	6.2E-01	6.3E-01			
64				6.33	**0.74**	9.9E-04	2.2E-03	**0.72**	4.8E-04	1.6E-03	**0.76**	1.6E-03	3.6E-03			
65				6.42	**0.57**	6.3E-03	8.0E-03	**0.65**	1.8E-02	2.3E-02	**0.50**	2.2E-03	4.4E-03			
66				8.01	**1.34**	6.4E-04	1.8E-03	**1.41**	8.8E-03	1.2E-02	**1.29**	1.4E-03	3.3E-03			

**B: Platelet activation (n = 6)**					**Treated vs. untreated** *			**ADP vs. untreated** †			**TRAP-6 vs. untreated** †			**λ-PPase**‡ **(n=1)**		**MS**§
**Spot identification #**	**UniProt - Accession #**	**UniProt - Gene**	**Protein ID**	**Isoelectric point (pI)**	**Average fold change** (ADP, TRAP-6/untreated)	**p- value** (mixed ANOVA)	**cor. p-value** (FDR<0.05)	**Fold change** (ADP/citrate)	**p-value** (subgroup comparison)	**cor. p-value** (FDR<0.05)	**Fold change** (TRAP-6/untreated)	**p- value** (subgroup comparison)	**cor. p-value** (FDR<0.05)	**Fold change** (PGI_2+λ-PPase/PGI_2)	**Fold change** (CTAD+λ-PPase/CTAD)	**Sequence Coverage (%)**
67	P22695	UQCRC2	Cytochrome b-c1 complex subunit 2	7.81	**0.82**	8.0E-05	1.4E-04	**0.85**	4.1E-04	7.9E-04	**0.79**	6.8E-03	7.9E-03	1.24	1.19	19.2
21	Q13418	ILK	Integrin-linked protein kinase	7.10	**0.80**	1.0E-04	1.4E-04	**0.72**	3.0E-05	1.7E-04	**0.89**	2.5E-02	2.7E-02	0.30	0.16	9.0
68	P48735	IDH2	Isocitrate dehydrogenase	8.37	**0.74**	9.7E-05	1.4E-04	**0.74**	9.0E-05	2.5E-04	**0.74**	5.5E-05	1.9E-04	1.11	1.05	12.0
69	P24844	MYL9	Myosin regulatory light polypeptide 9	4.74	**1.31**	3.1E-04	3.6E-04	**1.23**	5.1E-03	6.6E-03	**1.39**	5.2E-03	6.6E-03	1.15	0.84	41.3
70	P08567	PLEK	Pleckstrin	6.52	**1.65**	9.9E-05	1.4E-04	**1.76**	4.5E-04	7.9E-04	**1.53**	2.3E-04	5.4E-04	0.32	0.21	28.6
35				6.60	**1.60**	2.7E-05	1.4E-04	**1.62**	2.0E-05	1.7E-04	**1.58**	3.7E-05	1.7E-04	0.59	0.54	22.0
71			Not successfully identified	6.79	**0.86**	3.1E-03	3.1E-03	**0.96**	3.6E-01	3.6E-01	**0.76**	4.0E-03	6.2E-03			

* Mixed ANOVA (PGI_2 vs. CTAD vs. untreated and ADP vs. TRAP-6 vs. untreated)
† Alterations within subgroups by post-hoc contrasts (PGI_2, CTAD vs untreated) (ADP, TRAP-6 vs. untreated)
‡ λ-phosphatase-treated (λ-PPase+PGI_2, λ-PPase+CTAD) and λ-phosphatase-untreated gel-filtered platelets (PGI_2, CTAD)
§ Protein sequence coverage obtained by mass spectrometry
¶ Sequence coverage of protein species tryptically digested as pool

The tables summarize alphabetically (*Protein-ID*) altered 2D-DIGE protein spots with their corresponding protein abundance changes upon (**A**) inhibition (PGI_2/CTAD) and (**B**) activation (ADP/TRAP-6). Proteins of interest are given with their spot identification number in the exemplary 2D-DIGE image (*Supplementary Figure S3*) and their UniProt characteristics. Proteins of interest were defined as (a) matched in 90% of all gels and (b) average fold change > 20% between at least one treatment condition and untreated control (PGI_2 and/or CTAD vs. untreated; ADP and/or TRAP-6 vs. untreated control) and c) a FDR-corrected p-value < 0.05 obtained by a mixed ANOVA. Subgroup differences between treated gel-filtered platelets (PGI_2, CTAD, ADP and TRAP-6) and untreated control are given as relative fold changes. P-values corrected for multiple comparisons were obtained by post-hoc contrasts between the respective treatment subgroup and untreated controls. Fold changes > 20% between λ-phosphatase-treated (λ-PPase+PGI_2, λ-PPase+CTAD) and –untreated sample (PGI_2, CTAD) are considered as relevantly phosphorylated. Additionally, the protein sequence coverage obtained by mass spectrometry is given. The probability score used for the protein search algorithm was set at p < 0.05.

PGI_2 - prostacyclin, CTAD - citrate, theophylline, adenosine, dipyridamole, ADP - adenosine-diphosphate, TRAP-6 – thrombin receptor-activating peptide-6, λ-PPase - lambda-phosphatase, MS - mass spectrometry

Supplementary Material and Methods

Chemicals

Adenosine diphosphate (**ADP**), prostacyclin (**PGI_2**), Thrombin receptor-activating peptide-6 **(TRAP-6**), Iodoacetamide **(IAA**) were all purchased from Sigma Aldrich, St. Louis, MO, USA; **CTAD** blood tubes (3.5 ml, 0.129 mM trisodium citrate, 15 mM theophylline, 3.7 mM adenosine, 0.198 mM dipyridamole) and 0.129 mM **Sodium-citrate** blood tubes were obtained from Greiner Bio-One, Kremsmünster, Austria; Bradford Coomassie Plus kit (Pierce Biotechnology, USA); Dithiothreitol (**DTT**), Merck, Germany; Econo-Pac Chromatography Columns 15 mm Bio Rad, USA; Sepharose-2B (GE Healthcare, Uppsala, Sweden); calcium-free Dubecco's **PBS**-buffer (GIBCO, Paisley, Scotland, UK); **TCA** (Trichloracetic acid 6.1N solution, Sigma Aldrich, St. Louis, MO, USA)

Antibodies and enzymes

Mouse Anti-phospho-VASP (Ser^{239}), #0047-100, clone 16C2 (nanoTools Antikörpertechnik, Germany), 1:200 dilution for Western blot analysis

Mouse Anti-phospho-VASP (Ser^{157}), #0085-100, clone 5C6 (nanoTools Antikörpertechnik, Germany), 1:200 dilution for Western blot analysis

Rabbit Anti-VASP, #3112 (Cell Signaling, Germany), 1:1000 dilution for Western blot analysis

Rabbit Anti-phospho-LASP1 (Ser^{146}), (purchased from immunoGlobe Antikörpertechnik, Germany in order of Elke Butt), 1:100 dilution for Western blot analysis

Mouse Anti-LASP1, clone B8 (nanoTools Antikörpertechnik, Germany), 1:500

Goat Anti-panRAP1b, #SC-1481, clone B2508 (Santa Cruz Biotechnology Inc., CA, USA), 1:250 dilution for Western blot analysis

Goat Anti-Rabbit IgG (H+L)-HRP conjugate, #70745 (Cell Signaling, Germany), 1:20000 dilution for Western blot analysis

Donkey Anti-Mouse IgG (H+L)-HRP conjugate, #715-036-150 (Jackson ImmunoResearch Laboratories Inc., USA), 1:20000 dilution for Western blot analysis

Donkey Anti-Rabbit IgG (H+L)-DyLight 650 conjugate, #NBP1-75634 (Novus Biologicals, USA), 1:500 dilution for Western blot analysis

Donkey Anti-Mouse IgG (H+L)-DyLight 650 conjugate, #NBP1-75614 (Novus Biologicals, USA), 1:500 dilution for Western blot analysis

Lambda phosphatase and reaction buffer, #P0753S (New England BioLabs, MA, USA)

Trypsin, #PRV5111 (Promega, WI, USA)

VASP fix, #PSR-003 ("Platelet Solutions", Nottingham, UK),

CD62P, #304905 (anti-human CD62P, clone AK4, BioLegend, CA, USA)

Platelet proteome analysis by fluorescence two-dimensional gel electrophoresis (2D-DIGE)

For the acidic side, 36 µg protein (2 x 12 µg sample + 1 x 12 µg internal standard) were passively rehydrated on 24 cm pH 4-7 IPG-Dry-Strips in a solution containing 7 M urea, 2 M thiourea, 4 % CHAPS, 70 mM DTT and 0.5 % ampholyte pH 4–7. For the alkaline side, 24 cm pH 6-9 IPG-Dry-Strips

(GE Healthcare, Uppsala, Sweden) were soaked in a rehydration solution containing 7 M urea, 2 M thiourea, 4 % CHAPS, 150 mM DTT and 2 % ampholytes pH 6–10 prior to isoelectric focusing. Afterward, 3 x 12 µg of labeled protein samples were applied by “cup-loading” via the acidic side and the isoelectric focusing was carried out until 30 kVh was reached. The following SDS-PAGE was performed with an 11.5 % acrylamide gel at 35 V for 1 hour, 50 V for 1.5 hours and 110 V for 16.5 hours at 10˚C in an Ettan DALTsix electrophoresis chamber (GE Healthcare, Uppsala, Sweden). The gels were scanned afterward with a resolution of 100 µm using a Typhoon 9410 imager (GE Healthcare, Uppsala, Sweden).

Analysis of platelet activation by flow cytometry

To monitor platelet activation status, the commonly used platelet surface activation marker CD62P was determined. Therefore, untreated and treated (PGI_2, CTAD, ADP, TRAP-6) gel-filtered platelets were incubated with a phycoerythrin (PE)-labeled antibody against CD62P (dilution 1:40) for 20 minutes. Afterward, platelets were fixed with paraformaldehyde (1 % final concentration) and analyzed by flow cytometry using a BD Accuri flow cytometer and BD AccuriC6 analysis software (Becton Dickinson, NJ, USA). Read-outs are given as fold changes (treated vs. untreated). Basal CD62P expression of citrated GFP was considered as baseline (100 %). To detect differences between untreated controls and inactivation (PGI_2, CTAD) or activation (ADP, TRAP-6), respectively, a non-parametric one-sample Wilcoxen-rank sum test was performed. Significance level (two-sided) was set at $p < 0.05$ for all tests. SPSS Statistics 20.0 (IBM, Chicago, USA) was used.

VASP phosphorylation flow cytometry assay to validate 2D-DIGE results

To validate the phospho-profiles of VASP upon PGI_2 and CTAD treatment revealed by 2D-DIGE, the phosphorylation site Ser157 of VASP was assessed by flow cytometry. Blood samples from three healthy volunteers were freshly drawn into citrated and CTAD blood tubes and processed to generate gel-filtered platelets (as described above). Gel-filtered platelets from citrated, PGI_2 (0.4 µM) and CTAD treated platelets were subjected to the ready-to-use *VASPfix*- assay (“Platelet Solutions”, Nottingham, UK). 4 µl of gel-filtered platelets was forcefully mixed with 25 µl *VASPfix*-solution to perforate the platelet membrane and make VASP accessible[8]. After a one hour incubation in the dark, flow cytometry was performed (Accuri C6, Becton Dickinson). VASP-beads were detected by their allophycocyanin (APC-) fluorescence. VASP antibody against Ser^{157} was detected by its fluorescein isothiocyanate (FITC-) fluorescence on APC-gated beads. Values are represented as median fluorescence intensity (*Supplementary Figure S10*). SPSS Statistics 20.0 (IBM, Chicago, USA) was used.

2D-DIGE Image analysis

For spot detection and inter-gel matching the “DeCyder™ 6.5.14” software (GE Healthcare, Uppsala, Sweden) was used. Spot intensities were normalized to remove differences in staining intensities and to eliminate running differences between 2D gel images. The obtained values (Standardized

Abundance - SA) were subsequently used for the statistical analysis and are referred to as fold change (FC).

Biological pathway analysis

To gain a deeper insight into the biological function of differentially regulated proteins identified by 2D-DIGE, we initially applied Reactome pathway enrichment analysis (www.reactome.org) and found "Hemostasis" followed by "Platelet activation signaling and aggregation" as pathways with the highest number of overlaps and lowest p-value/FDR corrected p-value enriched (7.5E-11/3.3E-08, 10 proteins overlap and 7.02E-11/3.31E-08, 8 proteins overlap, respectively). Next, we employed an integrated biological database software-suite (Ingenuity Pathway Analysis, IPA; QIAGEN Inc., https://www.qiagenbioinformatics.com/products/ingenuity-pathway-analysis) in addition to extensive literature research. For the initial IPA-core analysis[9], proteins differentially regulated upon platelet inactivation (PGI_2 and CTAD, n = 26) compared to untreated control were entered along with their average fold change and the FDR-corrected p-value (for details see *Supplementary Table S1*) to enable weighting. The corresponding highest network score (45 with 17 focus molecules) included "Cell-to-Cell Signaling and Interaction", "Hematological System Development", and "Function and Inflammatory Response" within the term "Top Disease and Functions", where PKA was a key node and PKC was inferred manually based on literature[7]. Subsequently, datasets containing proteins and corresponding fold changes identified after λ-phosphatase treatment (PGI_2+λ-PPase, CTAD+λ-PPase) and corresponding controls (PGI_2, CTAD) were subjected to phosphorylation analysis to predict kinases and phosphatases within the upstream-regulators-module. By applying the Pathway Building tool, proteins that were differentially regulated by platelet activation (ADP and TRAP-6, n = 5), as well as the predicted kinases and phosphatases, were added and the initial network was narrowed to the platelet system. To give an additional layer of information, the Molecule Activity Predictor tool, which uses the protein level alterations within subgroups (PGI_2, CTAD, ADP, TRAP-6) was applied to suggest functional effects on neighboring molecules. For visualization, differentially regulated proteins identified upon inactivation (PGI_2 vs. untreated controls and CTAD vs. untreated controls), or activation (ADP vs. untreated controls and TRAP-6 vs. untreated controls), were alphabetically arranged in a circular layout and connected to the central nodes PKA and PKC, respectively.

Validation of RAP1B protein profiles by 1D Western blot

For the validation of RAP1B protein abundance differences, 1D Western blot analysis was performed. A total of 12 µg platelet protein of untreated control, PGI_2- and CTAD-treated gel-filtered platelets was mixed with a sample buffer (1.5 % SDS, 30 mM Tris-HCl pH 6.8, 7.5 % glycerol and 100 mM DTT), and separated by a 15 % SDS-PAGE (50 V for 30 minutes, 100 V for 5 hours). Subsequently, proteins were wet-blotted (75 V, 90 minutes) onto a nitrocellulose membrane (Pall, East Hills, NY USA) and blocked with 5 % non-fat dry milk (BioRad, Hercules, CA USA) in PBS containing 0.3 % Tween-20 (PBS-T). Ion-based ruthenium (Sigma-Aldrich St. Louis, MI USA) whole-protein stain was performed (dilution 1:100,000, overnight at 4°C) to control equal sample application and blotting procedure[9]. For validation, nitrocellulose membranes were incubated with a polyclonal pan-RAP1B (1:250 diluted in

PBS-T containing 3 % non-fat dry milk) for 2 hours at room temperature. After washing (3x PBS-T for 5 minutes, each), membranes were incubated with a Cy5-conjugated secondary antibody, diluted 1:500 in PBS-T containing 3 % non-fat dry milk and for 1.5 hours in the dark at room temperature. After washing (2x PBS-T, 1x 1xPBS for 5 minutes, each), the fluorescence signal was detected at 670 nm with a resolution of 100 µm using a Typhoon FLA9500 imager (GE Healthcare, Uppsala, Sweden). Quantification of detected RAP1B immune-reactive signals was performed by intensity measurements of each RAP1B- (24 kDa) and phosphorylated RAP1B-band (22 kDa) (ImageQuant TL 8.0, GE Healthcare, Uppsala, Sweden). RAP1B-immuno-bands are given as percentages of the total detected antibody signal in the citrate, PGI_2 and CTAD group. Differences between subgroups (untreated control, PGI_2 and CTAD) and antibody bands were analyzed by a two-way ANOVA. Differences within subgroup were evaluated by a Student´s t-test with an α-level set at $p < 0.05$. SPSS Statistics 20.0 (IBM, Chicago, USA) was used.

Detection of all proteoforms and phosphorylated protein species from VASP and LASP1 by 2D Western blot

For the detection of VASP and LASP1 proteoforms, 2D Western blot analysis was performed. For 2D Western blot analysis, 36 µg Cy2-labeled of untreated control, PGI_2- and CTAD-treated gel-filtered platelet proteins were separated as described for 2D-DIGE gels. Subsequently Cy2-labeled proteins were blotted (75 V, 90 minutes) onto a PVDF membrane (FluoroTrans® W, Pall Laboratory, East Hills, NY USA) and blocked with 5 % non-fat dry milk in PBS-T over night at 4°C. For detection, following antibodies were used in corresponding dilutions by incubation for 2 hours at room temperature in PBS-T containing 3 % non-fat dry milk: monoclonal pVASP-Ser^{239} (1:200), monoclonal pVASP-Ser^{157} (1:200), polyclonal pan-VASP (1:1000), pLASP-Ser^{146} (1:100) and with a monoclonal pan-LASP1 (1:500). After washing (3x PBS-T for 5 minutes, each), membranes were incubated with a horse-radish peroxidase (HRP) -conjugated secondary antibody, diluted 1:20,000 in PBS-T containing 3 % non-fat dry milk and incubated for 1.5 hours at room temperature. After washing (2x PBS-T, 1x 1xPBS for 5 minutes, each), the HRP signal was detected using an Enhanced Chemiluminescent (FluorChem® HD2, Alpha Innotech, CA USA).

Detection of all proteoforms and phosphorylated protein species from VASP and LASP1 by 1D Western blot

A total of 12 µg platelet protein of untreated control, PGI_2-, CTAD- and λ-phosphatase treated platelet proteins were mixed with a sample buffer (1.5 % SDS, 30 mM Tris-HCl pH 6.8, 7.5 % glycerol and 100 mM DTT), and separated by a 12.5 % SDS-PAGE (50 V for 20 minutes, 100 V for 3 hours). Subsequently, proteins were blotted (50 V for 30 minutes, 75 V for 90 minutes) onto a PVDF membrane and blocked with PBS-T containing 5 % non-fat dry milk. Ion-based ruthenium whole-protein stain was performed (dilution 1:100,000, overnight at 4°C)[10]. Afterwards, membranes were blocked with 5 % non-fat dry milk in PBS-T over night at 4°C. For Western blot analysis, membranes were incubated with p-$VASP^{Ser239}$, p-$VASP^{Ser157}$, pan-VASP, p-$LASP^{Ser146}$ and pan-LASP1 with the same antibody dilutions and conditions as described above. After washing, membranes were

incubated with a HRP-conjugated secondary antibody and the HRP signal was detected using an Enhanced Chemiluminescent.

References:

1 Han, X. *et al.* Zyxin regulates endothelial von Willebrand factor secretion by reorganizing actin filaments around exocytic granules. *Nat Commun* **8**, 14639, doi:10.1038/ncomms14639 (2017).

2 Zimman, A. *et al.* Phosphoproteomic analysis of platelets activated by pro-thrombotic oxidized phospholipids and thrombin. *PLoS One* **9**, e84488, doi:10.1371/journal.pone.0084488 (2014).

3 Ma, P. *et al.* Modulating platelet reactivity through control of RGS18 availability. *Blood* **126**, 2611-2620, doi:10.1182/blood-2015-04-640037 (2015).

4 Butt, E. *et al.* Actin binding of human LIM and SH3 protein is regulated by cGMP- and cAMP-dependent protein kinase phosphorylation on serine 146. *J Biol Chem* **278**, 15601-15607, doi:10.1074/jbc.M209009200 (2003).

5 Tyers, M. *et al.* Molecular cloning and expression of the major protein kinase C substrate of platelets. *Nature* **333**, 470-473, doi:10.1038/333470a0 (1988).

6 Marcus, K., Moebius, J. & Meyer, H. E. Differential analysis of phosphorylated proteins in resting and thrombin-stimulated human platelets. *Anal Bioanal Chem* **376**, 973-993, doi:10.1007/s00216-003-2021-z (2003).

7 Harper, M. T. & Poole, A. W. Isoform-specific functions of protein kinase C: the platelet paradigm. *Biochem Soc Trans* **35**, 1005-1008, doi:10.1042/BST0351005 (2007).

8 Glenn, J. R. et al. 'VASPFix' for measurement of VASP phosphorylation in platelets and for monitoring effects of P2Y12 antagonists. Thromb Haemost 111, 539-548, doi:10.1160/TH13-07-0581 (2014).

9 Kramer, A., Green, J., Pollard, J., Jr. & Tugendreich, S. Causal analysis approaches in Ingenuity Pathway Analysis. Bioinformatics 30, 523-530, doi:10.1093/bioinformatics/btt703 (2014).

10 Zellner, M. et al. Fluorescence-based Western blotting for quantitation of protein biomarkers in clinical samples. Electrophoresis 29, 3621-3627, doi:10.1002/elps.200700935 (2008).
